# Supplementary material for: Target identification for small-molecule discovery in the FOXO3a tumor-suppressor pathway using a biodiverse peptide library
Source: Cell Chem Biol. 2021 Nov 18;28(11):1602–1615.e9. doi: 10.1016/j.chembiol.2021.05.009 (PMC8610377; doi:10.1016/j.chembiol.2021.05.009)
Supplement: Document S1. Figures S1–S6 and Tables S1, S2, S4, and S5 [file mmc1.pdf]

Cell Chemical Biology, Volume *28*

Supplemental information

Target identification for small-molecule discovery in the FOXO3a tumor-suppressor pathway using a biodiverse peptide library

Amy Emery, Bryn S. Hardwick, Alex T. Crooks, Nadia Milech, Paul M. Watt, Chandan Mithra, Vikrant Kumar, Saranya Giridharan, Gayathri Sadasivam, Subashini Mathivanan, Sneha Sudhakar, Sneha Bairy, Kavitha Bharatham, Manjunath A. Hurakadli, Thazhe K. Prasad, Neelagandan Kamariah, Markus Muellner, Miguel Coelho, Christopher J. Torrance, Grahame J. McKenzie, and Ashok R. Venkitaraman

SUPPLEMENTAL INFORMATION

FIGURE LEGENDS

Supplementary Figure S1: Perturbation of the AKT and nuclear export pathways leads to an increase in nuclear localisation of GFP-FOXO3a in a U2OS reporter cell line (related to Figure 1) (A) Quantitation of nuclear translocation in U2OS-GFP-FOXO3a cells in response to small molecule inhibitors. Compounds underwent a two-fold serial dilution from a top concentration, on the left for each compound, of 1µM for BEZ235, GSK2334470, GSK69093 and PI-103, 20µM for LY294002 or 2nM for Leptomycin B. Grey bars are DMSO only. BEZ235: a dual phosphoinositide 3-kinase (PI3K)-mammalian target of rapamycin (mTOR) inhibitor. GSK2334470: PDPK1 inhibitor. GSK69063: pan-Akt kinase inhibitor. PI-103: ATP-competitive inhibitor of DNA-PK, PI 3-kinase (Class IA), and FRAP (mTOR) complex 1 and 2. LY294002: PI3K inhibitor. Leptomycin B: inhibitor of Exportin 1. (B) Quantitation of nuclear translocation in U2OS-GFP-FOXO3a cells following siRNA-mediated knockdown of selected AKT and nuclear export pathway components. Single siRNAs were used excepting hatched bars which indicate that a pool of four siRNAs was used. Grey bars are negative or non-targeting controls. Data represent the mean of three independent experiments +/- SD.

Supplementary Figure S2: Validation of the interaction between peptide 9J10 and 14-3-3 (related to Figure 2). (A) Western blotting for 14-3-3 in protein eluates following immunoprecipitation with V5 antibody from U2OS-GFP-FOXO3a cells transfected with the indicated peptides for 48hrs. These samples are the same as those shown in **Figure 2A**. (B) Western blotting for 14-3-3 following V5 immunoprecipitation with additional high stringency wash steps (400mM LiCl) prior to elution. (C) Amino acid sequence of peptide 9J10. (D) Western blotting for 14-3-3 following V5 immunoprecipitation in a panel of cell lines transfected with 9J10 or vector only. Asterisk marks the antibody light chain. With the exception of (A), all Western blots are representative of at least two independent replicates.

Supplementary Figure S3: Perturbation of the AKT/PI3K pathway with small molecule inhibitors in HEK293T cells leads to nuclear translocation of FOXO3a and changes in

pathway specific phosphorylation (related to Figure 2). (A) Representative images of HEK293T cells showing FOXO3a localisation after 3hr treatment with small molecule inhibitors. PI3Ki=1.25µM PI-103, AKTi=1µM GSK690693. The nuclear area is marked by an orange dotted line. Scale bar = 25µm. (B) Representative Western blots of cell lysates from HEK293T cells treated with small molecule inhibitors as in (A). Phosphorylation of AKT at Ser473 is dependent on PI3K activity. Phosphorylation of FOXO3a at Ser253 is carried out by AKT. Independent blots using the same samples were probed for the indicated targets. For each blot, HSP90 expression is shown on the right. (C) Quantitation of Western blots. Intensities were normalised to HSP90 intensities from the same blots then expressed relative to DMSO for each protein. Bars indicate the mean of three independent experiments +/- SD

Supplementary Figure S4: Expression of 9J10^{WT} but not 9J10^{AMut} modulates transcription and impairs cell growth (related to Figure 4) (A) List of over-represented transcription factors for genes regulated by 9J10 alone generated by promoter motif analysis using oPOSSUM-3. Transcription factors with a Z-score >10 and a Fisher score >7 were considered significant. (B) Cell confluency measurements from live cell imaging of MCF7 and (C) BT-549 cells transfected with Vector, 9J10^{WT} or 9J10^{AMut} at the final 48hr timepoint. Bars represent the average of three independent experiments +/- SD. (D) A representative experiment for MCF7 and (E) BT549 cells showing changes in cell confluency over time in cells transfected as in (B) and (C). Data represent the average of four fields +/- SD.

Supplementary Figure S5: Binding by ITC and co-crystal structure of 9J10 and 14-3-3ε (related to Figure 5). (A) Isothermal titration calorimetry demonstrating binding between a minimised 9J10 peptide and 14-3-3ε. A representative experiment is shown from two independent experiments. (B) The 2Fo-Fc map contour at 1 σ level around the 9J10 peptide, from the co-crystal structure. The map clearly shows the 9J10 peptide region. (C) Table showing interacting amino acids between 14-3-3ε protein and 9J10 peptide. (D) The Arg8 at pS-3 position of 9J10 (blue stick) shows significant conformational difference compared to that of Arg at the same position of the canonical phosphopeptides interacting with 14-3-3ε

(PDB: 2BR9 (yellow)); PDB: 6EIH-(orange)). (E) Table showing arginine pairing geometry from co-crystal structure.

Supplementary Figure S6: Identification and validation of compound CU7218 (related to Figure 6). (A) Optimisation of fluorescence polarisation assay. Direct binding of 14-3-3ε to TAMRA-labelled 9J10Min phosphopeptide. Kd value was calculated by titrating 14-3-3ε protein in the presence of 10nM TAMRA-p9J10Min. Plot represents mean of three independent experiments, +/- SD. (B) Competitive inhibition of TAMRA-p9J10Min binding to 14-3-3ε by an unlabelled 9J10 derived peptide measured by FP. Unlabelled 9J10 was titrated in the presence of 10nM TAMRA-p9J10Min. Plot represents mean of three independent experiments, +/- SD. (C) Graph showing Z' calculation for assay quality. Kd and IC_{50} values are the mean of three independent experiments, +/- SD.

Supplementary Figure S1: Perturbation of the AKT and nuclear export pathways leads to an increase in nuclear localisation of GFP-FOXO3a in a U2OS reporter cell line.

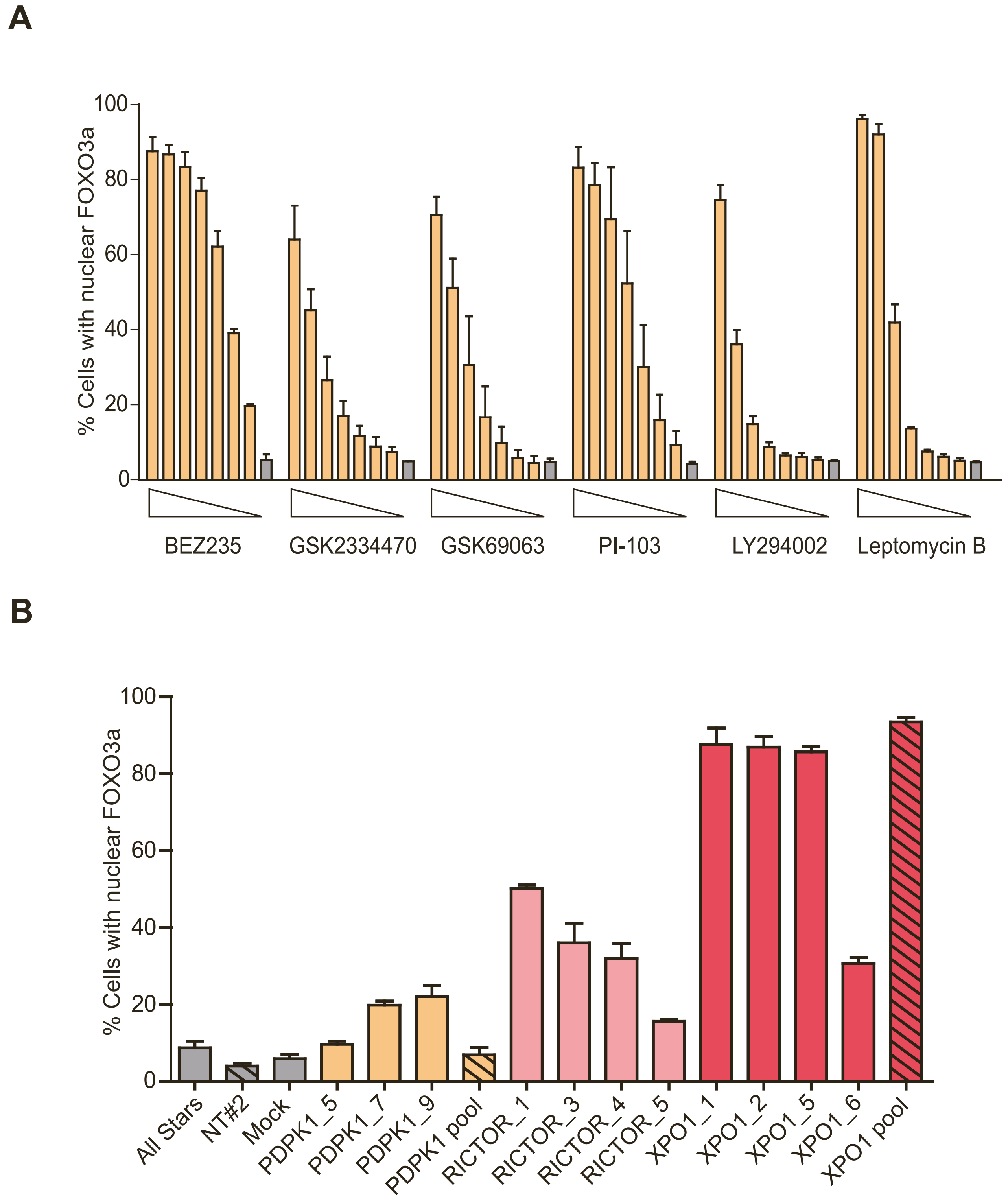

Supplementary Figure S2: Validation of the interaction between peptide 9J10 and 14-3-3.

A

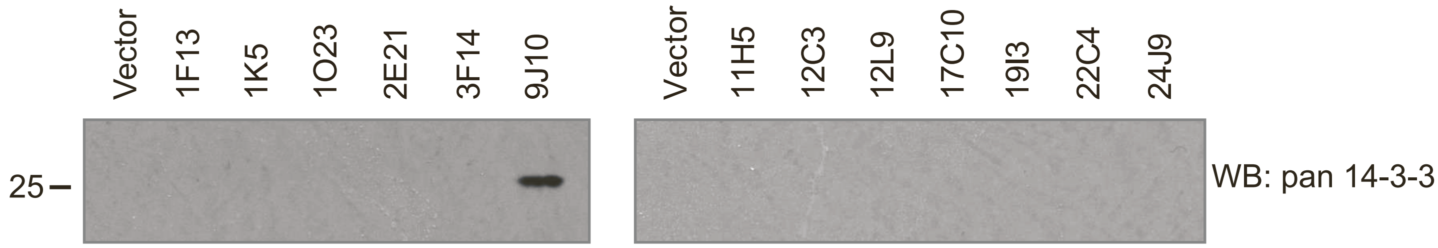

B

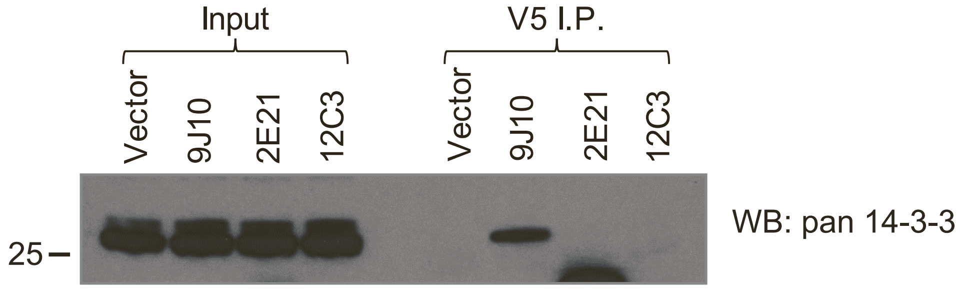

C

PTTSDNLLLSPELRDGARGAKRLCPEIGGDEAWRLLTEDAVAEQGEPVS
SVYLLRDPLRPSGRALGPVADQRGPPLAATAPIPLNRTPGRRRNSN

D

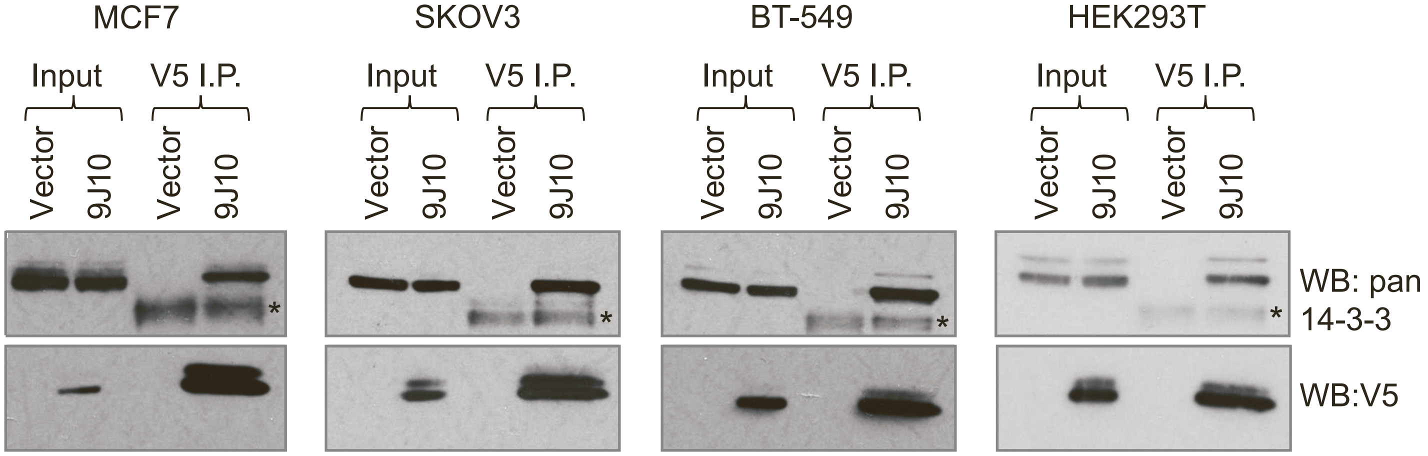

Supplementary Figure S3: Perturbation of the AKT/PI3K pathway with small molecule inhibitors in HEK293T cells leads to nuclear translocation of FOXO3a and changes in pathway specific phosphorylation.

A

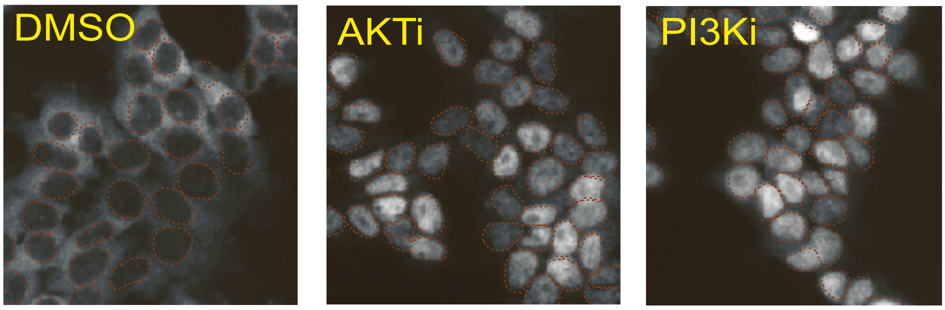

B

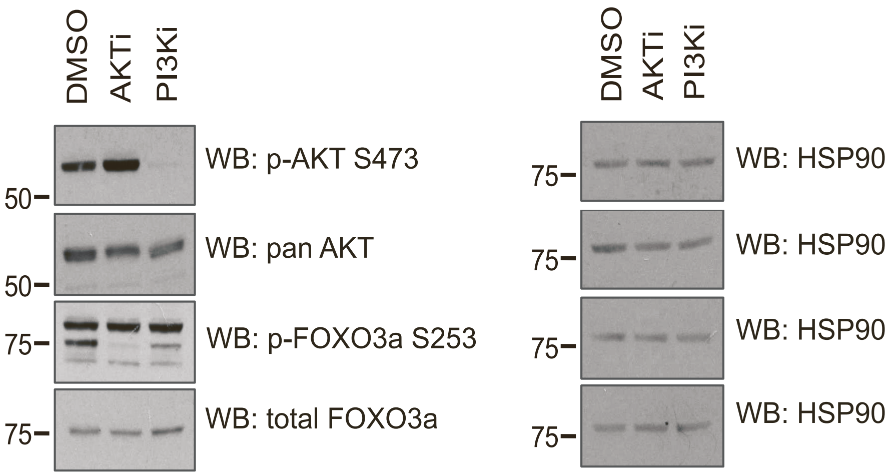

C

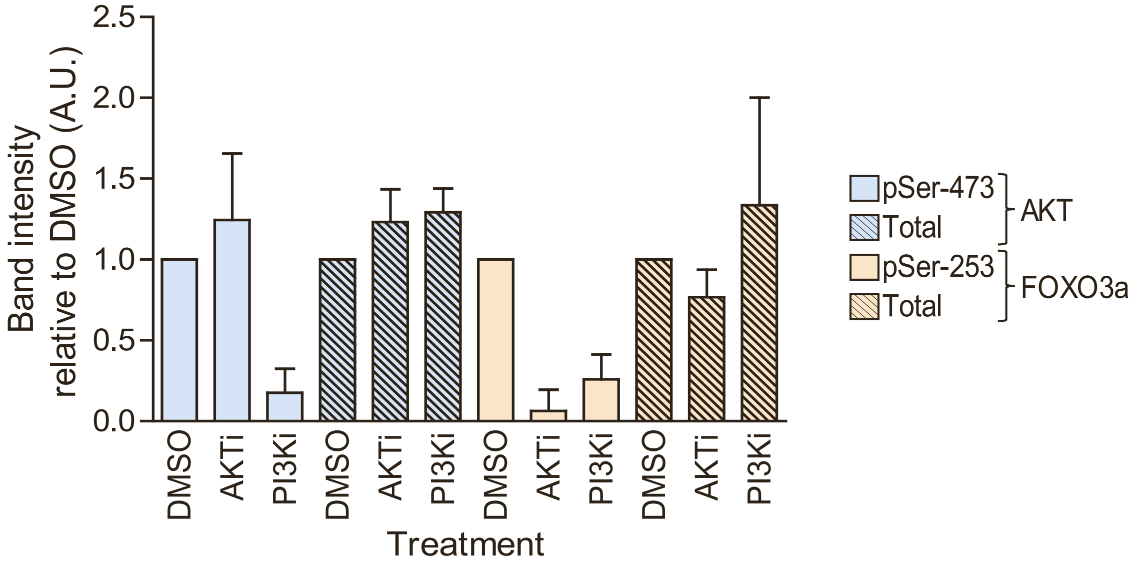

Supplementary Figure S4: Expression of 9J10^{WT} but not 9J10^{AMut} modulates transcription and impairs cell growth.

A

Transcription factor	Z-score	Fisher Score
EWSR1-FLI1	17.1	15.7
FOXI1	16.6	41.7
SRY	15.6	43.7
HOXA5	14.1	70.1
NFYA	13.8	69.7
ARID3A	13.5	47.7
TBP	13.1	37.3
Foxd3	12.4	38.5
Pdx1	12.3	46.2
Nkx2-5	11.7	42.7
Gfi	10.5	61.7
Foxq1	10.4	28.7

B

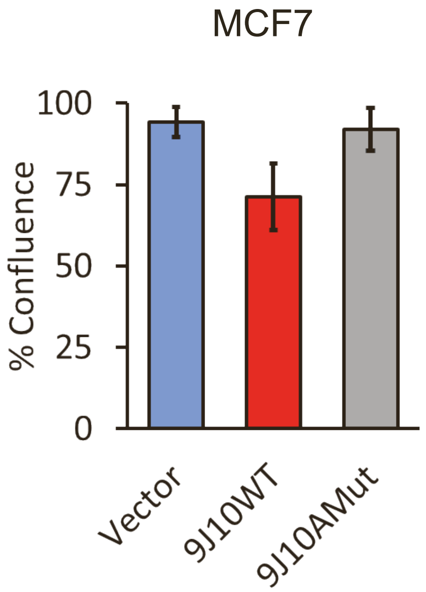

C

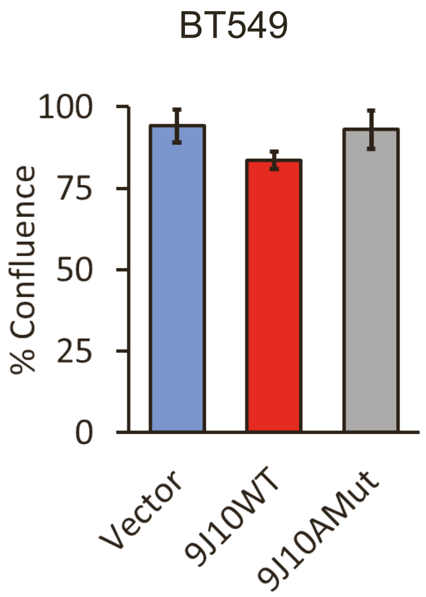

D

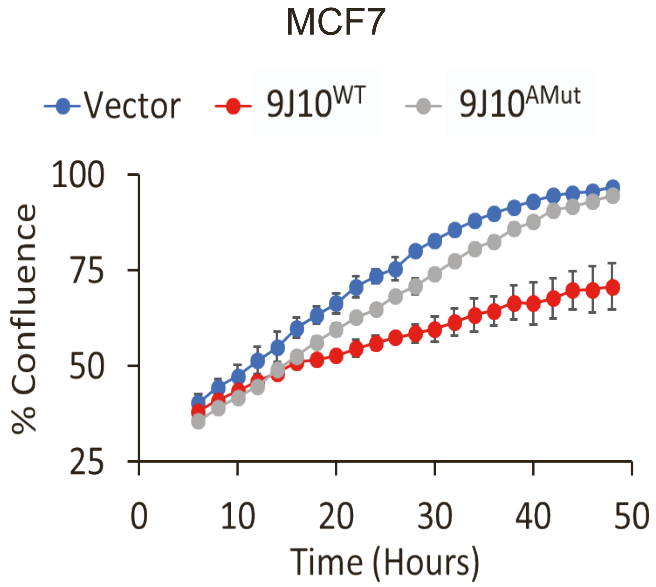

E

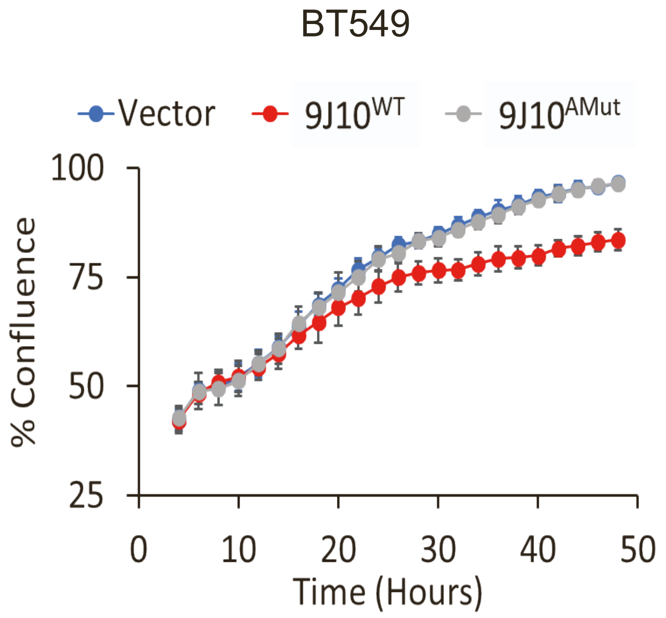

Supplementary Figure S5: Binding by ITC and co-crystal structure of 9J10 and 14-3-3ε.

A

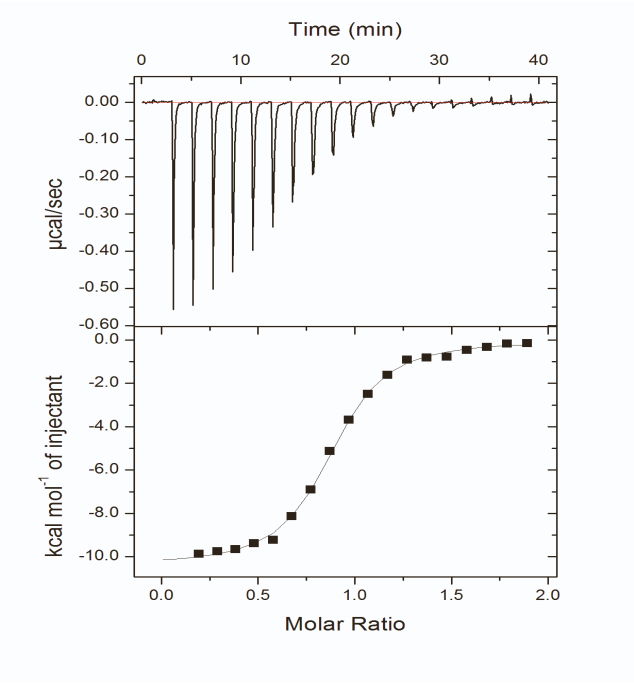

B

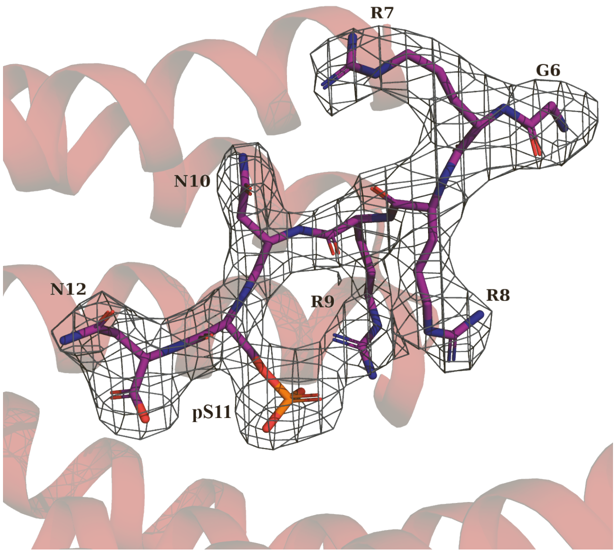

C

Interactions	14-3-3 (atom)	9J10 (atom)	Distance (Å)
H-Bonds	Asn 227 (OD1)	Asn 10 (N)	2.8
	Asn 227 (OD1)	Asn 10 (ND2)	3.3
	Asn 227 (ND2)	Asn 10 (O)	3.2
	Leu 223 (O)	Asn 10 (ND2)	3.1
	Asn 176 (OD1)	Asn 12 (N)	3.0
	Asn 176 (ND2)	Asn 12 (O)	3.0
	Arg 130 (NH2)	Sep 11 (O1P)	3.6
	Arg 130 (NH1)	Sep 11 (O2P)	2.7
	Arg 57 (NH1)	Sep 11 (O1P)	3.5
	Arg 57 (NH1)	Sep 11 (O3P)	3.3
	Arg 57 (NH2)	Sep 11 (O3P)	3.0
	Tyr 131 (OH)	Sep 11 (O1P)	2.6
Salt Bridges	Asp 226 (OD1)	Arg 7 (NH1)	3.4
	Asp 226 (OD1)	Arg 7 (NH2)	3.5
	Glu 183 (OE1)	Arg 9 (NE)	2.7
	Glu 183 (OE2)	Arg 9 (NE)	3.3
	Glu 183 (OE1)	Arg 9 (NH2)	3.6
	Glu 183 (OE2)	Arg 9 (NH2)	3.2
	Glu 134 (OE1)	Arg 9 (NH2)	3.4
	Lys 123 (NZ)	Asn 12 (O)	2.7

D

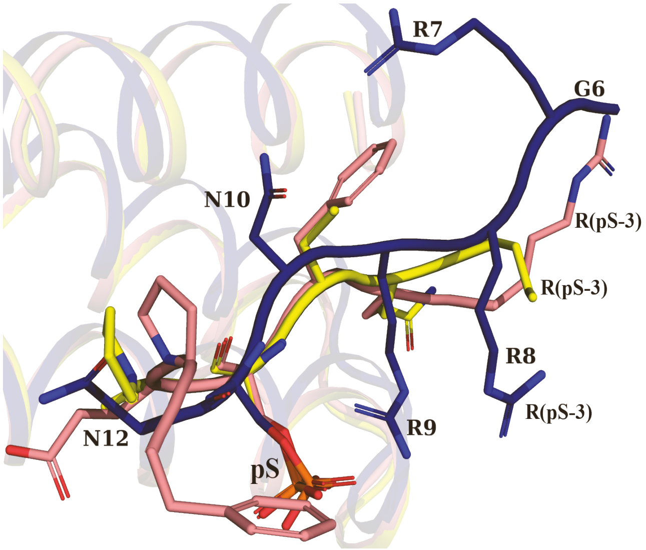

E

Pairs	Pair distance (CZ-CZ) d(Å)	Plane angle (NH1-NH2-NE) ϴ(°)
Arg61-Arg8	3.5	32.6
Arg8-Arg9	4.4	57.4

Supplementary Figure S6: Identification and validation of compound CU7218.

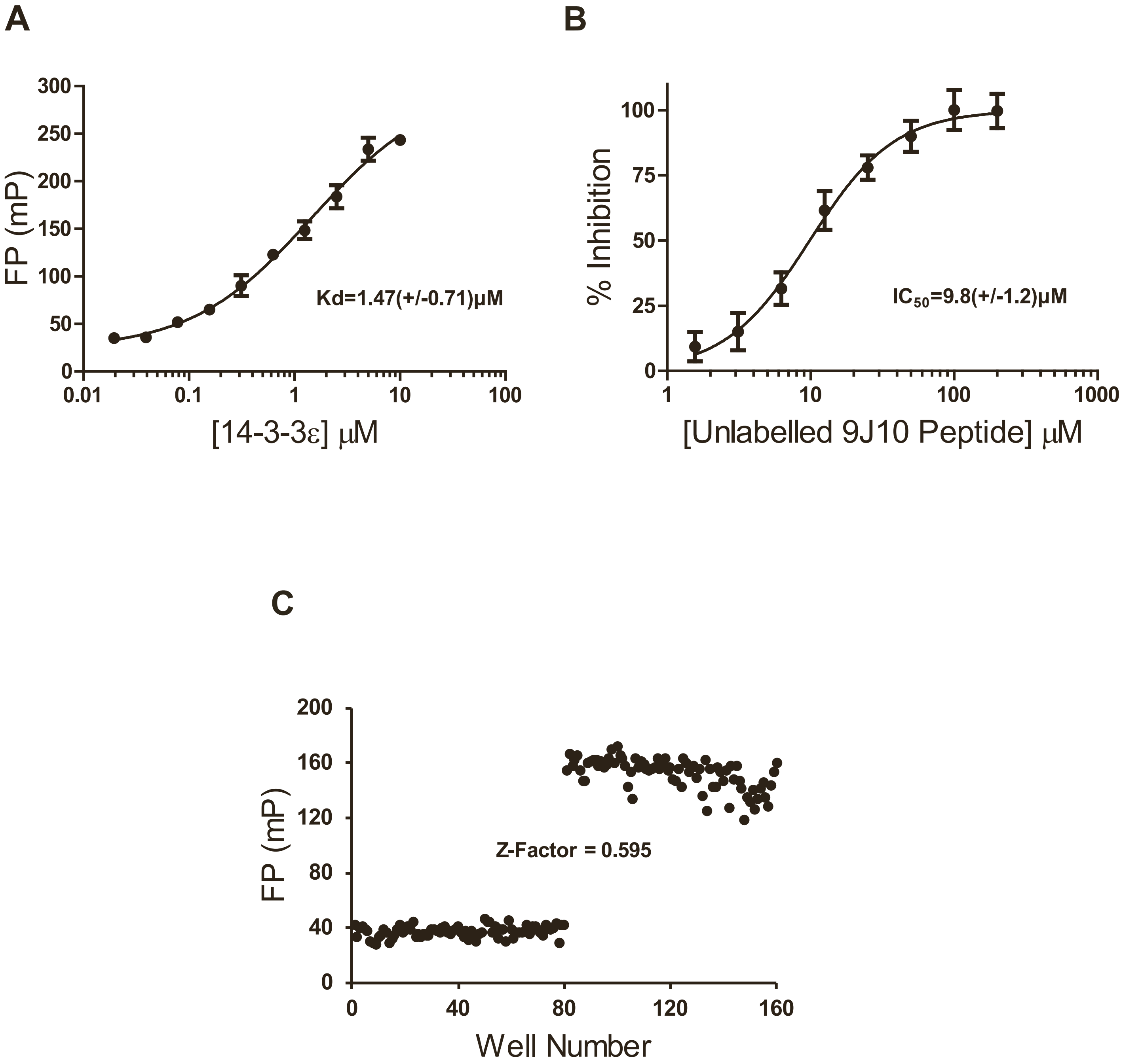

Supplementary Table S1: List of genomes used to generate the peptide library including genome length and percentage representation of each genome within the library (related to STAR Methods).

**Bacteria**	Genome length (bp)	Expected representation	Actual representation
Acinetobacter baumannii	4001457	3.21%	2.82%
Aeromonas hydrophila	4744448	3.81%	8.47%
Bacillus cereus	5432652	4.36%	2.80%
Bacteroides thetaiotaomicron	6293399	5.05%	5.20%
Bordetella pertussis	4086189	3.28%	5.39%
Borrelia burgdorferi	1397488	1.12%	1.65%
Campylobacter jejuni	1641481	1.32%	1.11%
Caulobacter crescentus	4016947	3.22%	4.00%
Chlorobium tepidum	2154946	1.73%	2.02%
Clostridium difficile	4298133	3.45%	2.06%
Corynebacterium diphtheriae	2488635	2.00%	1.36%
Deinococcus radiodurans	3284159	2.64%	2.17%
Desulfovibrio vulgaris	3773159	3.03%	2.82%
Geobacter sulfurreducens	3814128	3.06%	2.21%
Haemophilus influenzae	1830138	1.47%	0.81%
Helicobacter pylori	1667867	1.34%	1.13%
Legionella pneumophila	3397754	2.73%	1.61%
Listeria monocytogenes	2944528	2.36%	1.33%
Mycobacterium tuberculosis	4419977	3.55%	4.94%
Neisseria meningitidis	2194961	1.76%	1.52%
Porphyromonas gingivalis	2343476	1.88%	1.57%
Pseudomonas aeruginosa	6264404	5.03%	4.62%
Rhodobacter sphaeroides	4603060	3.69%	3.73%
Rhodopseudomonas palustris	5467640	4.39%	4.38%
Salmonella enterica	4951371	3.97%	2.43%
Streptomyces avermitilis	8482895	6.81%	5.87%
Thermotoga maritima	1860725	1.49%	1.22%
**Archaea**	Genome length (bp)	Expected representation	Actual representation
Archaeoglobus fulgidus	2178401	1.75%	1.55%
Aeropyrum pernix	1669696	1.34%	1.70%
Haloarcula marismortui	4274642	3.43%	3.69%
Halobacterium	2571010	2.06%	2.54%
Haloferax volcanii	4012900	3.22%	6.58%
Methanocaldococcus jannaschii	1739927	1.40%	0.83%
Pyrococcus horikoshii	1738505	1.40%	0.75%
Sulfolobus solfataricus	2992245	2.40%	1.70%
Thermoplasma volcanium	1584804	1.27%	1.44%

Supplementary Table S2: Validation of 59 primary hits in the FOXO3a Relocalisation Assay in U2OS-GFP-FOXO3a cells (related to Figure 1).

Peptide ID	Mean fold change relative to vector	Mean % cells with nuclear GFP-FOXO3a	Genome of origin
3F14	3.1 +/-1.3	4.6 +/-2.7	*Bordetella pertussis*
17C10	2.6 +/-0.8	4.1 +/-2.8	*Pseudomonas aeruginosa*
24J09	2.5 +/-0.9	3.8 +/-2.1	*Geobacter sulfurreducens*
11H05	2.4 +/-0.6	3.4 +/-1.1	*Rhodopseudomonas palustris*
2E21	2.3 +/-0.5	3.3 +/-0.7	*Aeromonas hydrophila and Legionella pneumophila*
1K05	2.2 +/-0.7	3.0 +/-0.3	*Streptomyces avermitilis*
1F13	2.2 +/-0.6	3.0 +/-0.4	*Bordetella pertussis*
12C03	2.1 +/-0.5	3.1 +/-1.4	*Streptomyces avermitilis*
12L09	2.1 +/-0.4	3.0 +/-0.8	*Bordetella pertussis*
22C04	2.1 +/-0.4	3.1 +/-1.1	*Aeromonas hydrophila*
1O23	2.0 +/-0.7	2.7 +/-0.3	*Unknown*
19I03	2.0 +/-0.6	2.9 +/-1.0	*Rhodobacter sphaeroides*
9J10	2.0 +/-0.3	2.9 +/-0.8	*Streptomyces avermitilis*
1M15	1.9 +/-0.2	3.0 +/-1.3	
20B17	1.9 +/-0.6	2.7 +/-0.7	
18C08	1.9 +/-0.4	2.8 +/-1.3	
14E14	1.9 +/-0.3	2.9 +/-1.4	
18D09	1.8 +/-0.4	2.6 +/-0.9	
18B15	1.8 +/-0.3	2.6 +/-1.2	
19B02	1.7 +/-0.6	2.3 +/-0.4	
18D07	1.7 +/-0.5	2.3 +/-0.2	
17J10	1.7 +/-0.3	2.5 +/-0.9	
11H13	1.6 +/-0.7	2.1 +/-0.2	
17I09	1.6 +/-0.7	2.1 +/-0.6	
17C16	1.6 +/-0.4	2.3 +/-0.5	
18B09	1.6 +/-0.1	2.5 +/-1.0	
3E10	1.5 +/-0.5	2.0 +/-0.2	
18B21	1.5 +/-0.4	2.1 +/-0.4	
7J15	1.5 +/-0.2	2.4 +/-1.2	
24J12	1.4 +/-0.5	1.9 +/-0.4	
11F11	1.4 +/-0.4	1.9 +/-0.4	
3L09	1.4 +/-0.2	2.0 +/-0.7	
12H13	1.3 +/-0.3	1.8 +/-0.4	
10J20	1.2 +/-0.5	1.7 +/-0.5	
19G09	1.0 +/-0.2	1.4 +/-0.4	
Vector	1.0 +/-0.0	1.5 +/-0.7	
10N04	0.9 +/-0.5	1.1 +/-0.2	
3I12	0.9 +/-0.3	1.2 +/-0.2	

10L18	0.9 +/-0.3	1.3 +/-0.2	
3H08	0.9 +/-0.2	1.2 +/-0.2	
5E23	0.9 +/-0.2	1.2 +/-0.3	
21D02	0.9 +/-0.1	1.3 +/-0.4	
3E14	0.8 +/-0.3	1.1 +/-0.2	
3H11	0.8 +/-0.3	1.1 +/-0.1	
8D06	0.8 +/-0.3	1.0 +/-0.2	
3H14	0.8 +/-0.2	1.1 +/-0.2	
6D21	0.8 +/-0.2	1.1 +/-0.3	
7J10	0.8 +/-0.2	1.1 +/-0.3	
11D11	0.8 +/-0.2	1.1 +/-0.2	
14N18	0.8 +/-0.2	1.2 +/-0.2	
8I08	0.8 +/-0.1	1.1 +/-0.4	
3B23	0.7 +/-0.3	0.9 +/-0.3	
5J03	0.7 +/-0.2	1.0 +/-0.2	
6N13	0.7 +/-0.2	1.0 +/-0.2	
7D15	0.7 +/-0.2	1.0 +/-0.3	
7F04	0.7 +/-0.2	1.0 +/-0.2	
8B23	0.7 +/-0.2	1.0 +/-0.2	
3F23	0.7 +/-0.1	1.0 +/-0.3	
10L04	0.6 +/-0.2	0.9 +/-0.1	
14F16	0.6 +/-0.2	0.9 +/-0.2	

Supplementary Table S4. Data collection and refinement statistics of 14-3-3ε:9J10 complex structure (related to Figure 5).

Parameters	14-3-3ε:9J10
Wavelength (Å)	1.54
Space group	$P2_12_12_1$
Unit cell parameters	
a,b,c (Å)	59.47, 83.35, 118.81
α, β, γ (°)	90 90 90
Resolution (Å)	59.47 – 3.16<br>(3.33 – 3.16)
R_{merge} (%)	14.5 (64.5)
Mean [<I>/σ(<I>)]	8.6 (2.3)
CC (1/2) (%)	99.5 (69.8)
Total no. of reflections	49,648 (6933)
Unique reflections	10, 688 (1516)
Completeness (%)	99.9 (99.5)
Multiplicity	4.6 (4.6)
Molecules in asymmetric unit	2
Solvent content (%)	39.56
**Refinement statistics**	
Resolution (Å)	30.00-3.16 (3.24-3.16)
Rwork (%)	22.17 (30.7)
Rfree (%)	22.05 (36.4)
Mean B-value (Å^2)	62.7
Ramachandran favoured (%)	95.9
Ramachandran outlier (%)	0
R.m.s. deviations	
Bond length (Å)	0.003
Bond angle (°)	1.241

Values in parentheses correspond to the highest resolution shell

Supplementary Table S5: List of siRNA target sequences (related to STAR Methods).

siRNA	Supplier	Target Sequence
RICTOR_1	Qiagen	ATGACCGATCTGGACCCATAA
RICTOR_3	Qiagen	TAGGTGCATTGACATACAACA
RICTOR_4	Qiagen	TACGAGCGCTTCGATATCTCA
RICTOR_5	Qiagen	TCCGATCATGGGCAGGTATTA
Non-Targeting Pool #2	Dharmacon	UAAGGCUAUGAAGAGAUAC,<br>AUGUAUUGGCCUGUAUUAG,<br>AUGAACGUGAAUUGCUCAA,<br>UGGUUUACAUGUCGACUAA
PDPK1 pool	Dharmacon	CAAGAGACCUCGUGGAGAA,<br>GACCAGAGGCCAAGAAUUU,<br>GGAAACGAGUAUCUUAUAU,<br>UGGCCAAAUUGCACGGAAU
PDPK1_5	Qiagen	AAGGGCATCATTCACAGGGAC
PDPK1_7	Qiagen	AACAAAGTTCTGAAAGGTGAA
PDPK1_9	Qiagen	CACGCCTAACAGGACGTATTA
XPO1 pool	Dharmacon	GAAAGUCUCUGUCAAAAUA,<br>GCAAUAGGCUCCAUUAGUG,<br>GGAACAUGAUCAACUUAUA,<br>GGAUACAGAUUCCAUAAAU
XPO1_1	Qiagen	CCCATTGTAAAGCGACTTCAA
XPO1_2	Qiagen	TACATGTTACTCCCTAATCAA
XPO1_5	Qiagen	TTCTCAGAATATGAATACGAA
XPO1_6	Qiagen	ATGGTTAGTCGAATGGCTAAA
