# Supplementary material for: Target identification for small-molecule discovery in the FOXO3a tumor-suppressor pathway using a biodiverse peptide library
Source: Cell Chem Biol. 2021 Nov 18;28(11):1602–1615.e9. doi: 10.1016/j.chembiol.2021.05.009 (PMC8610377; doi:10.1016/j.chembiol.2021.05.009)
Supplement: Document S2. Article plus supplemental information [file mmc4.pdf]

Article

Cell Chemical Biology

Target identification for small-molecule discovery in the FOXO3a tumor-suppressor pathway using a biodiverse peptide library

Graphical abstract

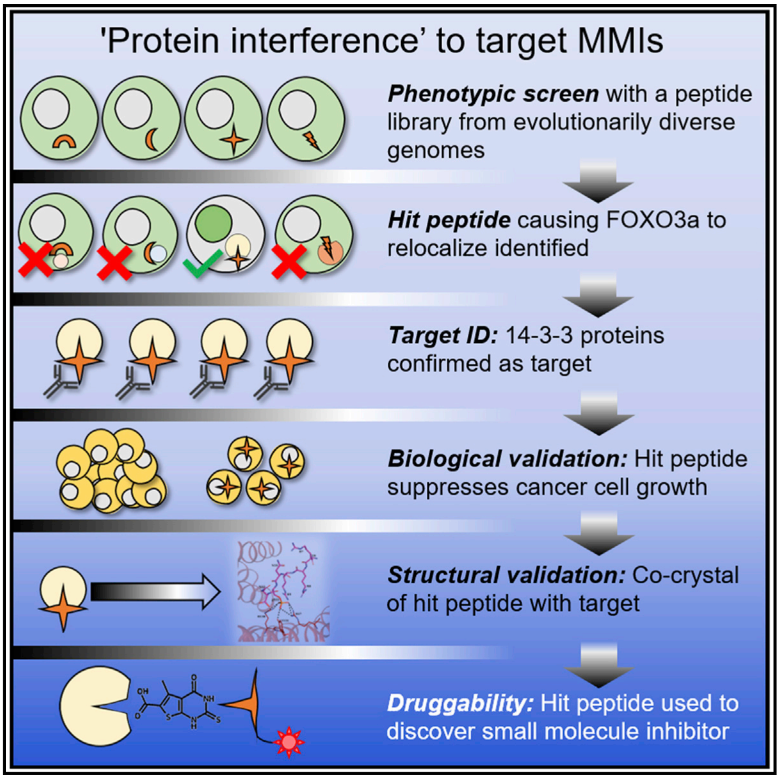

Highlights

- We describe protein interference, an approach to identify and validate new drug targets
- A genetic screen identifies a protein interference probe inducing FOXO3a reactivation
- The probe defines a druggable binding site in the 14-3-3 signal regulator family
- We illustrate a workflow to parse complex cellular pathways for new drug targets

Authors

Amy Emery, Bryn S. Hardwick, Alex T. Crooks, ..., Christopher J. Torrance, Grahame J. McKenzie, Ashok R. Venkitaraman

Correspondence

arv22@nus.edu.sg

In brief

Emery et al. describe “protein interference,” an approach to identify and validate new drug targets using structurally diverse natural peptides in genetic screens for cellular phenotypes. They discover a druggable site in 14-3-3 proteins that reactivates FOXO3a, and validate its structural features and biological activity.

Emery et al., 2021, Cell Chemical Biology *28*, 1602–1615
November 18, 2021 © 2021 The Author(s). Published by Elsevier Ltd.
https://doi.org/10.1016/j.chembiol.2021.05.009

CellPress

CellPress
OPEN ACCESS

Cell Chemical Biology

Article

Target identification for small-molecule discovery in the FOXO3a tumor-suppressor pathway using a biodiverse peptide library

Amy Emery,[1] Bryn S. Hardwick,[1] Alex T. Crooks,[1] Nadia Milech,[2] Paul M. Watt,[2] Chandan Mithra,[3] Vikrant Kumar,[3] Saranya Giridharan,[3] Gayathri Sadasivam,[3] Subashini Mathivanan,[3] Sneha Sudhakar,[3] Sneha Bairy,[3] Kavitha Bharatham,[3] Manjunath A. Hurakadli,[3] Thazhe K. Prasad,[3] Neelagandan Kamariah,[3] Markus Muellner,[4] Miguel Coelho,[4] Christopher J. Torrance,[4] Grahame J. McKenzie,[1,4] and Ashok R. Venkitaraman[1,3,4,5,6,*]
[1]Medical Research Council Cancer Unit, University of Cambridge, Hills Road, Cambridge CB2 0XZ, UK
[2]Telethon Kids Institute, Centre for Child Health Research, University of Western Australia & PYC Therapeutics Limited, Nedlands, WA 6009, Australia
[3]Center for Chemical Biology & Therapeutics, inStem & NCBS, Bellary Road, Bangalore 560065, India
[4]PhoreMost Ltd., Babraham Research Campus, Cambridge CB22 3AT, UK
[5]Present address: Cancer Science Institute of Singapore, National University of Singapore, Centre for Translational Medicine,14 Medical Drive #12-01, Singapore 117599 & Agency for Science, Technology and Research (A*STAR), 8A Biomedical Grove, Singapore 138648
[6]Lead contact
*Correspondence: arv22@nus.edu.sg
https://doi.org/10.1016/j.chembiol.2021.05.009

SUMMARY

Genetic screening technologies to identify and validate macromolecular interactions (MMIs) essential for complex pathways remain an important unmet need for systems biology and therapeutics development. Here, we use a library of peptides from diverse prokaryal genomes to screen MMIs promoting the nuclear relocalization of Forkhead Box O3 (FOXO3a), a tumor suppressor more frequently inactivated by post-translational modification than mutation. A hit peptide engages the 14-3-3 family of signal regulators through a phosphorylation-dependent interaction, modulates FOXO3a-mediated transcription, and suppresses cancer cell growth. In a crystal structure, the hit peptide occupies the phosphopeptide-binding groove of 14-3-3ε in a conformation distinct from its natural peptide substrates. A biophysical screen identifies drug-like small molecules that displace the hit peptide from 14-3-3ε, providing starting points for structure-guided development. Our findings exemplify "protein interference," an approach using evolutionarily diverse, natural peptides to rapidly identify, validate, and develop chemical probes against MMIs essential for complex cellular phenotypes.

INTRODUCTION

A complex network of macromolecular interactions (MMIs) involving proteins as well as other biological macromolecules mediate intracellular signaling. Computational methods predict 300,000 high-confidence binary MMIs involving human proteins (Zhang et al., 2012), but there may be many more. Parsing MMI networks to pinpoint rate-limiting interactions responsible for cellular phenotypes is challenging due to network complexity and redundancy but also the lack of appropriate tools (Hart et al., 2006; Kovacs et al., 2019; Luck et al., 2017). Libraries of short peptides, alone or mounted in scaffolds, have been screened using a range of methods for their ability to disrupt MMIs and induce phenotypes in yeast and bacteria (Blum et al., 2000; Caponigro et al., 1998; Norman et al., 1999). However, there is limited information concerning their use for genetic screens in mammalian cells to interrogate phenotypes beyond simple cell viability (Nim et al., 2016; Xu et al., 2001). Moreover, it is unclear whether libraries of biologically derived peptides may exhibit superior hit rates in complex phenotypic screens (Nim et al., 2016; Watt, 2006) compared with random or motif-based peptides (Xu et al., 2001). Finally, the attractive possibility that biologically derived peptides identified in such screens could serve as templates to discover small-molecule inhibitors of MMIs remains to be validated empirically.

To address these issues, we used a library of short (average, 31 residues) peptides encoded in diverse prokaryal genomes (Watt et al., 2017) to parse in human cells a well-characterized, therapeutically relevant pathway to identify and validate rate-limiting MMIs as potential targets for small-molecule inhibitors. We interrogated an intracellular signaling pathway frequently hyperactivated in human cancers and mediated by the enzymes phosphatidylinositol 3-kinase (PI3K) and protein kinase B (AKT) (Fresno Vara et al., 2004; Vivanco and Sawyers, 2002). Its constitutive activation inactivates the tumor-suppressive transcription factor, FOXO3a (Brunet et al., 1999; Burgering and Kops, 2002), by inducing its phosphorylation and cytosolic sequestration by factors such as the 14-3-3 protein family (Brunet et al., 2002; Mackintosh, 2004; Van Der Heide et al., 2004). Cytosolic FOXO3a sequestration and inactivation has been implicated in

 © 2021 The Author(s). Published by Elsevier Ltd.
This is an open access article under the CC BY license (http://creativecommons.org/licenses/by/4.0/).

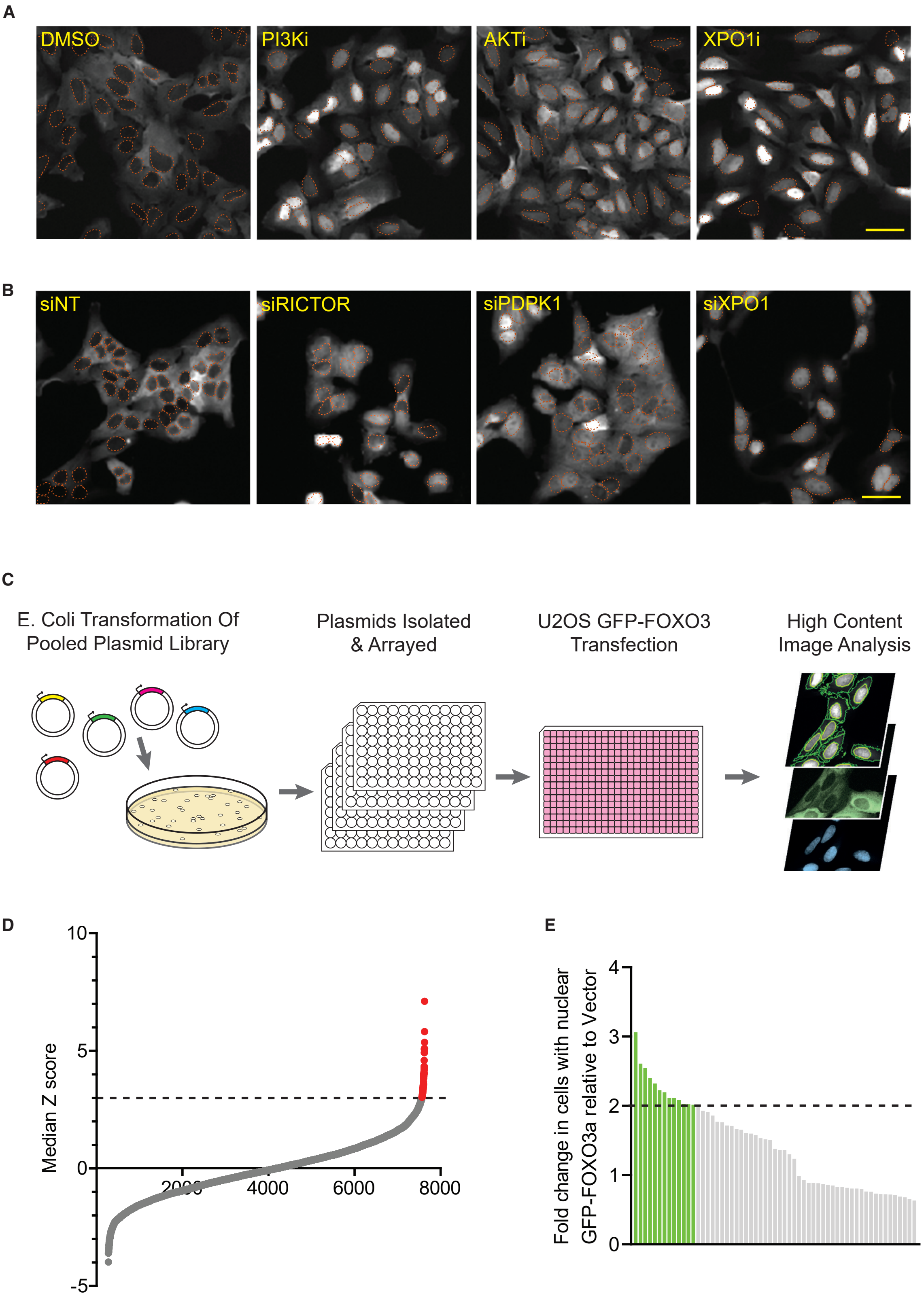

(legend on next page)

tumorigenesis (Dansen and Burgering, 2008; Hu et al., 2004; Liu et al., 2018; Yang et al., 2008) and signifies poor prognosis in a wide range of human tumors (Ni et al., 2014; Santo et al., 2013; Xie et al., 2012; Yang et al., 2013). Conversely, restoration of FOXO3a nuclear localization by genetic (Hu et al., 2004; Reagan-Shaw and Ahmad, 2007; Zou et al., 2008) or pharmacologic (Dong et al., 2007; Santo et al., 2013; Yang et al., 2010) means suppresses cancer cell growth. Because FOXO3a is typically inactivated in human cancers by phosphorylation rather than mutation, modulation of this pathway has attracted attention as a potential anti-cancer therapeutic strategy (Farhan et al., 2017; Liu et al., 2018; Yang and Hung, 2009; Zanella and Carnero, 2009).

Here, we report findings that illustrate the potential of biodiverse peptides to interfere with MMIs that inactivate FOXO3a in human cells, enabling an innovative approach for target validation and small-molecule inhibitor discovery. We demonstrate that one such peptide engages 14-3-3 proteins, and interferes with FOXO3a suppression, via a phosphorylation-dependent interaction. Expression of the peptide in cells modulates a broad transcriptional response, including FOXO-dependent transcripts, and causes growth suppression in cancer cell lines, speaking to its biological activity. X-ray crystallography reveals a non-canonical structural interaction between the peptide and 14-3-3ε. A high-throughput biophysical screen identifies drug-like small molecules capable of inhibiting the peptide/14-3-3ε interaction. Thus, collectively, our results provide a blueprint for the use of biodiverse peptide libraries in genetic screens to identify and validate potentially druggable MMIs that mediate complex phenotypes in human cells.

RESULTS

A DNA fragment library encoding biodiverse peptides from prokaryal genomes

We generated a novel library of DNA fragments from a mega-genome comprising 27 bacterial and 9 archaeal genomes, chosen for their phylogenetic diversity (see STAR methods and Table S1). In brief, peptide-encoding DNA fragments were amplified and cloned into a mammalian expression vector, in-frame with a V5 epitope tag sequence (Southern et al., 1991) positioned 5′ to the insert, to enable identification of interacting proteins by co-immunoprecipitation. Based on the Clarke-Carbon formula (Clarke and Carbon, 1976), which calculates the probability of any DNA sequence being included in a library of random fragments, the library complexity of 1.2×10^8 colony-forming units suggests a 1.7-fold coverage of the mega-genome. Next-generation sequencing of the library indicates an average insert length of 210 base pairs which, upon further analysis, corresponded to an actual mean length of 31 residues for the encoded peptides due to the presence of in-frame stop codons within some of the DNA sequences.

Cell-based phenotypic screen for nuclear FOXO3a relocalization

Phenotypic screens were performed in the human osteosarcoma cell line U2OS, which recapitulates the PI3K/AKT-mediated inactivation of FOXO3a by cystosolic sequestration (Brunet et al., 2002; Cautain et al., 2016; Link et al., 2009; Zanella et al., 2008). We developed a U2OS reporter cell line that stably expresses GFP-FOXO3a (referred to as U2OS-GFP-FOXO3a) to readily monitor intracellular localization of FOXO3a. In these cells, GFP-FOXO3a is predominantly localized to the cytosol and can be relocalized to the nucleus upon addition of PI3K or AKT inhibitors, or by treatment with leptomycin B, an inhibitor of the nuclear export protein Exportin 1 (XPO1) (Figures 1A and S1A). Moreover, depletion of key factors in the AKT and nuclear export pathways using small interfering RNA (siRNA) induced an increase in nuclear localization of GFP-FOXO3a (Figures 1B and S1B). Thus, these results demonstrate that localization of the GFP-FOXO3a protein responds to perturbations in the AKT and nuclear export pathways and validate the use of U2OS-GFP-FOXO3a cells for assay development.

We developed a high-content screening assay to quantitate the nuclear localization of GFP-FOXO3a. The intensity of GFP-FOXO3a was measured in both nuclear and cytoplasmic compartments of each cell using automated microscopic imaging. A nuclear/cytoplasmic ratio was calculated for each cell, and this was used to classify each cell as having either nuclear or cytoplasmic GFP-FOXO3a (i.e., having a nuclear/cytoplasmic ratio above or below a user-defined value). The threshold was determined in validation experiments using the PI3K inhibitor, PI-103, or dimethyl sulfoxide (DMSO) vehicle alone (see STAR methods). This enabled results to be expressed as the percentage of cells with nuclear GFP-FOXO3a (Figures S1A and S1B).

We carried out a screen for nuclear FOXO3a relocalization using a plasmid-encoded mini-library of ∼7,000 peptides (Table S1) randomly sampled from the full library comprising $\sim 10^8$ elements. The U2OS-GFP-FOXO3a reporter cell line was transfected with the arrayed peptide library in 384-well format, and GFP-FOXO3a localization was measured by high-content screening as described above (Figure 1C). The percentage of cells with nuclear GFP-FOXO3a was calculated as described in STAR methods) through automated image analysis. The screen yielded 364 hits with a Z score ≥ 2 and 59 hits with a Z score ≥ 3 (Figure 1D). These 59 peptides were selected for validation, and 13 (a hit rate of ∼0.2%) were shown to reproducibly induce at least a 2-fold increase in the percentage of cells with nuclear FOXO3a (Figure 1E and Table S2).

Figure 1. High-content screening of a peptide library to identify FOXO3a nuclear translocation in a U2OS reporter cell line

(A) Representative images of U2OS-GFP-FOXO3a cells showing GFP-tagged FOXO3a localization after treatment with small-molecule inhibitors or (B) transfection with siRNAs targeting the AKT and nuclear export pathways. PI3Ki, 1 μM PI-103; AKTi, 1 μM GSK690693; XPO1i, 2 nM leptomycin. The nuclear area is marked by an orange dotted line. Scale bars, 50 μm. See Figure S1 for quantitation.

(C) Schematic of screening workflow.

(D) Scatterplot of primary screen results showing a ranked distribution of peptides according to the robust Z score. Red dots indicate a Z score greater than 3.

(E) Bar graph showing validation of 59 selected primary hits, ranked by fold change in percentage of cells with nuclear FOXO3a relative to vector. Those over 2-fold were selected for further work.

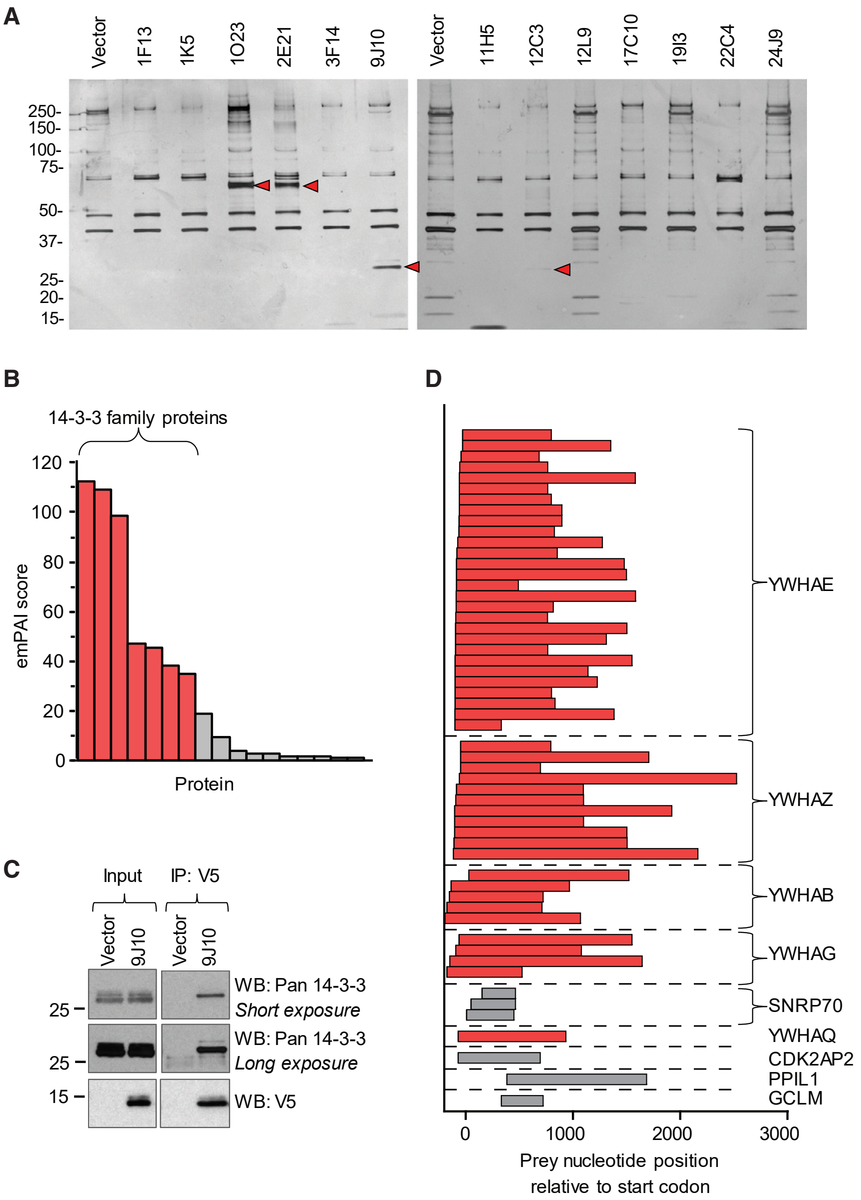

Figure 2. Peptide 9J10 interacts with 14-3-3

(A) Silver-stained gels of eluted proteins following immunoprecipitation with V5 antibody from U2OS-GFP-FOXO3a cells transfected with the indicated peptide for 48 h. Red arrowheads indicate differential bands (relative to vector), which were excised for protein identification by mass spectrometry.

(B) Graph showing proteins identified by mass spectrometry for the band from 9J10, ranked by emPAI score. Members of the 14-3-3 family are highlighted in red. Proteins with an emPAI >1 are displayed; see Table S3 for complete list.

(C) Western blotting for 14-3-3 following immunoprecipitation (IP) with V5 antibody from U2OS-GFP-FOXO3a cells transfected with 9J10 or vector for 48 h. A representative blot from three independent replicates is shown.

(D) Graphical representation of yeast two-hybrid experiment using 9J10 as bait, showing prey fragments for interacting proteins. Members of the 14-3-3 family are highlighted in red.

Characterization of peptides that modulate FOXO3a relocalization

Primary target identification was carried out by immunoprecipitation of the peptides from U2OS-GFP-FOXO3a cell lysates using the V5 epitope tag. Eluted proteins were separated by SDS-PAGE and visualized using silver staining to enable identification of co-immunoprecipitating proteins. Four hit peptides, designated 1O23, 2E21, 9J10, and 12C3 (lanes 4, 5, 7, and 10 in Figure 2A), showed clear differential bands (i.e., bands present in the peptide sample but absent in the vector control). Differential bands were excised and analyzed by liquid chromatography in combination with tandem mass spectrometry (Figure 2A).

One hit peptide, 9J10, was found to pull down a protein band with an apparent molecular weight between 25 and 37 kDa. Mass spectrometry revealed that the most abundant proteins in this band were the seven isoforms of the 14-3-3 family of proteins (Figure 2B and Table S3) whose predicted molecular weight ranges from 27.8 to 29.3 kDa. The exponentially modified protein abundance index (emPAI) (Ishihama et al., 2005) was used as a measure of abundance.

The 9J10/14-3-3 interaction was validated by western blotting for 14-3-3 following co-immunoprecipitation with 9J10 from lysates using anti-V5 antibody (Figures 2C and S2A). The interaction persisted under higher-stringency wash conditions, speaking to its high binding affinity (Figure S2B). While 14-3-3 family members were also identified in the band for 12C3 (Table S3), this interaction was not validated by western blotting (Figures S2A and S2B) and was therefore not pursued.

To further validate the interaction of 9J10 with 14-3-3 proteins, we used a yeast two-hybrid screen as an orthogonal means for target identification. The hit peptide, 9J10, was used as bait to screen a cDNA library from the HeLa human cell line. Several members of the 14-3-3 family were confirmed as interactors (Figure 2D). Thus, the interaction of 9J10 with 14-3-3 proteins was confirmed using two independent approaches.

Sequencing of 9J10 reveals that it encodes a 95-amino-acid peptide derived from a currently unannotated region of the *Streptomyces avermitilis* genome (Figure S2C). This soil bacterium contains the largest genome of those species included in the peptide library (Table S1). While the 9J10 peptide is notably larger than the average peptide size for the library (31 amino acids), the 13 hit peptides analyzed in Figure 2A (varying from 5 to 158 amino acids) have an average size of 61 amino acids (and a median of 67 amino acids), suggesting an enrichment for longer peptides in our screen.

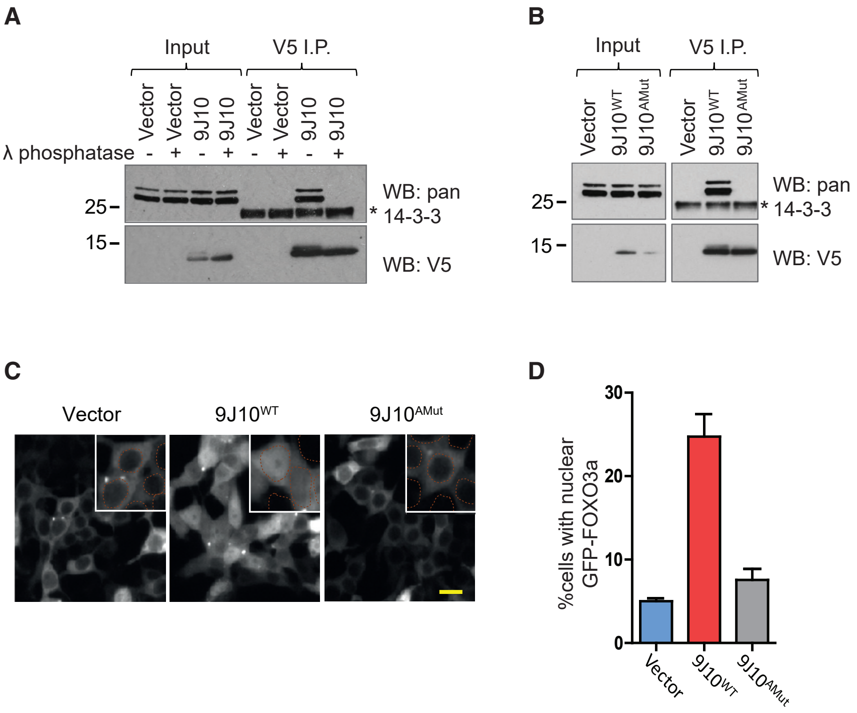

Figure 3. The interaction between 9J10 and 14-3-3 is phospho-dependent

(A) Western blotting for 14-3-3 following immunoprecipitation with V5 antibody from HEK293T cells transfected with 9J10 or vector for 24 h. Lysates were incubated in the presence or absence of lambda phosphatase.

(B) Western blotting for 14-3-3 following immunoprecipitation (I.P.) with V5 antibody from HEK293T cells transfected for 24 h with vector, wild-type 9J10 ($9J10^{WT}$), or 9J10 Ser94>Ala mutant ($9J10^{AMut}$). The asterisk marks the antibody light chain. All western blots are representative of at least two independent experiments.

(C) Representative images of HEK293T cells co-transfected with GFP-FOXO3a and vector, $9J10^{WT}$ or $9J10^{AMut}$, fixed at 24 h post transfection. GFP-FOXO3a localization is shown. Inset shows an enlarged region with the nucleus marked by an orange dotted line. Scale bar, 25 μm.

(D) Quantitation of FOXO3a localization from cells treated as in (C). Data represent the mean of three independent experiments ± SD.

Notably, the interaction between 9J10 and 14-3-3 proteins could be confirmed in a panel of human cell lines (Figure S2D), in which the FOXO3a tumor-suppressive pathway is inactivated. Thus, the 14-3-3 proteins were co-immunoprecipitated with 9J10 in lysates from the breast cancer cell line MCF7 and the ovarian cancer cell line SKOV3 (both of which carry activating mutations in PI3K), the breast cancer cell line BT-549 (which carries a frameshift mutation inactivating PTEN), and the transformed human embryonic kidney cell line HEK293T. We find that FOXO3a is predominantly cytoplasmic in HEK293T cells and translocates to the nucleus after exposure to small-molecule inhibitors of PI3K or AKT (Figure S3), recapitulating findings in the U2OS-GFP-FOXO3a reporter cell line and confirming FOXO3a suppression via PI3K/AKT activity. Collectively, these observations confirm that 9J10 interacts with 14-3-3 proteins in several cancer cell lines wherein FOXO3a is suppressed by different mechanisms.

Phosphorylated 9J10 engages 14-3-3 proteins

Homodimers of 14-3-3 proteins, or heterodimers with other 14-3-3 isoforms (Chaudhri et al., 2003), bind a wide range of cellular substrates and often sequester them in the cytoplasm (Mackintosh, 2004; Pozuelo Rubio et al., 2004; Tinti et al., 2014). Most 14-3-3 substrates are phosphorylated proteins in which a consensus phosphopeptide motif is recognized by a structurally characterized binding pocket formed by 14-3-3 multimers (Mackintosh, 2004; Yang et al., 2006). Interestingly, algorithmic prediction (http://www.compbio.dundee.ac.uk/1433pred) (Madeira et al., 2015) reveals a potential 14-3-3 binding motif at the C terminus of 9J10. This 9J10 motif, 91-Arg-Arg-Asn-Ser-Asn-95 (RRNSN, Figure S2C), is similar to the consensus 14-3-3 binding motif RXX-pS/T-XP (Muslin et al., 1996; Rittinger et al., 1999; Yaffe et al., 1997). This observation suggests that the 9J10/14-3-3 interaction may be mediated by phosphorylation of the Ser residue in the motif RRNSN.

Indeed, our results provide multiple lines of evidence supporting this conclusion. First, the interaction between 9J10 and 14-3-3 in cell extracts was inhibited by treatment with λ-phosphatase (Figure 3A). Notably, phosphatase treatment also depleted a slower-migrating form of 9J10, which may represent a phosphorylated form of the peptide. Second, mutation of Ser94 in 9J10 to a non-phosphorylatable Ala residue abolishes the co-immunoprecipitation of 14-3-3 proteins with 9J10 (Figure 3B). Third, the same mutant form of 9J10 also fails to induce the nuclear localization of GFP-FOXO3a (Figures 3C and 3D). Collectively, these results provide compelling evidence that 9J10 phosphorylation on Ser94 confers its biochemical and biological activity and illustrates how phenotypic screening using biodiverse peptides may identify macromolecular interactions mediated by phosphorylation.

9J10 modulates FOXO3a-dependent transcription

Next, we tested the ability of FLAG-tagged 14-3-3ε to co-immunoprecipitate with wild-type 9J10 ($9J10^{WT}$) using, as a control, the Ser94>Ala mutant ($9J10^{AMut}$), which does not interact with 14-3-3. Indeed, FLAG-14-3-3 co-immunoprecipitates with $9J10^{WT}$ but not $9J10^{AMut}$ (Figure 4A). Significantly, there is a sharp reduction in the interaction between FLAG-14-3-3 and FOXO3a in cells expressing $9J10^{WT}$, whereas $9J10^{AMut}$ fails to perturb this interaction (Figure 4A). 14-3-3ε promiscuously forms heterodimers with several other 14-3-3 isoforms, including 14-3-3γ (Chaudhri et al., 2003). Interestingly, neither $9J10^{WT}$ nor $9J10^{AMut}$ affect the ability of 14-3-3ε to heterodimerize with 14-3-3γ, suggesting that 9J10 engages the substrate recognition groove but not the dimerization interface.

Notably, $9J10^{WT}$ but not $9J10^{AMut}$ modulated FOXO3a-dependent gene transcription. Consistent with the release of FOXO3a from 14-3-3 in HEK293T cells (Figure 4A), we observed an

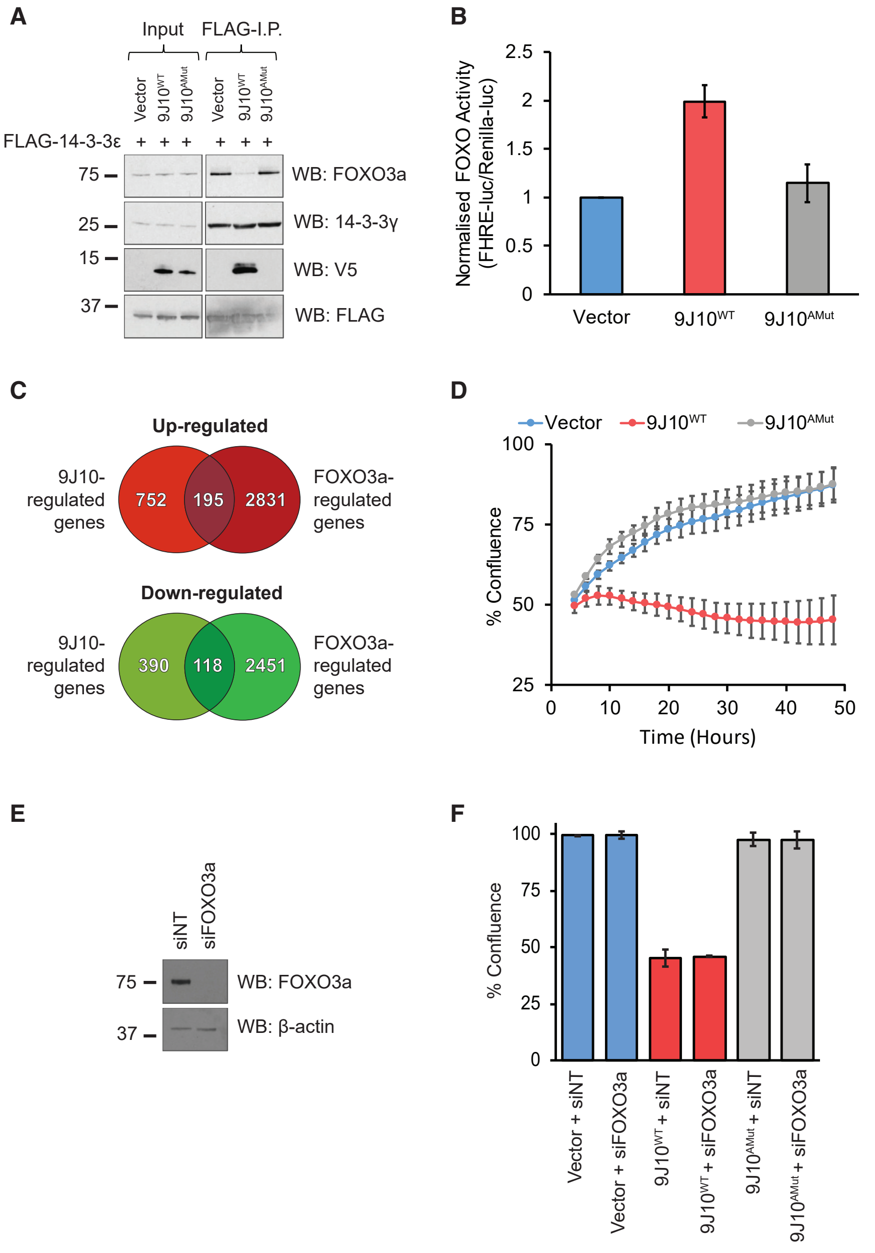

Figure 4. Expression of 9J10 reduces the interaction between 14-3-3 and FOXO3a, modulates transcription, and impairs cell growth in HEK293T cells

(A) Western blotting for FOXO3a and 14-3-3γ following immunoprecipitation (I.P.) with FLAG antibody from HEK293T cells co-transfected with FLAG-14-3-3ε and vector, $9J10^{WT}$, or $9J10^{Amut}$ for 24 h. All western blots are representative of at least two independent experiments.

(B) Normalized FHRE (Forkhead Response Element)-luciferase reporter activity in HEK293T transfected with vector, $9J10^{WT}$, or $9J10^{AMut}$ for 48 h. Data represent the mean of three independent experiments ± SD.

(C) Venn diagrams showing numbers of differentially expressed genes in response to 9J10 or FOXO3a-AAA relative to respective controls.

(D) Cell confluency measurements from live-cell imaging of HEK293T cells transfected with Vector, $9J10^{WT}$ or $9J10^{AMut}$ for 48 h. A representative experiment from three independent experiments is shown, data represent the average of four fields ± SD.

(E) Western blotting for expression of FOXO3a in cells transfected with vector in the presence of siNT or siFOXO3a. Samples were harvested at 48 h after plasmid transfection (72 h after siRNA transfection). β-Actin is included as a control for total protein levels. A representative example from three independent experiments is shown.

(F) Cell confluency measurements from live-cell imaging of HEK293T cells transfected with vector, $9J10^{WT}$, or $9J10^{AMut}$ in the presence of a non-targeting (siNT) or FOXO3a (siFOXO3a) siRNA at 48 h after plasmid transfection (72 h after siRNA transfection). Bars represent the mean of three independent experiments ± SD.

increase in transcriptional activity using a Forkhead response element luciferase reporter (Figure 4B). Moreover, transcriptome analysis using next-generation sequencing shows that transient $9J10^{WT}$ expression for 24 h induces a broad transcriptional response, which includes many known FOXO3a target genes, in common with expression of a constitutively active GFP-tagged FOXO3a mutant (FOXO3a-AAA) (Ramaswamy et al., 2002) (Figure 4C).

Because FOXO3a is reported to regulate different genes in different tissues and cell lines (Gross et al., 2008; Webb et al., 2016), we used a series of carefully controlled comparisons to analyze the effects of 9J10 expression. Thus, overexpression of constitutively active GFP-FOXO3a-AAA for 24 h caused upregulation of 3,026 genes and downregulation of 2,569 genes relative to the expression of GFP alone as a control (Figure 4C and Data S1). Of these, 195 upregulated genes and 118 downregulated genes were also differentially expressed in response to $9J10^{WT}$ relative to $9J10^{AMut}$, but not in cells overexpressing $9J10^{AMut}$ relative to empty vector. Taken together, these findings suggest that the transcriptional program regulated by $9J10^{WT}$ expression overlaps with that induced by expression of a constitutively active FOXO3a-AAA mutant.

Genes upregulated by both GFP-FOXO3a-AAA and $9J10^{WT}$ include the cyclin-dependent kinase inhibitors CDKN1A ($p21^{Cip1}$) (Seoane et al., 2004) and CDKN1B ($p27^{Kip1}$) (Accili and Arden, 2004), which are known FOXO3a targets. In addition, we analyzed transcription factor motif enrichment in the promoters of the 195 genes upregulated by both $9J10^{WT}$ and FOXO3a-AAA using oPOSSUM-3 (http://opossum.cisreg.ca/oPOSSUM3/), a web tool that detects over-representation of conserved transcription factor binding sites within a given gene set (Ho Sui et al., 2007; Kwon et al., 2012). oPOSSUM-3 analysis revealed that the most significantly enriched transcription factor binding motif in these 195 shared upregulated genes was indeed

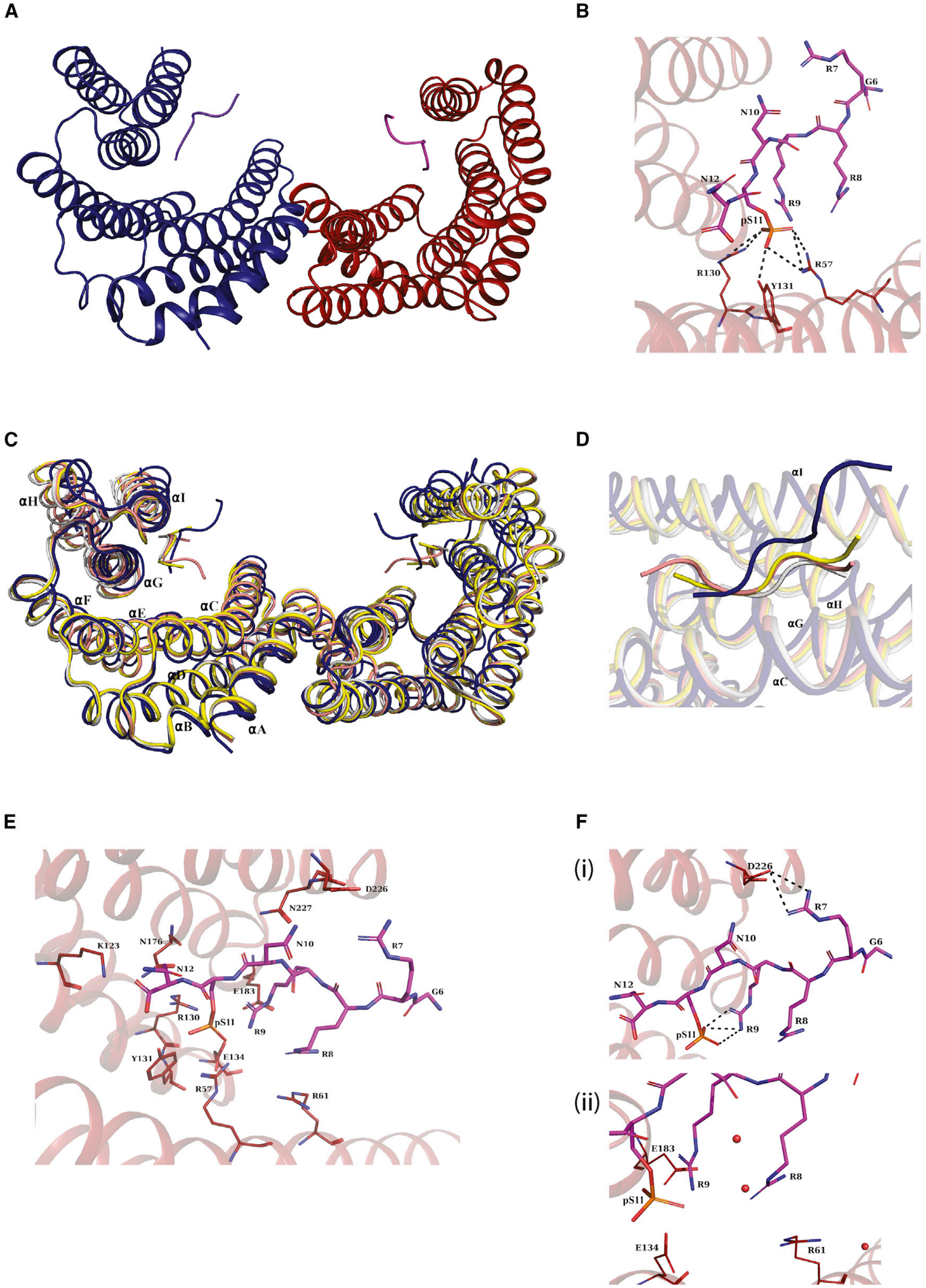

Figure 5. Co-crystal structure of 9J10 peptide and 14-3-3ε

(A) Ribbon representation of the 14-3-3ε dimer with each monomer (blue and firebrick) binding a single 9J10 phosphopeptide (purple and magenta).

(B) The phosphate moiety of 9J10 (magenta) anchors on the conserved Tyr131, Arg130, and Arg57 residues of 14-3-3ε.

(C) Structural variation of 14-3-3:9J10 complex. Comparison of dimeric 14-3-3ε:9J10 (blue) complex structure with other canonical phosphopeptide-bound structures of 14-3-3ε (PDB: 2BR9 [yellow]; PDB: 3UBW [orange]; PDB: 6EIH [gray]) show significant structural deviation in the C terminus.

(legend continued on next page)

FOXO3a. These findings suggest that 9J10^{WT} activates multiple FOXO3a target genes.

9J10 modulates a broad transcriptional program

Notably, however, the transcriptional program induced by 9J10^{WT} expression also includes 752 upregulated genes and 390 downregulated genes that were not regulated by FOXO3a-AAA (Figure 4C). oPOSSUM analysis reveals that in the gene set uniquely regulated by 9J10^{WT} (1,142 genes), 12 transcription factors showed significant motif enrichment (Figure S4A). Among these are HOXA5, NFYA, and EWSR1, which have been shown to co-immunoprecipitate with 14-3-3 in a closely related cell line (Laflamme et al., 2017). Thus, these findings suggest that 9J10 regulates a broad transcriptional program, beyond FOXO3a target genes alone, which includes the targets of other transcription factors known to bind 14-3-3.

9J10 suppresses cancer cell growth independent of FOXO3a

Next, we assessed the impact of these wide-ranging transcriptional changes on the growth of cancer cell lines. Notably, the expression of 9J10^{WT} but not 9J10^{AMut} suppressed cell growth in HEK293T cells (Figure 4D). A similar growth defect was also observed in the breast cancer cell lines MCF7 and BT-549 (c S4B and S4C), both of which carry activating mutations in the PI3K/AKT pathway. In no case did expression of 9J10^{AMut} suppress cell growth (Figures 4D, S4D, and S4E). However, efficient siRNA-mediated knockdown of FOXO3a (Figure 4E) failed to reverse the growth defect caused by 9J10^{WT} expression (Figure 4F) in HEK293T cells. We interpret these results to suggest that the biological activity of 9J10 in suppressing cancer cell growth may be independent of FOXO3a but instead requires elements of a broad transcriptional program mediated by multiple transcription factors, at least some of which are known to bind to the 14-3-3 family of signal regulators. Further dissection of the underlying mechanism warrants future studies.

Structure of a 9J10 phosphopeptide/14-3-3ε complex

We carried out X-ray crystallography of 14-3-3ε bound to a minimized 12-residue 9J10 peptide including the phospho-Ser residue (Figure 5 and Table S4). Its direct binding with 14-3-3ε was first confirmed by isothermal titration calorimetry (ITC), which demonstrated binding with a dissociation constant (K_D) of 0.70 ± 0.16 μM (Figure S5A). Subsequently, the co-crystal structure was solved at 3.16-Å resolution, with interpretable electron density for residues 2–232 of 14-3-3ε and seven peptide residues (6-GRRRNpSN-12, corresponding to residues 89–95 of the full-length 9J10 peptide) of 9J10 (Figure S5B). Within each 14-3-3ε dimer, a single 9J10 peptide interacts with each monomer at the substrate-binding groove (Figure 5A). The phosphate moiety from 9J10 pSer 94 occupies the canonical phosphate-binding pocket of 14-3-3ε, interacting with the conserved Arg57, Arg130, and Tyr131 residues (Figure 5B). Thus, our structural analyses are consistent with our biochemical findings and provide a structural mechanism for the interaction of 9J10 with the phosphopeptide-binding pocket of 14-3-3 dimers.

Interestingly, comparison with other available crystal structures of phosphopeptide-bound 14-3-3ε revealed several structural differences. The binding of 9J10 to 14-3-3ε was accompanied by the movement of the C-terminal helices αG, αH, and αI closer to the 9J10 peptide-binding groove by 2.2, 7.0, and 6.0 Å, respectively, causing 14-3-3ε to adopt a relatively "closed" conformation when compared with other structures (Figure 5C) (Borisova et al., 2018; Molzan et al., 2012; Yang et al., 2006). Moreover, the manner of 9J10 engagement with the substrate-binding groove of 14-3-3ε was distinct from that of other canonical phosphopeptides. While other canonical phosphopeptides (with the consensus sequence RXX-pS) bind in an extended conformation, 9J10 adopted a distinct, bent conformation, with the residue preceding the phospho-Ser turning upward toward the C-terminal αI helix (Figure 5D). Residues 9J10^{Arg7}, 9J10^{Arg9}, 9J10^{Asn10}, 9J10^{pSer11}, and 9J10^{Asn12} all make favorable interactions with 14-3-3ε (Figures 5E and S5C).

The atypical binding mode for 9J10 is characterized by the unique interactions of the three consecutive arginine residues in the 9J10 peptide. Of note are the intramolecular interactions between the phosphate moiety and the NH-guanidino group of 9J10^{Arg9} at the −2 position relative to pSer (i.e., pS(−2)), and the salt bridge between 9J10^{Arg7} at pS(−4) and 14-3-3ε^{Asp226} (Figure 5Fi). In addition, 9J10^{Arg8} at pS(−3) undergoes significant conformational change compared with the Arg at pS(−3) in the other canonical peptides (Figure S5D). The 14-3-3ε/9J10 complex is stabilized by Arg pairs via stacking of Arg residues 9J10^{Arg8} at pS(−3), 9J10^{Arg9} at pS(−2), and 14-3-3ε^{Arg61} (Figure S5E), while the phosphate moiety, 14-3-3ε^{Glu183}, 14-3-3ε^{Glu134}, and water molecules surrounding the Arg pairs play an important role in stabilizing the like-charge arginine pairing (Figure 5Fii).

Thus, our observations suggest that the phosphorylated 9J10 peptide engages 14-3-3ε through a structural mechanism distinct from that of consensus human phosphopeptide substrates and induces conformational changes that may influence substrate engagement. These differences highlight the ability of biodiverse peptides encoded by ancient genomes to engage novel, functionally relevant binding sites within human proteins, with implications for both target and inhibitor discovery.

Identification of drug-like small molecules that inhibit the 9J10/14-3-3 interaction

Next, we configured a biophysical screening assay to identify drug-like small molecules that inhibit the 9J10/14-3-3 interaction (Figure S6). The 9J10 phosphopeptide was minimized to the

(D) 9J10 peptide (dark blue) backbone adopts a bent conformation in the peptide-binding groove of 14-3-3ε when compared with other 14-3-3ε/peptide complexes (colored as in C).

(E) Unique interaction in the 14-3-3:9J10 complex. The residues involved in polar contacts between 14-3-3ε (firebrick sticks) and 9J10 peptide residues (magenta sticks) are shown.

(F) The unique interactions involving Arg9 at pS-2 and Arg7 at pS-4 are highlighted (i). Arginine pairings are formed by Arg8 and Arg9 of 9J10 (magenta stick) and Arg61 of 14-3-3ε (firebrick sticks) while the phosphate moiety, two glutamic acid residues, and water molecules (red spheres) are in the surrounding environment (ii).

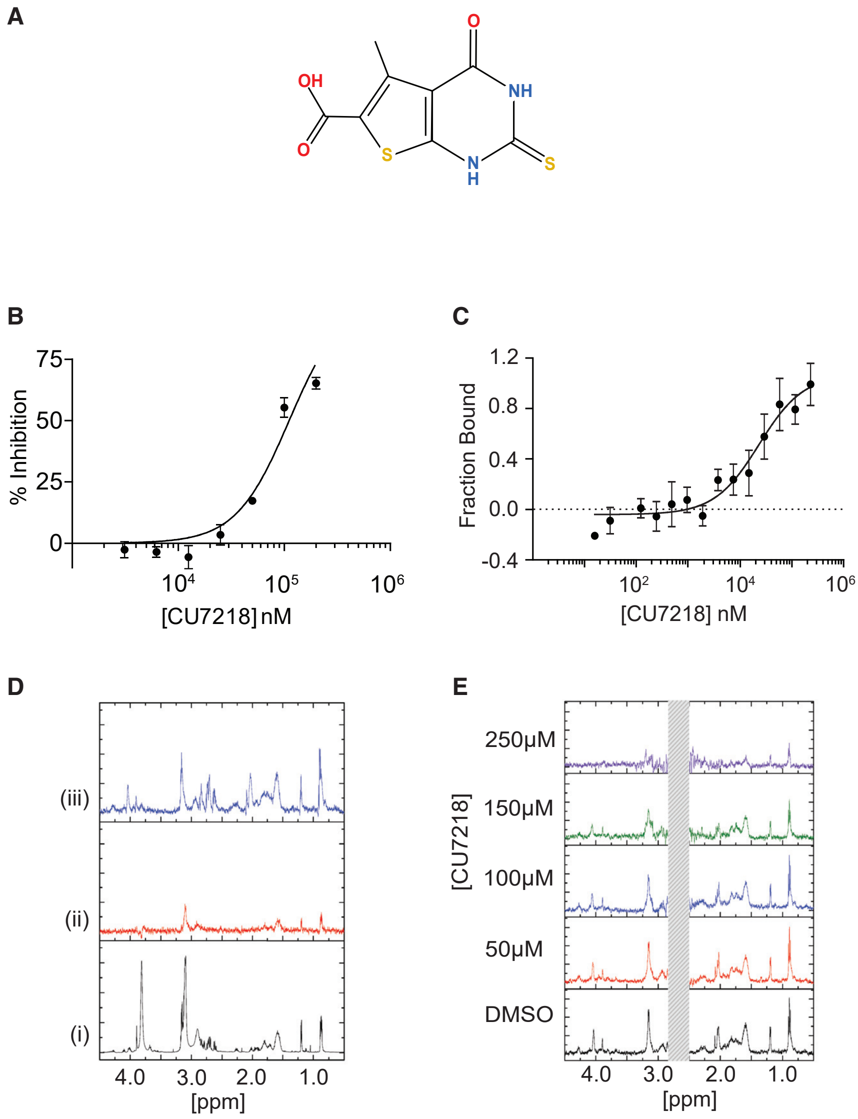

Figure 6. Identification and validation of a small-molecule inhibitor of 14-3-3

(A) Chemical structure of primary hit CU7218.

(B) Dose-response curve for CU7218 in the FP assay. Data are the mean of three independent experiments ± SD.

(C) Dose-response curve for CU7218 in the competitive MST assay. Data are the mean of three independent experiments ± SD.

(D) ^{1}H spectrum of 9J10 peptide in D_2O (i), the STD-NMR spectrum of 9J10 in the absence of 14-3-3 (ii), and the STD-NMR spectrum of 9J10 in the presence of 14-3-3 (iii).

(E) The STD-NMR spectrum of 9J10 (100 μM) in the presence of 14-3-3 (10 μM) plus addition of 2% DMSO/D_2O, or the indicated concentration of CU7218 to the solution of 9J10 and 14-3-3. Fresh samples were used for each experiment, and the solvent peaks are masked with a gray-colored box.

seven C-terminal amino acids essential for its interaction with 14-3-3 (termed p9J10Min) and labeled with tetramethylrhodamine (TAMRA) as a fluorescence polarization (FP) probe. We established an assay to measure changes in FP to detect the binding of TAMRA-p9J10Min to 14-3-3. Consistent with expectation, TAMRA-p9J10Min binds to purified 14-3-3ε with a K_D of 1.47 ± 0.71 μM in this assay (Figure S6A), and an unlabeled 9J10-derived phosphopeptide effectively competed for 14-3-3 binding, with a half-maximal inhibitory concentration (IC_{50}) of 9.8 ± 1.2 μM (Figure S6B).

A diversity library of 14,080 compounds (Huggins et al., 2011) was used for screening. Elements of the library conform to drug-like physicochemical properties with an average molecular weight of 370 Da, cLogP (octanol/water partition coefficient) value of 2.7, cLogS (aqueous solubility) of −4.3, and TPSA (total polar surface area) of 86 Å^2. Ten primary hits were identified showing >30% inhibition at 125 μM concentration, and of these the five commercially available primary hit compounds were reordered and tested in the FP assay to confirm activity. One of these compounds, CU7218 (Figure 6A), was selected for further validation. CU7218 is a fragment-like molecule with a molecular weight of 241 Da, which contains a carboxylate group attached to a heterocyclic five-membered ring and a pyrimidine ring (Figure 6A).

Consistent with a binding mode at the phosphopeptide-binding site of 14-3-3, compound CU7218 inhibits the interaction between 9J10 and 14-3-3ε in the FP assay, with an IC_{50} of 111 μM (Figure 6B). The compound was also active when tested in an orthogonal competitive microscale thermophoresis (MST) assay (Seidel et al., 2013), where it inhibited the interaction between 9J10 and 14-3-3 with an IC_{50} of 26.5 μM (Figure 6C). Thus, we confirmed the on-target activity of CU7218 using two distinct binding assays. Notably, CU7218 failed to disrupt substrate recognition by a different phosphopeptide-binding domain, the polo-box domain of human polo-like kinase 1 (PLK1) (Narvaez et al., 2017), speaking to its selectivity for the 14-3-3 phosphopeptide-binding site.

Saturation transfer difference (STD) nuclear magnetic resonance (NMR) provides further evidence for the binding activity of CU7218. The STD spectra of the 9J10 phosphopeptide (LNRTPGRRRNpSN) revealed a clear signal in the presence of 14-3-3ε (Figure 6D), indicating binding between peptide and protein. Peaks at 0.89 ppm and 1.19 ppm, identified as the methyl groups of Leu1 and Thr4, respectively, showed the strongest signals. A control experiment in the presence of 2% deuterated DMSO (DMSO-d_6) showed similar spectra, indicating that DMSO has no effect on the protein-peptide interaction. By contrast, CU7218 markedly reduced the 9J10 STD signal intensity (Figure 6E) from a concentration of 150 μM upward, indicating that the compound inhibits the interaction between 9J10

and 14-3-3. When taken together, these results exemplify how a bioactive peptide discovered by phenotypic screening can serve as a springboard to identify small-molecule inhibitors of a protein-protein interaction.

Further work beyond the scope of this paper will now be required to optimize the potency and physicochemical properties of compounds identified in this way before their cellular activity and selectivity can be fully assessed. It will be important to test whether such optimized compounds can displace FOXO3a and other substrate proteins from 14-3-3 and reproduce the cellular phenotypes induced by 9J10 expression. The low molecular weight and fragment-like structure of CU7218 offers ample scope to improve its potency through chemical elaboration.

DISCUSSION

Here we describe "protein interference," an approach whereby libraries of peptides encoded in evolutionarily diverse prokaryal genomes are used to interfere with macromolecular interactions essential for complex biochemical pathways in human cells, yielding phenotypes that can be scored in high-throughput screens. Our results exemplify several features underlying protein interference. We demonstrate that prokaryal peptides are capable of interfering with human protein-protein interactions and may do so via structural characteristics distinct from those typical of cognate human proteins. We observe in one example screen that prokaryal peptide libraries yield a relatively high "hit rate" compared with random synthetic peptide libraries. Notably, we illustrate a complete workflow whereby (1) a high-throughput genetic screen using such a library identifies a target macromolecular interaction essential for a complex phenotype in human cells, (2) the interaction between a "hit" peptide and its human target protein is characterized to enable structural and biological validation, and (3) the discovery of drug-like small molecules that inhibit the interaction yields starting points for further chemical exploration. Thus, our work to exemplify "protein interference" provides the foundation for a widely applicable method to parse macromolecular interactions in complex biological pathways and identify molecular targets for systems biology and therapeutics development.

Several features of this approach distinguish it from current methods for target identification through phenotypic screening. We have harnessed the evolutionary diversity of peptides naturally encoded in prokaryal genomes as probes to bind to, and interfere with, human protein function. Hence, unlike phenotypic screens using gene targeting or RNA depletion, which inactivate or remove target proteins, our approach mimics more closely the selective functional consequences of target engagement by a drug. In particular, the probe-target protein interaction defines an active functional site within the human protein target more directly and rapidly than is feasible with other methods, facilitating efforts to assess target "druggability." However, in contrast to direct phenotypic screening using libraries of chemical compounds, the size and structure of the natural peptides that we use as probes explore distinct structural space that probably conforms more closely to protein evolution. Moreover, our screening methodology facilitates the rapid identification and validation of molecular targets, typically a challenge in phenotypic screens using chemical libraries (Kubota et al., 2019; Moffat et al., 2017).

We have exemplified "protein interference" using the FOXO3a signaling pathway, a tumor-suppressive network that is frequently inactivated in human cancers by post-translational modifications rather than genetic mutations (Dansen and Burgering, 2008; Yang and Hung, 2009; Zanella and Carnero, 2009). We show that a relatively small library of $\sim 7 \times 10^3$ prokaryal peptides nevertheless yields ~13 bioactive probes capable of inducing the nuclear translocation of FOXO3a. The high "hit rate" (~0.2%) in this example screen, while anecdotal, is encouraging, and raises the possibility that libraries of evolutionarily diverse, biologically derived peptides encoded in simple genomes may offer advantages over random synthetic peptides for phenotypic screening.

We identify the target of one such peptide probe, 9J10, as the 14-3-3 protein family, which are known mediators of FOXO3a localization and implicated in a wide range of human diseases including cancer, neurodegeneration, and cystic fibrosis (Kaplan et al., 2017; Stevers et al., 2016; Wilker and Yaffe, 2004). 9J10 is encoded in the genome of *S. avermitilis*, an Actinobacterium that has proved to be a rich source of bioactive compounds including the avermectins, polycyclic lactones with anti-parasitic activity (Campbell, 2012). Interestingly, the 9J10/14-3-3 interaction depends on the phosphorylation of a Ser residue in the peptide, illustrating the potential of our approach to interfere with human protein function via post-translational modifications of the probe.

We demonstrate the biological activity of 9J10 in human cells using a combination of approaches. 9J10 expression disrupts the interaction between 14-3-3 and FOXO3a. RNA sequencing and promoter motif enrichment reveals that 9J10 expression modulates a transcriptional program including multiple known FOXO-dependent transcripts, and overlaps significantly with transcriptional changes induced by a constitutively active FOXO3a-AAA mutant protein.

Interestingly, 9J10 expression also modulates additional transcripts neither altered by FOXO3a-AAA expression nor possessing FOXO-binding promoter motifs. These findings suggest that 9J10 may activate transcription factors other than FOXO3a that are engaged by the 14-3-3 family of signal regulators. Indeed, multiple transcription factors whose expression or activity can be altered in cancer cells are known to bind the 14-3-3 proteins (Falcicchio et al., 2020; Mackintosh, 2004; Moon et al., 2017).

9J10 expression suppresses the growth of multiple cancer cell lines. However, FOXO3a depletion using RNAi suggests that this biological response is independent of FOXO activation. Our findings raise the hypothesis that 9J10 exerts its cancer-growth-suppressive effects by displacing a factor other than FOXO3a from its engagement by 14-3-3. Future experiments to identify this factor could provide fresh insight into the mechanisms underlying tumor-suppressive transcriptional programs regulated by the 14-3-3 protein family.

A crystal structure of the 9J10/14-3-3 complex reveals that 9J10 adopts a binding mode somewhat distinct from that of cognate 14-3-3 binding peptides from human proteins. In particular, two consecutive Arg residues in 9J10 facilitate the interaction of its phospho-Ser moiety with the canonical phosphate-binding site in 14-3-3, and the peptide backbone of 9J10 deviates from the canonical peptide-binding groove to engage residues in an

adjacent helical region of 14-3-3. These features suggest the potential to uncover new, functionally relevant binding sites using prokaryal peptides as binding probes for human proteins. In other words, protein interference screens seek novel, druggable sites on human proteins within the cellular milieu.

We illustrate in this work a complete workflow for protein interference, starting with a high-throughput phenotypic screen, followed by target identification and structural as well as biological validation, which enables the discovery of small-molecule ligands that displace 9J10 from 14-3-3. Thus, using a bioactive peptide as a probe for target identification and then as a springboard for small-molecule screening streamlines the throughput of phenotypic screens and the deconvolution of potential targets. Such an approach is highly scalable, so the limited proof-of-concept screen reported here provides a blueprint for larger screens of higher complexity that promise to expand both our knowledge of biologically relevant macromolecular interactions in human cells and our currently limited repertoire of "druggable" targets.

SIGNIFICANCE

Macromolecular interactions between human proteins underlie normal cellular regulation and its derangement in diseases. Protein-protein interactions represent a vast yet untapped potential repertoire of therapeutic targets. New technologies are needed to parse this repertoire and identify potentially druggable protein-protein interactions critical for cellular pathways. Here, we describe "protein interference" as a tool to seek novel druggable sites, whose engagement in living cells modulates rate-limiting steps in the pathways responsible for complex cellular phenotypes. "Protein interference" utilizes structurally diverse libraries of short peptides derived from natural genomes as probes. Genetically encoded protein interference probes, expressed in living cells, seek cognate binding sites in human proteins and disrupt the macromolecular interactions regulating protein function. Such disruption in turn alters cellular phenotypes, enabling rapid genetic screens to dissect rate-limiting protein-protein interactions that are essential for intricate pathways. Notably, the interaction of the protein interference probe with its cellular protein target defines a potential binding site for new drugs, including peptides, macrocyclics, or small molecules, and provides a facile approach to screen for compounds that displace the probe from its protein target. These features of protein interference enable the rapid identification of druggable sites in target proteins, and validation of the structural features and biological consequences of binding-site engagement. The work we report here provides a complete workflow for protein interference technology, from a high-throughput phenotypic screen to the identification and structural and biological validation of new targets, and the discovery of small-molecule ligands that displace the protein interference probe from its cellular target. This work provides a blueprint for future screens using larger protein interference libraries of greater complexity to seek and validate novel druggable targets for systems biology and development of therapeutics for human diseases.

STAR★METHODS

Detailed methods are provided in the online version of this paper and include the following:

- KEY RESOURCES TABLE
- RESOURCE AVAILABILITY
  - Lead contact
  - Materials availability
  - Data and code availability
- EXPERIMENTAL MODEL AND SUBJECT DETAILS
  - Cell lines
- METHOD DETAILS
  - GFP-FOXO3a vectors
  - Peptide library preparation
  - FOXO3a relocalisation assay
  - Compound treatment
  - siRNA
  - Protein interference screen
  - SDS-PAGE and western blotting
  - V5 immunoprecipitation
  - Silver staining
  - Mass spectrometry
  - Yeast 2 hybrid screening
  - FLAG immunoprecipitation
  - Immunofluorescence for endogenous FOXO3a
  - 9J10 mutant plasmid constructs
  - Live cell confluency measurements
  - Forkhead luciferase reporter assay
  - RNA sequencing: sample preparation
  - RNA sequencing: data analysis
  - Transcription factor motif enrichment analysis
  - Protein expression and purification
  - Isothermal titration calorimetry
  - Crystallization and structure determination
  - Fluorescence polarisation (FP) assay
  - Microscale Thermophoresis (MST)
  - STD-NMR
- QUANTIFICATION AND STATISTICAL ANALYSIS

SUPPLEMENTAL INFORMATION

Supplemental information can be found online at https://doi.org/10.1016/j.chembiol.2021.05.009.

ACKNOWLEDGMENTS

This work was funded by program grants MC_UU_12022/1 and MC_UU_12022/8 to A.R.V. from the UK Medical Research Council, and by an SFC grant BT/PR7222/MED/31/1901/2012 to A.R.V. from the Department of Biotechnology, Govt. of India. We thank members of the Venkitaraman laboratory (particularly Robert Mahen) for helpful discussions and critical review of this work.

AUTHOR CONTRIBUTIONS

A.E., B.S.H., A.T.C., G.J.M., and A.R.V. conceived and carried out phenotypic screen, peptide hit identification, validation, and biological characterization. C.M., V.K., S.G., B.S.H., G.S., and A.R.V. conceived and carried out assay development, screening, and hit validation for small-molecule discovery. S.M., S.S., S.B., K.B., N.K., and A.R.V. determined and analyzed the structure of the 9J10/14-3-3 complex. N.K., M.A.H., and T.K.P. carried out biophysical

assays. N.M. and P.M.W. prepared and provided the library of bioactive peptides. M.M., M.C., and C.J.T. validated 9J10/14-3-3 interaction using the yeast two-hybrid assay. A.E. and A.R.V. wrote the paper with assistance from the other authors.

DECLARATION OF INTERESTS

A.R.V., C.J.T., and G.J.M. are founders of PhoreMost Ltd. C.J.T., M.M., and M.C. are employees and shareholders of the company, A.R.V. is a shareholder and the chief scientific adviser, and G.J.M. and B.S.H. are shareholders. P.M.W. is a founder and shareholder, and N.M. a shareholder, of PYC Therapeutics.

Received: August 29, 2020
Revised: March 3, 2021
Accepted: May 14, 2021
Published: June 9, 2021

REFERENCES

Accili, D., and Arden, K.C. (2004). FoxOs at the crossroads of cellular metabolism, differentiation, and transformation. Cell *117*, 421–426.

Battye, T.G.G., Kontogiannis, L., Johnson, O., Powell, H.R., and Leslie, A.G.W. (2011). iMOSFLM: a new graphical interface for diffraction-image processing with MOSFLM. Acta Crystallogr. D Biol. Crystallogr. *67*, 271–281.

Blum, J.H., Dove, S.L., Hochschild, A., and Mekalanos, J.J. (2000). Isolation of peptide aptamers that inhibit intracellular processes. Proc. Natl. Acad. Sci. U S A *97*, 2241–2246.

Borisova, M.E., Voigt, A., Tollenaere, M.A.X., Sahu, S.K., Juretschke, T., Kreim, N., Mailand, N., Choudhary, C., Bekker-Jensen, S., Akutsu, M., et al. (2018). p38-MK2 signaling axis regulates RNA metabolism after UV-light-induced DNA damage. Nat. Commun. *9*, 1017.

Brunet, A., Bonni, A., Zigmond, M.J., Lin, M.Z., Juo, P., Hu, L.S., Anderson, M.J., Arden, K.C., Blenis, J., and Greenberg, M.E. (1999). Akt promotes cell survival by phosphorylating and inhibiting a Forkhead transcription factor. Cell *96*, 857–868. https://doi.org/10.1016/s0092-8674(00)80595-4.

Brunet, A., Kanai, F., Stehn, J., Xu, J., Sarbassova, D., Frangioni, J.V., Dalal, S.N., DeCaprio, J.A., Greenberg, M.E., and Yaffe, M.B. (2002). 14-3-3 transits to the nucleus and participates in dynamic nucleocytoplasmic transport. J. Cell Biol. *156*, 817–828.

Burgering, B.M., and Kops, G.J. (2002). Cell cycle and death control: long live Forkheads. Trends Biochem. Sci. *27*, 352–360. https://doi.org/10.1016/s0968-0004(02)02113-8.

Campbell, W.C. (2012). Ivermectin and Abamectin (Springer Science & Business Media).

Caponigro, G., Abedi, M.R., Hurlburt, A.P., Maxfield, A., Judd, W., and Kamb, A. (1998). Transdominant genetic analysis of a growth control pathway. Proc. Natl. Acad. Sci. U S A *95*, 7508–7513.

Cautain, B., Castillo, F., Musso, L., Ferreira, B.I., de Pedro, N., Rodriguez Quesada, L., Machado, S., Vicente, F., Dallavalle, S., and Link, W. (2016). Discovery of a novel, isothiazolonaphthoquinone-based small molecule activator of FOXO nuclear-cytoplasmic shuttling. PLoS One *11*, e0167491.

Chaudhri, M., Scarabel, M., and Aitken, A. (2003). Mammalian and yeast 14-3-3 isoforms form distinct patterns of dimers in vivo. Biochem. Biophys. Res. Commun. *300*, 679–685.

Chung, N., Zhang, X.D., Kreamer, A., Locco, L., Kuan, P.-F., Bartz, S., Linsley, P.S., Ferrer, M., and Strulovici, B. (2008). Median absolute deviation to improve hit selection for genome-scale RNAi screens. J. Biomol. Screen. *13*, 149–158.

Clarke, L., and Carbon, J. (1976). A colony bank containing synthetic Col El hybrid plasmids representative of the entire *E. coli* genome. Cell *9*, 91–99.

Dansen, T.B., and Burgering, B.M.T. (2008). Unravelling the tumor-suppressive functions of FOXO proteins. Trends Cell. Biol. *18*, 421–429.

Dong, S., Kang, S., Gu, T.-L., Kardar, S., Fu, H., Lonial, S., Khoury, H.J., Khuri, F., and Chen, J. (2007). 14-3-3 Integrates prosurvival signals mediated by the AKT and MAPK pathways in ZNF198-FGFR1-transformed hematopoietic cells. Blood *110*, 360–369.

Emsley, P., and Cowtan, K. (2004). Coot: model-building tools for molecular graphics. Acta Crystallogr. D Biol. Crystallogr. *60*, 2126–2132.

Evans, P. (2006). Scaling and assessment of data quality. Acta Crystallogr. D Biol. Crystallogr. *62*, 72–82.

Falcicchio, M., Ward, J.A., Macip, S., and Doveston, R.G. (2020). Regulation of p53 by the 14-3-3 protein interaction network: new opportunities for drug discovery in cancer. Cell Death Discov. *6*, 126.

Farhan, M., Wang, H., Gaur, U., Little, P.J., Xu, J., and Zheng, W. (2017). FOXO signaling pathways as therapeutic targets in cancer. Int. J. Biol. Sci. *13*, 815–827.

Fresno Vara, J.A., Casado, E., de Castro, J., Cejas, P., Belda-Iniesta, C., and González-Barón, M. (2004). PI3K/Akt signalling pathway and cancer. Cancer Treat. Rev. *30*, 193–204. https://doi.org/10.1016/j.ctrv.2003.07.007.

Furuyama, T., Nakazawa, T., Nakano, I., and Mori, N. (2000). Identification of the differential distribution patterns of mRNAs and consensus binding sequences for mouse DAF-16 homologues. Biochem. J. *349*, 629–634.

Gross, D.N., van den Heuvel, A.P.J., and Birnbaum, M.J. (2008). The role of FoxO in the regulation of metabolism. Oncogene *27*, 2320–2336.

Hart, G.T., Ramani, A.K., and Marcotte, E.M. (2006). How complete are current yeast and human protein-interaction networks? Genome Biol. *7*, 120.

Ho Sui, S.J., Fulton, D.L., Arenillas, D.J., Kwon, A.T., and Wasserman, W.W. (2007). oPOSSUM: integrated tools for analysis of regulatory motif over-representation. Nucleic Acids Res. *35*, W245–W252.

Hu, M.C.-T., Lee, D.-F., Xia, W., Golfman, L.S., Ou-Yang, F., Yang, J.-Y., Zou, Y., Bao, S., Hanada, N., Saso, H., et al. (2004). IkappaB kinase promotes tumorigenesis through inhibition of forkhead FOXO3a. Cell *117*, 225–237.

Huggins, D.J., Venkitaraman, A.R., and Spring, D.R. (2011). Rational methods for the selection of diverse screening compounds. ACS Chem. Biol. *6*, 208–217.

Ishihama, Y., Oda, Y., Tabata, T., Sato, T., Nagasu, T., Rappsilber, J., and Mann, M. (2005). Exponentially modified protein abundance index (emPAI) for estimation of absolute protein amount in proteomics by the number of sequenced peptides per protein. Mol. Cell. Proteomics *4*, 1265–1272.

Kaplan, A., Ottmann, C., and Fournier, A.E. (2017). 14-3-3 adaptor protein-protein interactions as therapeutic targets for CNS diseases. Pharmacol. Res. *125*, 114–121.

Kovacs, I.A., Luck, K., Spirohn, K., Wang, Y., Pollis, C., Schlabach, S., Bian, W., Kim, D.K., Kishore, N., Hao, T., et al. (2019). Network-based prediction of protein interactions. Nat. Commun. *10*, 1240.

Kubota, K., Funabashi, M., and Ogura, Y. (2019). Target deconvolution from phenotype-based drug discovery by using chemical proteomics approaches. Biochim. Biophys. Acta *1867*, 22–27.

Kwon, A.T., Arenillas, D.J., Hunt, R.W., and Wasserman, W.W. (2012). oPOSSUM-3: advanced analysis of regulatory motif over-representation across genes or ChIP-seq datasets. G3 (Bethesda) *2*, 987–1002.

Laflamme, C., Galan, J.A., Ben El Kadhi, K., Méant, A., Zeledon, C., Carréno, S., Roux, P.P., and Emery, G. (2017). Proteomics screen identifies class I Rab11 family interacting proteins as key regulators of cytokinesis. Mol. Cell. Biol. *37*, e00278-16.

Link, W., Oyarzabal, J., Serelde, B.G., Albarran, M.I., Rabal, O., Cebriá, A., Alfonso, P., Fominaya, J., Renner, O., Peregrina, S., et al. (2009). Chemical interrogation of FOXO3a nuclear translocation identifies potent and selective inhibitors of phosphoinositide 3-kinases. J. Biol. Chem. *284*, 28392–28400.

Liu, Y., Ao, X., Ding, W., Ponnusamy, M., Wu, W., Hao, X., Yu, W., Wang, Y., Li, P., and Wang, J. (2018). Critical role of FOXO3a in carcinogenesis. Mol. Cancer *17*, 104.

Luck, K., Sheynkman, G.M., Zhang, I., and Vidal, M. (2017). Proteome-scale human interactomics. Trends Biochem. Sci. *42*, 342–354.

Mackintosh, C. (2004). Dynamic interactions between 14-3-3 proteins and phosphoproteins regulate diverse cellular processes. Biochem. J. *381*, 329–342.

Madeira, F., Tinti, M., Murugesan, G., Berrett, E., Stafford, M., Toth, R., Cole, C., MacKintosh, C., and Barton, G.J. (2015). 14-3-3-Pred: improved methods to predict 14-3-3-binding phosphopeptides. Bioinformatics *31*, 2276–2283.

McCoy, A.J., Grosse-Kunstleve, R.W., Adams, P.D., Winn, M.D., Storoni, L.C., and Read, R.J. (2007). Phaser crystallographic software. J. Appl. Crystallogr. *40*, 658–674.

Milech, N., and Watt, P. (2012). The construction of ''phylomer'' peptide libraries as a rich source of potent inhibitors of protein/protein interactions. In Therapeutic Proteins: Methods and Protocols, V. Voynov and J.A. Caravella, eds. (Humana Press), pp. 43–60.

Moffat, J.G., Vincent, F., Lee, J.A., Eder, J., and Prunotto, M. (2017). Opportunities and challenges in phenotypic drug discovery: an industry perspective. Nat. Rev. Drug Discov. *16*, 531–543.

Molzan, M., Weyand, M., Rose, R., and Ottmann, C. (2012). Structural insights of the MLF1/14-3-3 interaction. FEBS J. *279*, 563–571.

Moon, S., Kim, W., Kim, S., Kim, Y., Song, Y., Bilousov, O., Kim, J., Lee, T., Cha, B., Kim, M., et al. (2017). Phosphorylation by NLK inhibits YAP-14-3-3-interactions and induces its nuclear localization. EMBO Rep. *18*, 61–71.

Mortazavi, A., Williams, B.A., McCue, K., Schaeffer, L., and Wold, B. (2008). Mapping and quantifying mammalian transcriptomes by RNA-seq. Nat. Methods *5*, 621–628.

Murshudov, G.N., Skubák, P., Lebedev, A.A., Pannu, N.S., Steiner, R.A., Nicholls, R.A., Winn, M.D., Long, F., and Vagin, A.A. (2011). REFMAC5 for the refinement of macromolecular crystal structures. Acta Crystallogr. D Biol. Crystallogr. *67*, 355–367.

Muslin, A.J., Tanner, J.W., Allen, P.M., and Shaw, A.S. (1996). Interaction of 14-3-3 with signaling proteins is mediated by the recognition of phosphoserine. Cell *84*, 889–897.

Narvaez, A.J., Ber, S., Crooks, A., Emery, A., Hardwick, B., Guarino Almeida, E., Huggins, D.J., Perera, D., Roberts-Thomson, M., Azzarelli, R., et al. (2017). Modulating protein-protein interactions of the mitotic polo-like kinases to target mutant KRAS. Cell Chem. Biol. *24*, 1017–1028.e7.

Ni, D., Ma, X., Li, H.Z., Gao, Y., Li, X.T., Zhang, Y., Ai, Q., Zhang, P., Song, E.L., Huang, Q.B., et al. (2014). Downregulation of FOXO3a promotes tumor metastasis and is associated with metastasis-free survival of patients with clear cell renal cell carcinoma. Clin Cancer Res. *20*, 1779–1790. https://doi.org/10.1158/1078-0432.CCR-13-1687.

Nim, S., Jeon, J., Corbi-Verge, C., Seo, M.H., Ivarsson, Y., Moffat, J., Tarasova, N., and Kim, P.M. (2016). Pooled screening for antiproliferative inhibitors of protein-protein interactions. Nat. Chem. Biol. *12*, 275–281.

Norman, T.C., Smith, D.L., Sorger, P.K., Drees, B.L., O'Rourke, S.M., Hughes, T.R., Roberts, C.J., Friend, S.H., Fields, S., and Murray, A.W. (1999). Genetic selection of peptide inhibitors of biological pathways. Science *285*, 591–595.

Pozuelo Rubio, M., Geraghty, K.M., Wong, B.H., Wood, N.T., Campbell, D.G., Morrice, N., and Mackintosh, C. (2004). 14-3-3-affinity purification of over 200 human phosphoproteins reveals new links to regulation of cellular metabolism, proliferation and trafficking. Biochem. J. *379*, 395–408.

Ramaswamy, S., Nakamura, N., Sansal, I., Bergeron, L., and Sellers, W.R. (2002). A novel mechanism of gene regulation and tumor suppression by the transcription factor FKHR. Cancer Cell *2*, 81–91.

Reagan-Shaw, S., and Ahmad, N. (2007). The role of Forkhead-box Class O (FoxO) transcription factors in cancer: a target for the management of cancer. Toxicol. Appl. Pharmacol. *224*, 360–368. https://doi.org/10.1016/j.taap.2006.12.003.

Rittinger, K., Budman, J., Xu, J., Volinia, S., Cantley, L.C., Smerdon, S.J., Gamblin, S.J., and Yaffe, M.B. (1999). Structural analysis of 14-3-3 phosphopeptide complexes identifies a dual role for the nuclear export signal of 14-3-3 in ligand binding. Mol. Cell *4*, 153–166.

Santo, E.E., Stroeken, P., Sluis, P.V., Koster, J., Versteeg, R., and Westerhout, E.M. (2013). FOXO3a is a major target of inactivation by PI3K/AKT signaling in aggressive neuroblastoma. Cancer Res. *73*, 2189–2198.

Seidel, S.A.I., Dijkman, P.M., Lea, W.A., van den Bogaart, G., Jerabek-Willemsen, M., Lazic, A., Joseph, J.S., Srinivasan, P., Baaske, P., Simeonov, A., et al. (2013). Microscale thermophoresis quantifies biomolecular interactions under previously challenging conditions. Methods *59*, 301–315.

Seoane, J., Le, H.-V., Shen, L., Anderson, S.A., and Massagué, J. (2004). Integration of Smad and forkhead pathways in the control of neuroepithelial and glioblastoma cell proliferation. Cell *117*, 211–223.

Southern, J.A., Young, D.F., Heaney, F., Baumgärtner, W.K., and Randall, R.E. (1991). Identification of an epitope on the P and V proteins of simian virus 5 that distinguishes between two isolates with different biological characteristics. J. Gen. Virol. *72*, 1551–1557.

Stevers, L.M., Lam, C.V., Leysen, S.F.R., Meijer, F.A., van Scheppingen, D.S., de Vries, R.M.J.M., Carlile, G.W., Milroy, L.G., Thomas, D.Y., Brunsveld, L., et al. (2016). Characterization and small-molecule stabilization of the multisite tandem binding between 14-3-3 and the R domain of CFTR. Proc. Natl. Acad. Sci. U S A *113*, E1152–E1161.

Tinti, M., Madeira, F., Murugesan, G., Hoxhaj, G., Toth, R., and Mackintosh, C. (2014). ANIA: ANnotation and Integrated Analysis of the 14-3-3 interactome. Database (Oxford) *2014*, bat085.

Van Der Heide, L.P., Hoekman, M.F.M., and Smidt, M.P. (2004). The ins and outs of FoxO shuttling: mechanisms of FoxO translocation and transcriptional regulation. Biochem. J. *380*, 297–309.

Vivanco, I., and Sawyers, C.L. (2002). The phosphatidylinositol 3-Kinase AKT pathway in human cancer. Nat. Rev. Cancer *2*, 489–501. https://doi.org/10.1038/nrc839.

Watt, P.M. (2006). Screening for peptide drugs from the natural repertoire of biodiverse protein folds. Nat. Biotechnol. *24*, 177–183.

Watt, P.M., Milech, N., and Stone, S.R. (2017). Structure-diverse Phylomer libraries as a rich source of bioactive hits from phenotypic and target directed screens against intracellular proteins. Curr. Opin. Chem. Biol. *38*, 127–133.

Webb, A.E., Kundaje, A., and Brunet, A. (2016). Characterization of the direct targets of FOXO transcription factors throughout evolution. Aging Cell *15*, 673–685.

Wilker, E., and Yaffe, M.B. (2004). 14-3-3 Proteins—a focus on cancer and human disease. J. Mol. Cell. Cardiol. *37*, 633–642.

Winn, M.D., Ballard, C.C., Cowtan, K.D., Dodson, E.J., Emsley, P., Evans, P.R., Keegan, R.M., Krissinel, E.B., Leslie, A.G.W., McCoy, A., et al. (2011). Overview of the CCP4 suite and current developments. Acta Crystallogr. D Biol. Crystallogr. *67*, 235–242.

Xie, C., Song, L.B., Wu, J.H., Li, J., Yun, J.P., Lai, J.M., Xie, D.Y., Lin, B.L., Yuan, Y.F., Li, M., and Gao, Z.L. (2012). Upregulator of cell proliferation predicts poor prognosis in hepatocellular carcinoma and contributes to hepatocarcinogenesis by downregulating FOXO3a. PLoS One *7*, e40607. https://doi.org/10.1371/journal.pone.0040607.

Xu, X., Leo, C., Jang, Y., Chan, E., Padilla, D., Huang, B.C., Lin, T., Gururaja, T., Hitoshi, Y., Lorens, J.B., et al. (2001). Dominant effector genetics in mammalian cells. Nat. Genet. *27*, 23–29.

Yaffe, M.B., Rittinger, K., Volinia, S., Caron, P.R., Aitken, A., Leffers, H., Gamblin, S.J., Smerdon, S.J., and Cantley, L.C. (1997). The structural basis for 14-3-3:phosphopeptide binding specificity. Cell *91*, 961–971.

Yang, J.-Y., and Hung, M.-C. (2009). A new fork for clinical application: targeting forkhead transcription factors in cancer. Clin. Cancer Res. *15*, 752–757.

Yang, J.-Y., Zong, C.S., Xia, W., Yamaguchi, H., Ding, Q., Xie, X., Lang, J.-Y., Lai, C.-C., Chang, C.-J., Huang, W.-C., et al. (2008). ERK promotes tumorigenesis by inhibiting FOXO3a via MDM2-mediated degradation. Nat. Cell Biol. *10*, 138–148.

Yang, J.-Y., Chang, C.-J., Xia, W., Wang, Y., Wong, K.-K., Engelman, J.A., Du, Y., Andreeff, M., Hortobagyi, G.N., and Hung, M.-C. (2010). Activation of FOXO3a is sufficient to reverse mitogen-activated protein/extracellular signal-regulated kinase kinase inhibitor chemoresistance in human cancer. Cancer Res. *70*, 4709–4718.

Yang, X., Lee, W.H., Sobott, F., Papagrigoriou, E., Robinson, C.V., Grossmann, J.G., Sundström, M., Doyle, D.A., and Elkins, J.M. (2006).

CellPress
OPEN ACCESS

Structural basis for protein-protein interactions in the 14-3-3 protein family. Proc. Natl. Acad. Sci. U S A *103*, 17237–17242.

Yang, X.B., Zhao, J.J., Huang, C.Y., Wang, Q.J., Pan, K., Wang, D.D., Pan, Q.Z., Jiang, S.S., Lv, L., Gao, X., et al. (2013). Decreased expression of the FOXO3a gene is associated with poor prognosis in primary gastric adenocarcinoma patients. PLoS One *8*, e78158. https://doi.org/10.1371/journal.pone.0078158.

Zanella, F., and Carnero, A. (2009). Adding more content to screening: reactivation of FOXO as a therapeutic strategy. Clin. Transl. Oncol. *11*, 651–658.

Zanella, F., Rosado, A., García, B., Carnero, A., and Link, W. (2008). Chemical genetic analysis of FOXO nuclear-cytoplasmic shuttling by using image-based cell screening. Chembiochem *9*, 2229–2237.

Zhang, Q.C., Petrey, D., Deng, L., Qiang, L., Shi, Y., Thu, C.A., Bisikirska, B., Lefebvre, C., Accili, D., Hunter, T., et al. (2012). Structure-based prediction of protein-protein interactions on a genome-wide scale. Nature *490*, 556–560.

Zou, Y., Tsai, W.B., Cheng, C.J., Hsu, C., Chung, Y.M., Li, P.C., Lin, S.H., and Hu, M.C. (2008). Forkhead box transcription factor FOXO3a suppresses estrogen-dependent breast cancer cell proliferation and tumorigenesis. Breast Cancer Res. *10*, R21. https://doi.org/10.1186/bcr1872.

STAR★METHODS

KEY RESOURCES TABLE

REAGENT or RESOURCE	SOURCE	IDENTIFIER
Antibodies		
Rabbit polyclonal anti pan-14-3-3	Cell Signaling Technology	Cat#8312S; RRID:AB_10860606
Mouse monoclonal anti V5	Invitrogen	Cat#R96025; RRID:AB_159313
Rabbit monoclonal anti 14-3-3 gamma	Cell Signaling Technology	Cat#5522; RRID:AB_10827887
Rabbit monoclonal anti FOXO3a	Cell Signaling Technology	Cat#12829; RRID:AB_2636990
Mouse monoclonal anti FLAG	Sigma	Cat#F1804; RRID:AB_262044
Rabbit monoclonal anti HSP90	Cell Signaling Technology	Cat#4877S; RRID:AB_2233307
Mouse monoclonal anti pan-AKT	Cell Signaling Technology	Cat#2920S; RRID:AB_1147620
Rabbit monoclonal anti AKT-pSer473	Cell Signaling Technology	Cat#4060S; RRID:AB_2315049
Rabbit polyclonal anti FOXO3a-pSer253	Cell Signaling Technology	Cat#9466S; RRID:AB_2106674
Alexa Fluor 488 conjugated goat anti-rabbit IgG (A11034, Thermo Fisher Scientific	Thermo Fisher Scientific	Cat#A11034; RRID:AB_2576217
Sheep Anti-mouse IgG peroxidase conjugated	Fisher Scientific	Cat#NXA931; RRID:AB_772209
Donkey Anti-rabbit IgG peroxidase conjugated	Fisher Scientific	Cat#NA934; RRID:AB_772206
Mouse monoclonal anti beta-actin	Sigma Aldrich	Cat#A5441; RRID:AB_476744
Mouse monoclonal anti YAP	Cell Signaling Technology	Cat#12395S, RRID:AB_2797897
Mouse monoclonal anti p53	Santa Cruz Biotechnology	Cat#sc-126, RRID:AB_628082
Bacterial and virus strains		
ElectroMAX DH5alpha-E competent cells	Thermo Fisher Scientific	Cat#11319019
E. coli BL21 DE3	Sigma Aldrich	Cat#CMC0014
Chemicals, peptides, and recombinant proteins		
TAMRA-9J10Min peptide (5-TAMRA-GRRRN(p-Serine)N-acid)	Sigma Aldrich	Custom synthesis
TAMRA-9J10 peptide (5-TAMRA-LNRTPGRRRN(p-Serine)N-acid)	Sigma Aldrich	Custom synthesis
Unlabelled 9J10 peptide (LNRTPGRRRN(p-Serine)N-acid)	Sigma Aldrich	Custom synthesis
CU7218	Enamine	Cat#EN300-04536; CAS: 34330-04-6
V5 peptide	Abcam	Cat# ab15829
Hoechst 33342	Invitrogen	Cat#H3570; CAS: 875756-97-1
Leptomycin B	New England Biolabs	Cat#9676; CAS: 87081-35-4
PI-103	Selleckchem	Cat#S1038; CAS: 371935-74-9
BEZ235	Selleckchem	Cat#S1009; CAS: 915019-65-7
GSK2334470	Stratech Scientific	Cat#S7087-SEL; CAS: 1227911-45-6
LY294002	Merck Millipore	Cat#440202; CAS: 154447-36-6
GSK690693	Sigma Aldrich	Cat#SML0428; CAS: 937174-76-0
DMSO	Sigma Aldrich	Cat#D4540; CAS: 67-68-5
Critical commercial assays		
Pierce Silver Stain Kit	Thermo Fisher Scientific	Cat#24612
Wizard SV 96 Plasmid DNA Purification System	Promega	Cat#A2258
Anti-FLAG M2 Affinity Gel	Sigma Aldrich	Cat#A2220
Dynabeads Protein G	Invitrogen	Cat#10003D
jetPRIME transfection reagent	Polyplus	Cat#114-07
FuGENE 6 transfection reagent	Promega	Cat#E2691

(*Continued on next page*)

REAGENT or RESOURCE	SOURCE	IDENTIFIER
Continued		
RNAiMAX transfection reagent	Invitrogen	Cat#13778075
ECL Western Blotting Detection Reagents	Fisher Scientific	Cat#10155854
RNeasy Mini Kit	Qiagen	Cat#74104
Superscript III reverse transcriptase	Thermo Fisher Scientific	Cat#18010044
Quantitect Primer Assays against B2M	Qiagen	QT00088935
Quantitect Primer Assays against FASLG	Qiagen	QT00001281
Monolith Protein Labeling Kit	NanoTemper Technologies	Cat#MO-L011
Dual-Glo Luciferase Assay System	Promega	Cat#E2940
Deposited data		
Crystal Structure of 14-3-3 epsilon with 9J10 peptide	This study	PDB: 7C8E
RNA sequencing data (ArrayExpress)	This study	ArrayExpress: E-MTAB-10161
Experimental models: Cell lines		
U2OS	ECACC	Cat# 92022711
HEK293T	Authenticated using STR profiling	N/A
BT-549	A kind gift from C. Caldas (CRUK-CI)	N/A
MCF-7	Authenticated using STR profiling	N/A
SKOV-3	Authenticated using STR profiling	N/A
Oligonucleotides		
See Table S5 for siRNA target sequences	Various (see Table S5)	N/A
Recombinant DNA		
p3XFLAG-CMV-10	Sigma	Cat#E6758
pcDNALib	PYC Therapeutics	N/A
pGL4.20	Promega	Cat#E6751
pRL-TK	Promega	Cat#E2241
pcDNA3.1(+)	Thermo Fisher Scientific	Cat#V79020
pET28a	Novagen	Cat#69864-3
Full length 14-3-3 epsilon, codon-optimised for expression in *E. coli*	Thermo Fisher, GeneArt	Custom synthesis
Full length 14-3-3- epsilon cDNA clone	Cusabio	Cat#CL02628HU
Software and algorithms		
MetaXpress Translocation Enhanced Application Module	Molecular Devices	https://www.moleculardevices.com/products/cellular-imaging-systems/acquisition-and-analysis-software/metaxpress
GraphPad Prism	Graphpad Software Inc	https://www.graphpad.com/scientific-software/prism/
Origin 7.0	Origin Lab Corp	https://www.originlab.com/
Cellomics HCS Software, Molecular Translocation BioApplication V4	Thermo Fisher Scientific	N/A

RESOURCE AVAILABILITY

Lead contact

Further information and requests for resources and reagents should be directed to the lead contact, Ashok R. Venkitaraman (arv22@nus.edu.sg).

Materials availability

All unique/stable reagents generated in this study are available from the lead contact with a completed Materials Transfer Agreement as long as stocks remain available and reasonable compensation is provided by requestor to cover processing and shipment.

Data and code availability

RNA sequencing data in Figure 4 has been deposited in ArrayExpress (ArrayExpress: E-MTAB-10161). The crystal structure in Figure 5 has been deposited in the RCSB Protein Data Bank with the ID PDB: 7C8E.

EXPERIMENTAL MODEL AND SUBJECT DETAILS

Cell lines

HEK293T (female), MCF-7 (female) and U2OS (female) cells were cultured in Dulbecco modified Eagle medium (DMEM), BT-549 (female) cells were cultured in RPMI-1640 medium and SKOV3 (female) cells were cultured in McCoys 5A medium. All medium was supplemented with 10% fetal bovine serum (FBS). All cell lines were grown at 37°C in 5% CO2. All cell lines were authenticated using STR (Short Tandem Repeat) Profiling. To generate the stable U2OS-GFP-FOXO3a cell line, U2OS cells were transfected with a plasmid expressing Emerald-GFP-FOXO3a, and single cell clones stably expressing the transgene were selected by growth in the presence of 800 μg/ml G418. Clones were screened by eye on a fluorescence microscope to identify those in which localisation of EmGFP-FOXO3a was predominantly cytoplasmic and was homogenous between cells. One such single cell clone was selected for screening.

METHOD DETAILS

GFP-FOXO3a vectors

FOXO3 (NM_001455) Human Untagged Clone (Origene #SC119227) was used as template for conventional PCR with primers to add BamHI and XhoI restriction enzyme digestion sequences to the 5' and 3' ends respectively of FOXO3 and enable cloning into the mammalian expression vector pcDNA3.1(+) (Thermo Fisher Scientific #V79020). pcDNA6.2-GW/EmGFP-mIR (Thermo Fisher Scientific # K493600) was used as the template for conventional PCR for EmGFP. Primers to add KpnI and BamHI restriction enzyme digestion sequences to the 5' and 3' ends respectively of EmGFP were used to enable cloning of EmGFP into the pcDNA3.1(+)-FOXO3 vector to create a mammalian expression vector for GFP-FOXO3a fusion protein. The above vector was further modified by replacement of the wild type FOXO3a with the AAA mutant form (Ramaswamy et al., 2002) (Addgene #10709). Wild-type FOXO3a protein was excised by BamHI/XhoI digestion and the AAA-mutant was ligated in place to create a GFP-FOXO3a-AAA fusion protein. The GFP-FOXO3a vector was further modified to produce a control vector expressing GFP only: Wild-type FOXO3a protein was excised by BamHI/XhoI digestion and a Klenow fill-in was performed. The resultant linear vector was autoligated to form a GFP only control vector. All vectors were verified by Sanger sequencing.

Peptide library preparation

Genomic DNA from sequenced bacterial and archaeal genomes (see Table S1) was obtained by request from the American Type Tissue Culture and used as template for random low-temperature linear amplification with Klenow fragment by primer extension used a modified tagged-random primer amplification (TRPA) (Milech and Watt, 2012). The TRPA protocol was designed so that the oligonucleotides would anneal completely to highly AT-rich sequences and minimize bias, and involves four rounds of Klenow-mediated low temperature extension from oligos ending in random nucleotides. Amplified oligo fragments are tagged with an MfeI restriction site for compatible cloning into the EcoRI sites of the mammalian expression vector pcDNALib (containing an N-terminal V5 tag), killing the EcoRI site. The library complexity was determined to be 1.2×10^8 cfu (colony forming units) which represents a 1.7x fold coverage of the required complexity for full coverage of all the included bacterial and archaeal genomes in all 6 reading frames (based on Clarke-Carbon formula, 1976 (Clarke and Carbon, 1976)).

In order to reduce the number of small peptides (<10 amino acids) encoded by the library an aliquot of the library was digested with BamHI and NotI and separated from the backbone vector by agarose gel electrophoresis. The digested fragment containing DNA encoding the peptides was excised and purified using the QIAquick Gel Extraction Kit (Qiagen #28706) and cloned back into the pcDNALib vector containing an N-terminal V5 tag. The minimal fragment recovery size using this method of DNA purification is 70 base pairs, therefore the minimum peptide size if there is no in-frame stop codon is 12 amino acids. Sequencing analysis confirmed a 33% reduction of sequences encoding small peptides in the pooled plasmid library following this size selection step.

ElectroMAX DH5α-E Competent Cells (Thermo Fisher Scientific #11319019) were transformed by electroporation using the pooled plasmid library. The cells were plated onto 20cm square plates and grown overnight at 37°C. Individual colonies were picked into 2mL of LB Broth +100 μg/mL ampicillin. A final library of 9504 individual clones was created, growing 2mL cultures of individual clones for 24 hr, 37°C, 220 rpm in 96 well deep block plates and recovering mini-prep DNA using the Wizard SV 96 Plasmid DNA Purification System and Vac-Man 96 Manifold (Promega Corp #A2258 & #A2291).

FOXO3a relocalisation assay

Cells were transfected with plasmid DNA or siRNA or treated with compounds and incubated for the indicated time. Cells were then fixed in a final concentration of 3.7% formaldehyde by addition of fixative directly to the medium for 10min, then stained with 4 μg/mL Hoechst 33,342 (Invitrogen). For testing of peptides in the U2OS-GFP-FOXO3a cell line, cells were transfected with plasmid DNA using FuGENE 6 (Promega, E2691) as described for the screen. For testing of peptides in the HEK293T cell line, cells were co-transfected

with a plasmid encoding the test plasmid and a plasmid encoding GFP-FOXO3a using JetPRIME transfection reagent in 96-well plates. At the indicated time point, cells were then fixed and stained as described above.

Compound treatment

Leptomycin B was purchased from NEB. PI-103 and BEZ235 were purchased from Selleckchem. GSK690693 was purchased from Sigma. GSK2334470 was purchased from Stratech Scientific Ltd. LY294002 was purchased from Merck Millipore. All compounds were resuspended in DMSO with the exception of Leptomycin B which was supplied as a solution in ethanol. Compounds were serially diluted in medium using a 2-fold dilution series, DMSO was kept constant with a maximum final concentration of 0.2%. Cells were plated in 96-well plates and treated the following day in triplicate with diluted compounds for 3 hr. After 3 hr, cells were fixed and stained according to the FOXO3a Relocalisation Assay protocol.

siRNA

U2OS-GFP-FOXO3a cells were reversed transfected in triplicate with 25nM siRNA per well in 384-well plates using RNAiMAX transfection reagent (Invitrogen, 13778075). Cells were incubated for 72hr then fixed and stained according to the FOXO3a Relocalisation Assay protocol. The target sequence for all oligos used can be found in Table S5.

Protein interference screen

Transfection

The screen was carried out using a protocol adapted for automation on the FXP and NXP liquid handling robots (Beckman Coulter). U2OS-GFP-FOXO3a cells were reverse transfected with the arrayed peptide plasmid library in triplicate, in 384-well flat bottom plates using FuGENE 6 transfection reagent (Promega, E2691). The plasmid library was diluted to an average concentration of 50 ng/μL prior to transfection. Cells were then incubated undisturbed for 48hr before fixation and staining as detailed above for the FOXO3a Relocalisation Assay. The same automated method was used for hit validation in the U2OS-GFP-FOXO3a cell line.

Image acquisition and analysis

For the screen, images were acquired with a Cellomics Arrayscan VTi (Thermo Scientific) using a 10x objective. Adaptive acquisition was used to ensure that at least 300 cells per well were imaged. Automated image analysis was carried out using the Cellomics Molecular Translocation BioApplication V4. A nuclear mask was defined using the Hoechst channel. Mitotic and apoptotic cells were excluded based on Hoechst intensity and clumps of cells which could not be segmented were also discarded. Nuclear and cytoplasmic regions were then defined in the GFP channel using circ and ring functions derived from the nuclear mask. Average intensities were calculated for each region and the ratio between nuclear and cytoplasmic GFP-FOXO3a was then calculated. A user-defined ratio of 1.2 was selected to identify cells with a high nuclear:cytoplasmic ratio of GFP-FOXO3a. Using this ratio, each cell was classified as having either nuclear GFP-FOXO3a (i.e. a nuclear:cytoplasmic ratio greater than or equal to 1.2) or cytoplasmic GFP-FOXO3a (i.e. a nuclear:cytoplasmic ratio less than 1.2). Results were then expressed as the percentage of cells with nuclear GFP-FOXO3a. This ratio was selected as the optimal threshold for high content screening using the PI3K inhibitor PI-103 as a positive control. Cells were treated with either DMSO or with 1 μM PI-103, a concentration giving a high degree of nuclear translocation of GFP-FOXO3a. Images were acquired and then analyzed using a range of nuclear:cytoplasmic ratios to identify a ratio which reproducibly gave the maximal window between DMSO and PI-103 treated cells.

For all FOXO3a relocalisation assays excluding the primary screen, images were acquired with an ImageXpress Micro Confocal (Molecular Devices) using a 10x or 20x objective. At least four fields and 500 cells per well were imaged. Automated image analysis was carried out using the Translocation Enhanced Application Module (Molecular Devices). A nuclear mask was defined using the Hoechst channel. Nuclear and cytoplasmic regions were then defined in the GFP channel using circ and ring functions derived from the nuclear mask. Median intensities were calculated for each region and the ratio between nuclear and cytoplasmic GFP-FOXO3a was then calculated. A user-defined cut-off ratio of 1.2 was selected to identify cells with a high nuclear:cytoplasmic ratio of GFP-FOXO3a.

Data analysis

Raw data was reported as percentage of cells with high nuclear:cytoplasmic ratio of GFP-FOXO3a per well. Data was normalised to plate median. Median absolute deviation (MAD) was used for hit selection (Chung et al., 2008) as follows. The median of all samples on a plate (plate median) was calculated, then the distance of each individual sample from the plate median was calculated. Finally, the median of these values is calculated to give the MAD. Each sample on the plate was then given a Z score which was calculated as how many MAD the value is from the plate median. For replicate plates the median Z score was taken. A Z score cut-off of >3 was selected to identify primary hits for validation.

SDS-PAGE and western blotting

Cell pellets were lysed in lysis buffer (20mM Tris (pH8), 150mM NaCl, 0.5% NP-40, 1mM EDTA) supplemented with protease inhibitors (cOmplete EDTA-free protease inhibitor cocktail; Roche) and phosphatase inhibitor cocktails 2 and 3 (P5726 and P0044; Sigma). Lysates were heated at 70°C for 10min in NuPAGE LDS sample buffer. Proteins were separated by SDS-PAGE on a Bolt 12% Bis-Tris Plus or a NuPAGE 4–12% Bis-Tris gel (Invitrogen) and transferred to polyvinylidene difluoride (PVDF) membrane in NuPAGE transfer buffer plus 7.5% methanol. The membranes were blocked in Tris-buffered saline plus 0.1% Tween 20 (TBST) plus 5% milk powder then incubated with primary antibodies: pan-14-3–3 antibody (8312S, Cell Signaling Technology), V5 antibody (R96025, Invitrogen),

14-3-3γ (Cell Signaling Technology, 5522), FOXO3a (Cell Signaling Technology 12,829), FLAG (Sigma, F1804), HSP90 (Cell Signaling Technology, 4877S), pan-AKT (Cell Signaling Technology, 2920S), AKT-pSer473 (Cell Signaling Technology, 4060S), FOXO3a-pSer253 (Cell Signaling Technology, 9466S), beta-actin (Sigma Aldrich, A5441), YAP (Cell Signaling Technology, 12395S), p53 (Santa Cruz, sc-126). Membranes were then incubated with horseradish peroxidase (HRP) conjugated secondary antibodies and developed using Amersham ECL Western Blotting Detection Reagents.

V5 immunoprecipitation

Cells were transfected in T75 flasks with plasmids using JetPRIME transfection reagent and harvested at the indicated time point. Immunoprecipitation experiments were carried out using 0.4 to 1.3mg of cell lysate. Where cells were lambda phosphatase treated, each lysate was incubated with 2000 units of lambda protein phosphatase (New England Biolabs, P0753S) for 30min at 37°C prior to incubation with antibody-conjugated beads. Anti-V5 antibody (R96025, Invitrogen) was conjugated to Protein G Dynabeads (10003D, Thermo Fisher Scientific) by incubating 1.6 μg of antibody with 30 μL of beads. Lysates were cleared by incubation with 30 μL unconjugated beads for 1hr. Cleared lysates were then incubated with V5 antibody-conjugated beads for 18hr at 4°C. The beads were washed with lysis buffer then bound proteins were eluted. For samples to be silver stained, proteins were eluted from beads by incubation with 20 μL of 1mg/ml V5 peptide (abcam; ab15829) at 37°C for 10min. For Western blotting, proteins were eluted from beads by denaturation in LDS loading buffer (Invitrogen) at 70°C. Eluted proteins were then resolved by SDS-PAGE.

Silver staining

Following SDS-PAGE the gel was washed in distilled water then fixed and stained using the Pierce Silver Stain Kit (Thermo Fisher Scientific, 24,612). The gel was fixed in 30% ethanol:10% acetic acid solution for 2 × 15min, then incubated in Sensitizer solution for 1min followed by washing in distilled water. The gel was stained for 30min then washed and developed for 2-3min until bands appeared. The reaction was stopped with 5% acetic acid. Sensitizer, stain and developer solutions were supplied as part of the silver stain kit.

Mass spectrometry

Mass spectrometry was carried out by the Cambridge Center for Proteomics. 1D gel bands were transferred into a 96-well PCR plate. The bands were cut into 1mm^2 pieces, destained, reduced (DTT) and alkylated (iodoacetamide) and subjected to enzymatic digestion with trypsin overnight at 37°C. After digestion, the supernatant was pipetted into a sample vial and loaded onto an autosampler for automated LC-MS/MS analysis.

All LC-MS/MS experiments were performed using a Dionex Ultimate 3000 RSLC nanoUPLC (Thermo Fisher Scientific Inc, Waltham, MA, USA) system and either a QExactive or Fusion Lumos Orbitrap mass spectrometer (Thermo Fisher Scientific Inc, Waltham, MA, USA). Separation of peptides was performed by reverse-phase chromatography at a flow rate of 300 nL/min and a Thermo Scientific reverse-phase nano Easy-spray column (Thermo Scientific PepMap C18, 2 μm particle size, 100 Å pore size, 75 μm i.d. x 50 cm length). Peptides were loaded onto a pre-column (Thermo Scientific PepMap 100 C18, 5 μm particle size, 100 Å pore size, 300 μm i.d. x 5 mm length) from the Ultimate 3000 autosampler with 0.1% formic acid for 3 min at a flow rate of 10 μL/min. After this period, the column valve was switched to allow elution of peptides from the pre-column onto the analytical column. Solvent A was water +0.1% formic acid and solvent B was 80% acetonitrile, 20% water +0.1% formic acid. The linear gradient employed was 2–40% B in 30 min.

The LC eluant was sprayed into the mass spectrometer by means of an Easy-Spray source (Thermo Fisher Scientific Inc.). All m/z values of eluting ions were measured in an Orbitrap mass analyzer which was scanned between m/z 380–1500. Data dependent scans were employed to automatically isolate and generate fragment ions by higher energy collisional dissociation in the HCD collision cell and measurement of the resulting fragment ions was performed in the Orbitrap analyser. Singly charged ions and ions with unassigned charge states were excluded from being selected for MS/MS and a dynamic exclusion window of 20 s was employed.

Post-run, the data was processed using Protein Discoverer (version 2.3., Thermo Scientific). Briefly, all MS/MS data were converted to mgf files and the files were then submitted to the Mascot search algorithm (Matrix Science, London UK) and searched against the UniProt human database (93,609 sequences; 37041084 residues) and a common contaminant sequences (123 sequences; 40,594 residues). Variable modifications of oxidation (M) and deamidation (NQ) and a fixed modification of carbamidomethyl were applied. The peptide and fragment mass tolerances were set to 20 ppm and 0.1 Da, respectively. A significance threshold value of $p < 0.05$ and a peptide cut-off score of 20 were also applied.

Yeast 2 hybrid screening

The ULTImate Y2H screen was performed by Hybrigenics Services (Paris, France; https://www.hybrigenics-services.com/). Full length 9J10 peptide was cloned into the pB27 plasmid as a fusion with LexA (N-LexA-bait-C). The bait was used to screen a HeLa cell cDNA library. A total of 80.3 million interactions were analyzed.

FLAG immunoprecipitation

The 14-3-3ε coding sequence was isolated by PCR from a vector containing a cDNA clone for YWHAE (Cusabio, CL026287HU Gene ID 7531 Accession Number BC000179) using forward primer ACGTGAATTCCATGGATGATCGAGAGGATCTGG and reverse primer ACGTTCTAGATCACTGATTTTCGTCTTCCACGTCCTGC. The PCR product was digested with EcoRI and XbaI and cloned into these sites in the expression vector p3XFLAG-CMV-10 (Sigma, E6758). Cells were co-transfected in T75 flasks with a plasmid encoding

FLAG-tagged 14-3-3ε plus empty vector, 9J10^{WT} or 9J10^{AMut} using JetPRIME transfection reagent, and harvested at the indicated time point. Anti-FLAG M2 Affininty Gel (Sigma, A2220) was washed in TBS then 40 μL of bead slurry was incubated with 1.2mg of cell lysate for 18hr at 4°C. The beads were washed then bound proteins were eluted by denaturing in LDS loading buffer (Invitrogen) at 70°C. Eluted proteins were then resolved by SDS-PAGE.

Immunofluorescence for endogenous FOXO3a

HEK293T cells were plated in 96-well plates and treated as indicated. Cells were then fixed in a final concentration of 3.7% formaldehyde by addition of fixative directly to the medium for 10min, then permeabilised for 10 min in PBS containing 0.1% Triton X-100. Cells were then incubated with anti-FOXO3a antibody (Cell Signaling Technology 12,829), diluted 1:1000 in PBS +1% bovine serum albumin (BSA) for 2hrs. Cells were washed in PBS + 1% BSA then incubated with Alexa Fluor 488 conjugated goat anti-rabbit IgG (A11034, Thermo Fisher Scientific) and 4 μg/ml Hoechst 33,342 (Invitrogen) for 1hr. Images were acquired on the ImageXpress Micro Confocal and analyzed as described above for screening.

9J10 mutant plasmid constructs

Vector pcDNALib-9J10 was used as a template for conventional PCR using the primers below to mutate serine 117 in the V5-9J10 peptide to alanine (A) and retain the HindIII and NotI restriction enzyme recognition sites to enable cloning into either pcDNALib or pcDNA3.1(+) (Thermo Fisher Scientific #V79020)

FWD Primer: CCGGACTCTAGCAAGCTTACCATGGGTAAGCCTATCCC Tm80.1°C.

REV “A” Primer: CGAGCGGCCGCTCAATTAATTAGCATTGCG Tm80.6°C.

Live cell confluency measurements

HEK293T, MCF7 and BT549 cells were plated into Eppendorf 96 well cell culture plates at 20 000, 10 000 and 5 000 cells per well respectively in standard growth media (see above). The following day HEK293T cells were transfected using JetPRIME reagent (Polyplus, 114-07), MCF7 and BT549 cells were transfected using Fugene 6 (Promega, E2691) with plasmid DNA coding for either Vector, 9J10^{WT} or 9J10^{AMut} peptides. Four hours post-transfection media was changed for MCF7 and BT549 cells and the plates placed into an Incucyte Zoom Live-Cell Analysis System (Essen Bioscience). Cell confluency was measured with the Basic Analyzer setting, user defined parameters and the 10X objective. Four images per well were captured at 2 hourly intervals for 48 hours. Where siRNA was included, cells were reverse transfected with 40nM siRNA using RNAiMAX transfection reagent (Invitrogen, 13778075), then forward transfected 24 hours later with plasmid DNA as described above. The target sequence for all oligos used can be found in Table S5.

Forkhead luciferase reporter assay

The 6 x Forkhead Response Element (FHRE) (Furuyama et al., 2000) luciferase reporter was constructed by hybridising the following oligos:

6 x FHRE Top CTAGAGTTGTTTACATAGTTGTTTACTAGTAGTTGTTTACATAGTTGTTTACTTATAGTTGTTTACATAGTTGTTTACTA

6 x FHRE Bottom

AGCTTAGTAAACAACTATGTAAACAACTATAAGTAAACAACTATGTAAACAACTACTAGTAAACAACTATGTAAACAACTCTAGGTAC

The hybridised oligos were cloned into pGL4.20 (Promega Corp #E6751) via the KpnI and HindIII restriction enzyme digestion sites in the vector’s multiple cloning site (MCS).

The reporter vector was transfected into HEK293T cells using JetPRIME reagent in 96 well plates along with pRL-TK (Promega Corp #E2241) for normalisation, and the plasmid being tested. 48 hours post transfection the cells were assayed using the Dual-Glo Luciferase Assay System (Promega Corp #E2940). Results are shown as fold change compared to vector control.

RNA sequencing: sample preparation

Transfection

HEK293T cells were transfected in T75 flasks with plasmid DNA encoding Vector, 9J10^{WT}, 9J10^{AMut}, GFP-FOXO3a-AAA or GFP, using JetRPIME transfection reagent. Cells were harvested 24 hr post-transfection. Five independent transfections on different days and using cells of different passage number were carried out for each condition.

RNA extraction and quantification

Total RNA was isolated using the Qiagen RNeasy Mini Kit (#74104) complete with DNase digestion (Qiagen #79254), according to manufacturers instructions. RNA was submitted to Novogene for transcriptome sequencing. RNA degradation and contamination was monitored on 1% agarose gels. RNA purity was checked using the NanoPhotometer spectrophotometer (IMPLEN, CA, USA), and RNA integrity and quantitation were assessed using the RNA Nano 6000 Assay Kit of the Bioanalyzer 2100 system (Agilent Technologies, CA, USA).

Library preparation

A total amount of 1 μg RNA per sample was used as input material for the RNA sample preparations. Sequencing libraries were generated using NEBNext UltraTM RNA Library Prep Kit for Illumina (NEB, USA) following manufacturer’s recommendations and index codes were added to attribute sequences to each sample. Briefly, mRNA was purified from total RNA using poly-T oligo-attached

magnetic beads. Fragmentation was carried out using divalent cations under elevated temperature in NEBNext First Strand Synthesis Reaction Buffer (5X). First strand cDNA was synthesized using random hexamer primer and M-MuLV Reverse Transcriptase (RNase H-). Second strand cDNA synthesis was subsequently performed using DNA Polymerase I and RNase H. Remaining overhangs were converted into blunt ends via exonuclease/polymerase activities. After adenylation of 3′ ends of DNA fragments, NEBNext Adaptor with hairpin loop structure were ligated to prepare for hybridization. In order to select cDNA fragments of preferentially 150–200 bp in length, the library fragments were purified with AMPure XP system (Beckman Coulter, Beverly, USA). Then 3 μL USER Enzyme (NEB, USA) was used with size-selected, adaptor-ligated cDNA at 37°C for 15 min followed by 5 min at 95°C before PCR. Then PCR was performed with Phusion High-Fidelity DNA polymerase, Universal PCR primers and Index (X) Primer. At last, PCR products were purified (AMPure XP system) and library quality was assessed on the Agilent Bioanalyzer 2100 system.

Clustering and sequencing

The clustering of the index-coded samples was performed on a cBot Cluster Generation System using PE Cluster Kit cBot-HS (Illumina) according to the manufacturer's instructions. After cluster generation, the library preparations were sequenced on an Illumina platform and paired-end reads were generated.

RNA sequencing: data analysis

Quality control

Raw data (raw reads) of FASTQ format were firstly processed through fastp. Clean data (clean reads) were obtained by removing reads containing adapter and poly-N sequences and reads with low quality from raw data. At the same time, Q20, Q30 and GC content of the clean data were calculated. All the downstream analyses were based on the clean data with high quality.

Mapping to reference genome

Reference genome and gene model annotation files were downloaded from genome website browser (NCBI/UCSC/Ensembl) directly. Paired-end clean reads were aligned to the reference genome using the Spliced Transcripts Alignment to a Reference (STAR) software, which is based on a previously undescribed RNA-seq alignment algorithm that uses sequential maximum mappable seed search in uncompressed suffix arrays followed by seed clustering and stitching procedure.

Quantification of gene expression level

HTSeq v0.6.1 was used to count the read numbers mapped to each gene, and then FPKM of each gene was calculated based on the length of the gene and reads count mapped to this gene. FPKM, Reads Per Kilobase of exon model per Million mapped reads, considers the effect of sequencing depth and gene length for the reads count at the same time, and is currently the most commonly used method for estimating gene expression levels (Mortazavi et al., 2008).

Differential expression analysis

Differential expression analysis between two conditions/groups (with biological replicates) was performed using the DESeq2 R package (2_1.6.3). DESeq2 provide statistical routines for determining differential expression in digital gene expression data using a model based on the negative binomial distribution. The resulting p values were adjusted using the Benjamini and Hochberg's approach for controlling the False Discovery Rate (FDR). Genes with an adjusted p value of <0.05 found by DESeq2 were assigned as differentially expressed.

Transcription factor motif enrichment analysis

oPOSSUM 3.0 (http://opossum.cisreg.ca/oPOSSUM3/) (Ho Sui et al., 2007; Kwon et al., 2012) was used to identify enriched transcription factor motifs among differentially regulated gene sets. The tool was run with the following parameters: all oPOSSUM database genes were used as background, a conservation score of 0.40 and a matrix score threshold of 85% were applied, a search region of 2000 bp upstream and downstream of the transcription start site was used. Only motifs with a Z score >10 and a Fisher score >7 were considered enriched.

Protein expression and purification

For crystallography

A synthetic gene construct (GeneArt, Thermo Fisher) encoding full length human 14-3-3ε, codon-optimised for expression in E. coli was PCR amplified to give a C-terminally truncated 14-3-3ε construct (amino acids 1–232) and inserted into pET28a vector (Novagen 69,864-3) containing an N-terminal 6xHis tag with a thrombin cleavage site. The resulting plasmid was then expressed in E.coli BL21 DE3 cells. Cultures were grown in Luria Broth media at 37°C and when the OD600 reached 0.6, the cells were induced with 1mM isopropyl β-D-1-thiogalactopyranoside (IPTG) and continued growth at 37°C for 4 hr. The cells were harvested by centrifugation and re-suspended in buffer containing 50 mM HEPES pH 7.5, 300 mM NaCl, 1 mM DTT, 0.1 mM PMSF and 1 protease inhibitor tablet and lysed by sonication. The supernatant was passed through a Ni-NTA IMAC column, followed by washes with 50 mM HEPES pH 7.5, 300 mM NaCl, 1 mM DTT and 0–20 mM Imidazole. The protein was eluted using 50 mM HEPES pH 7.5, 300 mM NaCl, 1 mM DTT and 500 mM Imidazole. The affinity purified protein was injected into S75 Superdex size exclusion column.

For FP and biophysical assays (MST, ITC and STD-NMR)

A synthetic gene construct encoding full length human 14-3-3ε, codon-optimised for expression in E. coli (GeneArt, Thermo Fisher) was cloned into the pET28a vector using the BamHI and XhoI restriction enzyme sites. 6x His-14-3-3ε expression was induced in BL21(DE3) strain at 0.6 OD600 with 1 mM isopropyl β-D-1-thiogalactopyranoside (IPTG) and the culture was grown for 4h at 37°C. Cells were harvested and the pellet was suspended in ice-cold lysis buffer (10mL/gm of cell pellet; 50mM HEPES [pH 7.5],

300mM NaCl, 10mM imidazole, 0.1mM PMSF, 1mM DTT, and 1 protease inhibitor tablet (Roche)). Cells were lysed by sonication on ice and centrifuged at 20,000 rev min-1 for 45min at 4°C to remove cell debris. The supernatant was applied onto a HisTrap HP column (GE Healthcare) pre-equilibrated with buffer (50mM HEPES [pH 7.5], 300mM NaCl, 0.5mM DTT, and 25mM Imidazole). The column was washed with the same buffer until all unbound proteins were removed. The protein of interest was eluted using a linear gradient of up to 100% elution buffer (50mM HEPES [pH 7.5], 300mM NaCl, 0.5mM DTT, and 250mM Imidazole). Protein purity was visualized by running SDS-PAGE. Fractions of sufficient purity were pooled and concentrated to 2mL using a 10 kDa cut-off Centricon centrifugal filter devices (Millipore). The concentrated protein was further purified using HiLoad 16/600Superdex-75 prep-grade gel-filtration column (GE Healthcare) pre-equilibrated with 10mM HEPES, 150mM NaCl and 0.5mM DTT.

Isothermal titration calorimetry

14-3-3ε protein was dialyzed in dialysis buffer containing 50mM HEPES, 150mM NaCl and 0.5mM DTT. TAMRA-9J10 peptide, 5-TAMRA- LNRTPGRRRN(p-Serine)N-acid (Sigma-Aldrich), was dissolved in water and then finally diluted in dialysis buffer. ITC experiments were performed using MicroCal ITC 200 instrument. All experiments were carried out at 25°C on high feedback mode with stirring speed 800rpm and filter period time 5 s. 270 μL volume of 24 μM 14-3-3ε was titrated with 20 injections of 360 μM TAMRA labeled 9J10 with each 2 μL injections with 120 s intervals between each injections. Control experiment was conducted with identical buffer condition and protein in the cell was replaced by buffer and this control experimental result was used to subtract to account for heat of dilution. The data was analyzed by origin 7 software and the results were fit by using one set of sites model.

Crystallization and structure determination

The purified, 15 mg/ml C-terminal truncated 14-3-3ε protein was incubated with a TAMRA-9J10 peptide, 5-TAMRA- LNRTPGRRRN (p-Serine)N-acid (Sigma-Aldrich), at a ratio of 1:2 (0.5 mM: 1 mM) supplemented with 0.05 mM EDTA and 1 mM $MgCl_2$. The incubated mixture was screened using Hampton and Jena Bioscience (Basic and Classic) condition kits using sitting drop at 16°C. Diffracting crystals were obtained in condition containing 28% PEG 400, 0.1M Hepes pH 7.5 and 0.2M Calcium chloride. Diffraction data was collected to 3.16 Å at 100 K at the homesource Rigaku FR-X rotating anode X-ray diffractometer with R-Axis-IV++ detector. The diffraction images were processed and integrated using iMosflm (Battye et al., 2011), later scaled using Scala program (Evans, 2006) in CCP4 suite (Winn et al., 2011). Initial structure solution of the protein was obtained by MR method using Phaser MR module (McCoy et al., 2007), with 2BR9 (Yang et al., 2006) monomer as the starting model. Further model building was done in Coot (Emsley and Cowtan, 2004) followed by refinement in Refmac5 program (Murshudov et al., 2011). Atomic coordinates and structure factors have been deposited to PDB under the accession code PDB: 7C8E, shown in Table S4.

Fluorescence polarisation (FP) assay

All FP Assays were carried out in black 384 well Non-Binding Surface (NBS) plates (Corning) in 10mM HEPES, 150mM NaCl +0.05% Tween. Assay plates were incubated at room temperature for 20 min before measuring FP values using the BMG PHERAstar plate reader with the 540/590/590nm FP filter module set to read wells containing 10nM TAMRA-p9J10Min peptide (5-TAMRA-GRRRN(p-Serine)N-acid (Sigma-Aldrich)), only at 35mP. His-14-3-3ε was titrated against 10nM TAMRA-p9J10Min peptide and from 3 independent experiments a Kd of 1.47(+/−0.71)μM was calculated for the interaction (Figure S6A). To determine if the interaction could be inhibited an unlabelled 9J10 peptide, LNRTPGRRRN(p-Serine)N-acid (Sigma-Aldrich) was titrated against TAMRA-p9J10Min peptide (10nM) bound to His-14-3-3ε (1.5μM). The peptide disrupted binding giving a mean IC_{50} of 9.8(+/−1.2)μM from 3 independent experiments (Figure S6B).

Then using the same conditions as above, 1.5 μM His-14-3-3ε and 10 nM TAMRA-p9J10Min peptide, we calculated that the Z-factor for the assay was 0.595 from 80 wells of 10nM TAMRA-p9J10Min only (negatives) and 80 wells of 10 nM TAMRA-p9J10Min plus 1.5 μM His-14-3-3ε (positives), thereby enabling a high throughput screening (HTS) campaign to be initialised (Figure S6C).

Mean of negatives (μn) = 37.1 Standard Deviation (σn) = 4.1.

Mean of positives (μp) = 151.9 Standard Deviation (σp) = 11.4

$$Z - factor = 1 - \frac{3(\sigma_p + \sigma_n)}{|\mu_p - \mu_n|}$$

For FP HTS the assay components were as above, compounds were screened at an approximate final concentration of 125 μM and final DMSO concentration of 1%. Appropriate volumes of TAMRA-p9J10Min peptide and His-14-3-3ε at 3X final concentration were made up in assay buffer respectively and placed in reservoirs on the Beckman Coulter Biomek FXp Automated Workstation deck in the positions indicated by the scheduling software. Compound dilution plates, black 384 well NBS assay plates and tip racks were loaded into the Cytomat as indicated by the scheduling software. The appropriate Fluorescence Polarisation program was initiated on the BMG PheraStar plate reader and was manually set using an assay plate and 10 nM TAMRA-p9J10Min to an FP value of 35mP. Test compounds were diluted to 3X final concentration in the dilution plates. 10 μL of diluted compound was added into each well of the assay plate followed by 10μL of His-14-3-3ε and 10μL of TAMRA-p9J10Min. Plates were incubated at room temperature for 20 min on the robot's deck or in the Cytomat before the Fluorescence Polarisation was read using the BMG PheraStar plate reader. Test compounds were assayed in triplicate. Mean % inhibition normalised to DMSO control (1%) was calculated for each compound. Compounds with a mean inhibition greater than 30% were deemed hits. Hit compounds were re-purchased where

available and were tested to see if they could competitively inhibit the binding of TAMRA-p9J10Min to His-14-3-3ε in a dose dependant manner. Compounds were titrated 2-fold from a top concentration of 200 μM giving a maximum final concentration of DMSO in the assay of 0.4%. DMSO controls were run alongside all experimental compounds and % inhibition normalised to these controls. All other experimental conditions were as described above.

Microscale Thermophoresis (MST)

The 14-3-3ε protein was labeled with NT-647-NHS fluorescent dye using the Monolith Protein Labeling Kit (Cat# MO-L011, NanoTemper Technologies). Assays were carried out in 10 mM Hepes, 150 mM NaCl and 0.01% Tween 20. For the direct binding assay, 10 μL of labeled protein at a final concentration of 20 nM was mixed with 10 μL of cognate peptide. For the competitive assay, 10 μL of labeled protein at a final concentration of 20 nM was mixed with 1 μM of cognate peptide at the EC80 concentration determined by prior titration using a 16-point serial dilution by direct-binding MST. For both assays, samples were prepared as above, incubated for 20 min and centrifuged at 15,000 rpm at 4°C for 5 min and 10 μL of the supernatant was loaded into premium glass capillaries (NanoTemper Technologies). MST analysis was performed at MST power of 60% and LED power of 50%, at 25°C temperature using a Monolith NT.115 (NanoTemper Technologies). An initial ''CapillaryScan'' was performed to scan for fluorescence across the length of the capillary tray to determine the exact position of each capillary before the MST measurement was started. Test compound in 2% final DMSO concentration was assayed at 16 different concentrations by serial dilution, and data were analyzed using NanoTemper analysis software with ''T-jump + Thermophoresis'' settings. The change in thermophoresis between different experimental conditions was expressed as the change in the normalized fluorescence (ΔF_{norm}), which is defined as F_{hot}/F_{cold} (F-values correspond to average fluorescence values between defined areas in the curve under steadystate conditions under control (F_{cold}) or experimental (F_{hot}) conditions). Titration of the non-fluorescent ligand causes a gradual change in thermophoresis, which is plotted as Fraction bound to yield a binding curve, which was then fitted using Kd model and derived binding constants Kd and inhibition constant IC50 using Graphpad Prism software.

STD-NMR

1H NMR and STD spectra were recorded with a Bruker Avance-III HD 600 MHz at 298 K. Bruker TopSpin software was used to acquire and process the NMR data. NMR samples were prepared in D2O, with 100 μM 9J10 peptide (LNRTPGRRRN(p-Serine)N-acid) (Sigma-Aldrich), and 10 μM 14-3-3ε protein. For STD NMR measurements standard Bruker pulse sequence stddiffesgp.3 was used, the on-resonance frequency was set to be 0 ppm, and the off-resonance frequency at −40 ppm. The number of scans was typically 64, both the saturation pulses and relaxation delay were set to be 5 s and the power of the shaped pulse for saturation was set at 40 dB. Experiments with the varying concentrations of CU7218 were performed by mixing appropriate volume of the compound in DMSO and the final DMSO concentration was adjusted to 2.0% by using d6-DMSO.

QUANTIFICATION AND STATISTICAL ANALYSIS

Statistical analysis and n number is indicated throughout in the figure legends. Data is represented as mean +/− standard deviation unless otherwise stated in the figure legend. Kd and IC_{50} values were calculated using Graphpad Prism software.

Cell Chemical Biology, Volume *28*

Supplemental information

Target identification for small-molecule discovery in the FOXO3a tumor-suppressor pathway using a biodiverse peptide library

Amy Emery, Bryn S. Hardwick, Alex T. Crooks, Nadia Milech, Paul M. Watt, Chandan Mithra, Vikrant Kumar, Saranya Giridharan, Gayathri Sadasivam, Subashini Mathivanan, Sneha Sudhakar, Sneha Bairy, Kavitha Bharatham, Manjunath A. Hurakadli, Thazhe K. Prasad, Neelagandan Kamariah, Markus Muellner, Miguel Coelho, Christopher J. Torrance, Grahame J. McKenzie, and Ashok R. Venkitaraman

SUPPLEMENTAL INFORMATION

FIGURE LEGENDS

Supplementary Figure S1: Perturbation of the AKT and nuclear export pathways leads to an increase in nuclear localisation of GFP-FOXO3a in a U2OS reporter cell line (related to Figure 1) (A) Quantitation of nuclear translocation in U2OS-GFP-FOXO3a cells in response to small molecule inhibitors. Compounds underwent a two-fold serial dilution from a top concentration, on the left for each compound, of 1µM for BEZ235, GSK2334470, GSK69093 and PI-103, 20µM for LY294002 or 2nM for Leptomycin B. Grey bars are DMSO only. BEZ235: a dual phosphoinositide 3-kinase (PI3K)-mammalian target of rapamycin (mTOR) inhibitor. GSK2334470: PDPK1 inhibitor. GSK69063: pan-Akt kinase inhibitor. PI-103: ATP-competitive inhibitor of DNA-PK, PI 3-kinase (Class IA), and FRAP (mTOR) complex 1 and 2. LY294002: PI3K inhibitor. Leptomycin B: inhibitor of Exportin 1. (B) Quantitation of nuclear translocation in U2OS-GFP-FOXO3a cells following siRNA-mediated knockdown of selected AKT and nuclear export pathway components. Single siRNAs were used excepting hatched bars which indicate that a pool of four siRNAs was used. Grey bars are negative or non-targeting controls. Data represent the mean of three independent experiments +/- SD.

Supplementary Figure S2: Validation of the interaction between peptide 9J10 and 14-3-3 (related to Figure 2). (A) Western blotting for 14-3-3 in protein eluates following immunoprecipitation with V5 antibody from U2OS-GFP-FOXO3a cells transfected with the indicated peptides for 48hrs. These samples are the same as those shown in **Figure 2A**. (B) Western blotting for 14-3-3 following V5 immunoprecipitation with additional high stringency wash steps (400mM LiCl) prior to elution. (C) Amino acid sequence of peptide 9J10. (D) Western blotting for 14-3-3 following V5 immunoprecipitation in a panel of cell lines transfected with 9J10 or vector only. Asterisk marks the antibody light chain. With the exception of (A), all Western blots are representative of at least two independent replicates.

Supplementary Figure S3: Perturbation of the AKT/PI3K pathway with small molecule inhibitors in HEK293T cells leads to nuclear translocation of FOXO3a and changes in

pathway specific phosphorylation (related to Figure 2). (A) Representative images of HEK293T cells showing FOXO3a localisation after 3hr treatment with small molecule inhibitors. PI3Ki=1.25µM PI-103, AKTi=1µM GSK690693. The nuclear area is marked by an orange dotted line. Scale bar = 25µm. (B) Representative Western blots of cell lysates from HEK293T cells treated with small molecule inhibitors as in (A). Phosphorylation of AKT at Ser473 is dependent on PI3K activity. Phosphorylation of FOXO3a at Ser253 is carried out by AKT. Independent blots using the same samples were probed for the indicated targets. For each blot, HSP90 expression is shown on the right. (C) Quantitation of Western blots. Intensities were normalised to HSP90 intensities from the same blots then expressed relative to DMSO for each protein. Bars indicate the mean of three independent experiments +/- SD

Supplementary Figure S4: Expression of 9J10^{WT} but not 9J10^{AMut} modulates transcription and impairs cell growth (related to Figure 4) (A) List of over-represented transcription factors for genes regulated by 9J10 alone generated by promoter motif analysis using oPOSSUM-3. Transcription factors with a Z-score >10 and a Fisher score >7 were considered significant. (B) Cell confluency measurements from live cell imaging of MCF7 and (C) BT-549 cells transfected with Vector, 9J10^{WT} or 9J10^{AMut} at the final 48hr timepoint. Bars represent the average of three independent experiments +/- SD. (D) A representative experiment for MCF7 and (E) BT549 cells showing changes in cell confluency over time in cells transfected as in (B) and (C). Data represent the average of four fields +/- SD.

Supplementary Figure S5: Binding by ITC and co-crystal structure of 9J10 and 14-3-3ε (related to Figure 5). (A) Isothermal titration calorimetry demonstrating binding between a minimised 9J10 peptide and 14-3-3ε. A representative experiment is shown from two independent experiments. (B) The 2Fo-Fc map contour at 1 σ level around the 9J10 peptide, from the co-crystal structure. The map clearly shows the 9J10 peptide region. (C) Table showing interacting amino acids between 14-3-3ε protein and 9J10 peptide. (D) The Arg8 at pS-3 position of 9J10 (blue stick) shows significant conformational difference compared to that of Arg at the same position of the canonical phosphopeptides interacting with 14-3-3ε

(PDB: 2BR9 (yellow)); PDB: 6EIH-(orange)). (E) Table showing arginine pairing geometry from co-crystal structure.

Supplementary Figure S6: Identification and validation of compound CU7218 (related to Figure 6). (A) Optimisation of fluorescence polarisation assay. Direct binding of 14-3-3ε to TAMRA-labelled 9J10Min phosphopeptide. Kd value was calculated by titrating 14-3-3ε protein in the presence of 10nM TAMRA-p9J10Min. Plot represents mean of three independent experiments, +/- SD. (B) Competitive inhibition of TAMRA-p9J10Min binding to 14-3-3ε by an unlabelled 9J10 derived peptide measured by FP. Unlabelled 9J10 was titrated in the presence of 10nM TAMRA-p9J10Min. Plot represents mean of three independent experiments, +/- SD. (C) Graph showing Z' calculation for assay quality. Kd and IC_{50} values are the mean of three independent experiments, +/- SD.

Supplementary Figure S1: Perturbation of the AKT and nuclear export pathways leads to an increase in nuclear localisation of GFP-FOXO3a in a U2OS reporter cell line.

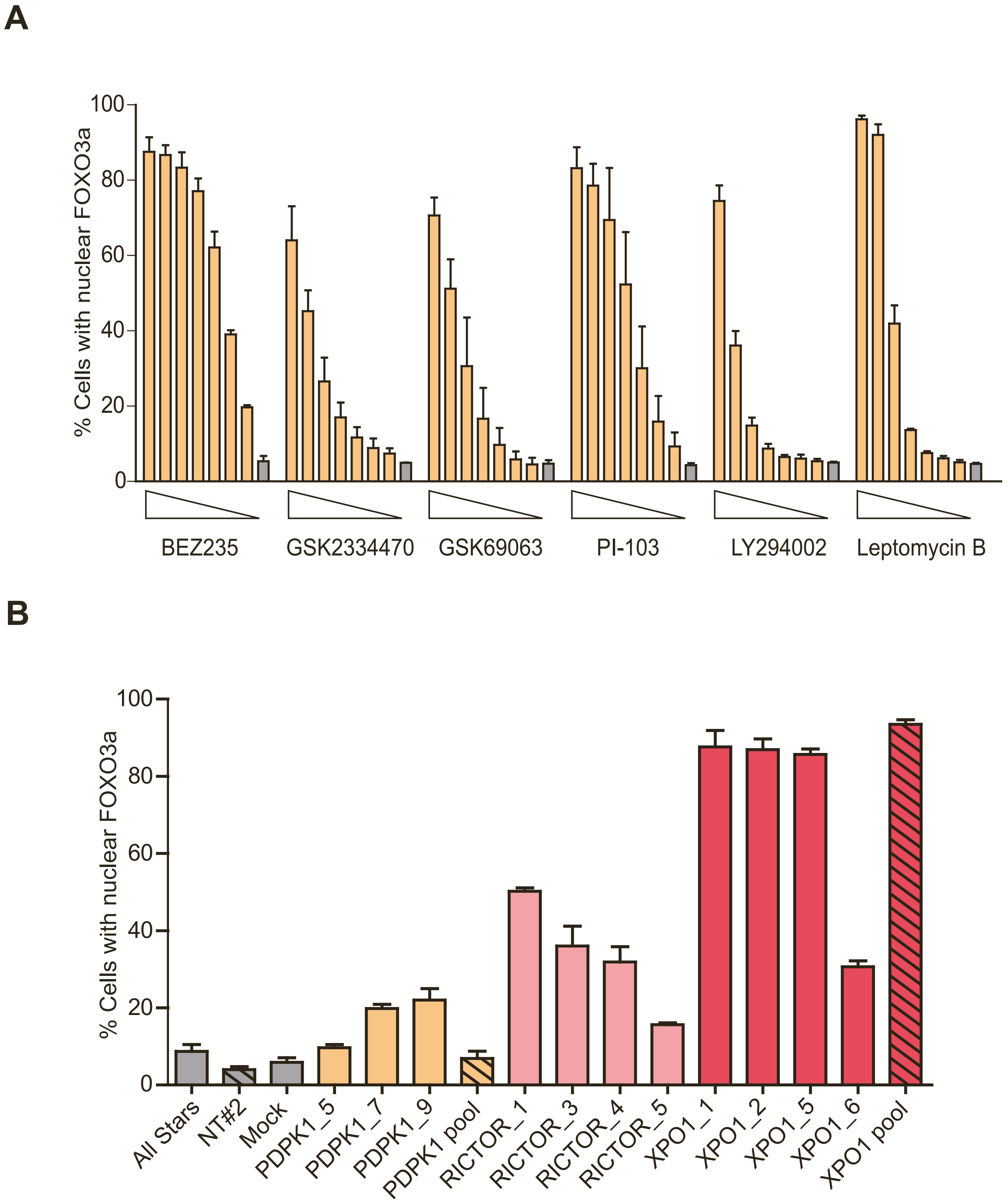

Supplementary Figure S2: Validation of the interaction between peptide 9J10 and 14-3-3.

A

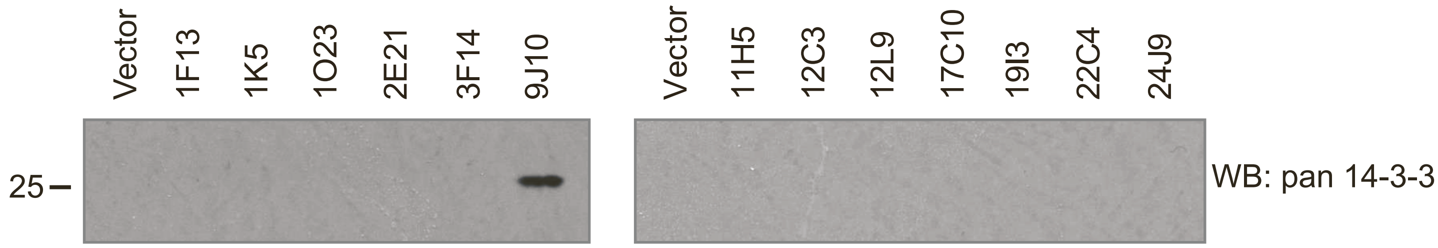

B

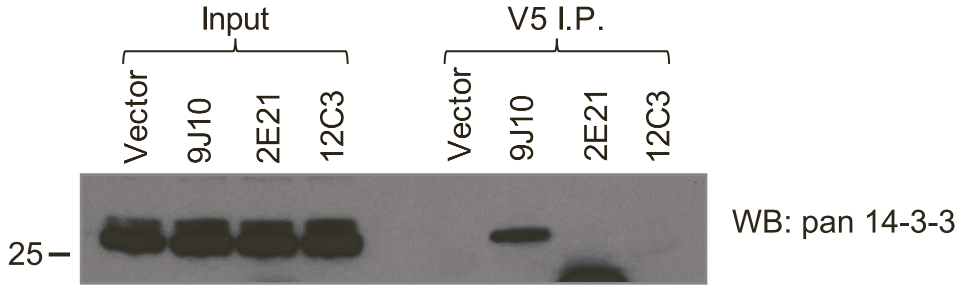

C

PTTSDNLLLSPELRDGARGAKRLCPEIGGDEAWRLLTEDAVAEQGEPVS
SVYLLRDPLRPSGRALGPVADQRGPPLAATAPIPLNRTPGRRRNSN

D

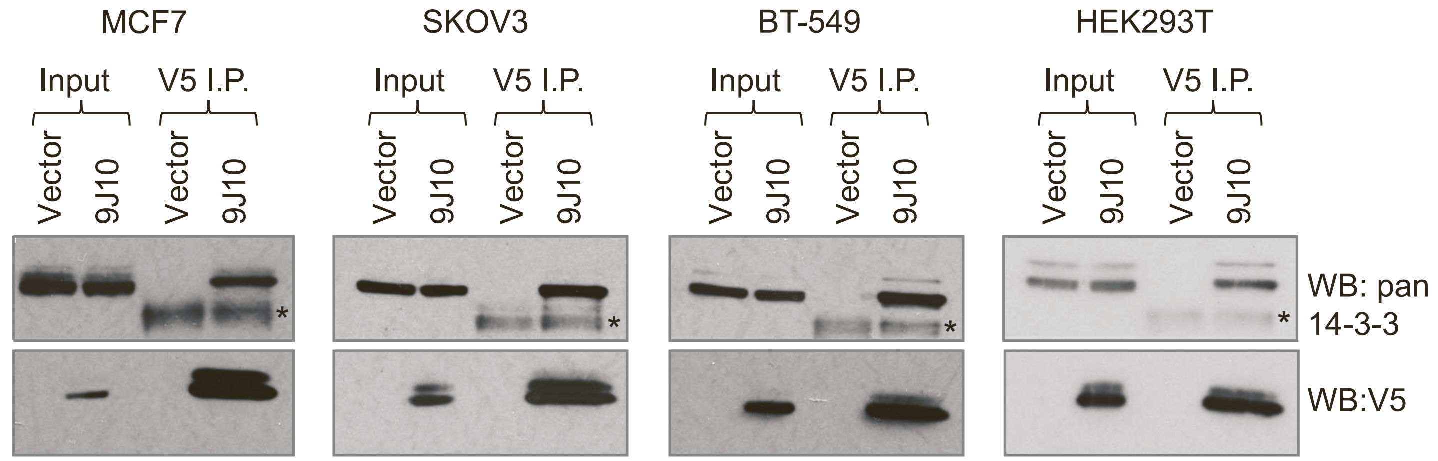

Supplementary Figure S3: Perturbation of the AKT/PI3K pathway with small molecule inhibitors in HEK293T cells leads to nuclear translocation of FOXO3a and changes in pathway specific phosphorylation.

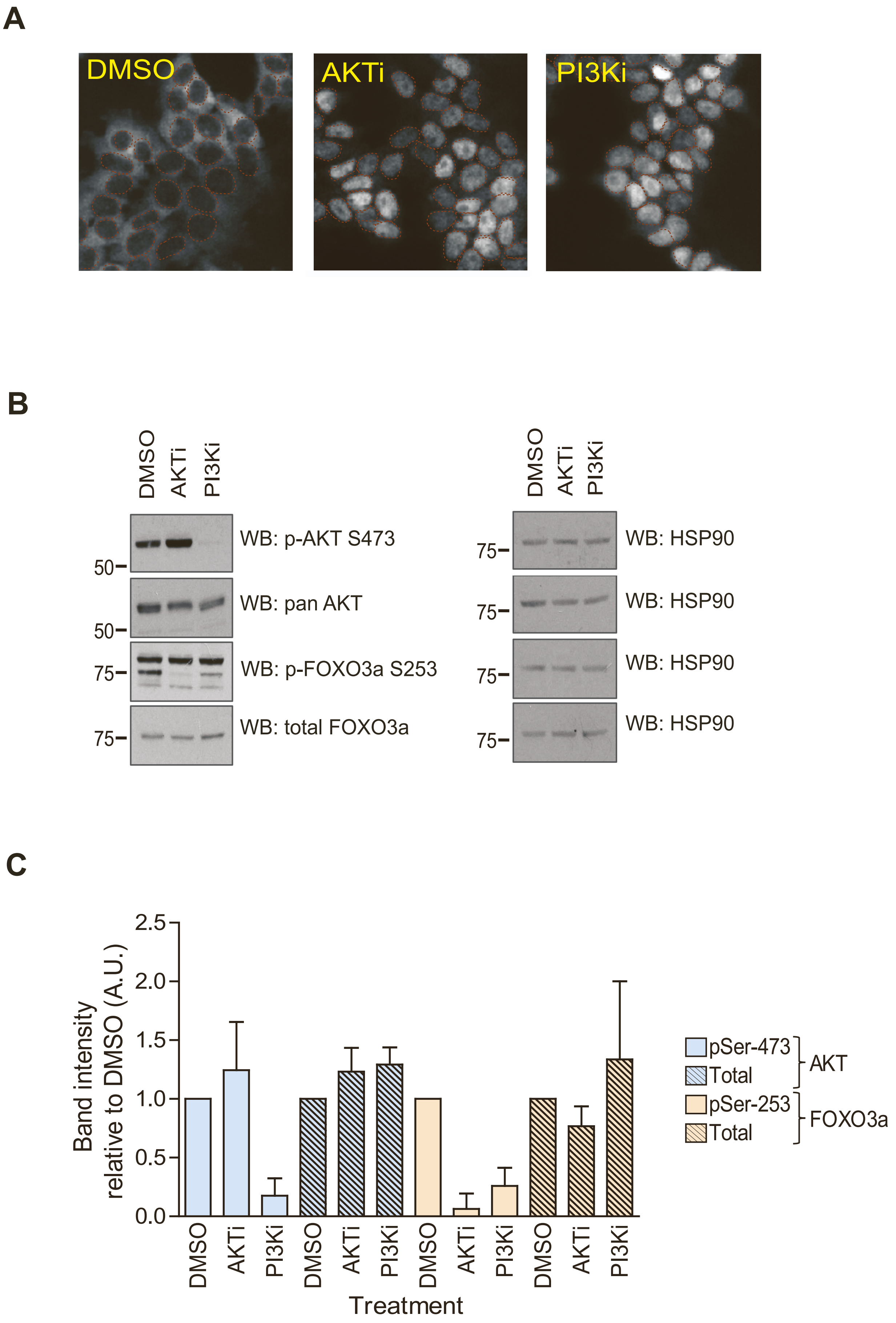

Supplementary Figure S4: Expression of 9J10^{WT} but not 9J10^{AMut} modulates transcription and impairs cell growth.

A

Transcription factor	Z-score	Fisher Score
EWSR1-FLI1	17.1	15.7
FOXI1	16.6	41.7
SRY	15.6	43.7
HOXA5	14.1	70.1
NFYA	13.8	69.7
ARID3A	13.5	47.7
TBP	13.1	37.3
Foxd3	12.4	38.5
Pdx1	12.3	46.2
Nkx2-5	11.7	42.7
Gfi	10.5	61.7
Foxq1	10.4	28.7

B

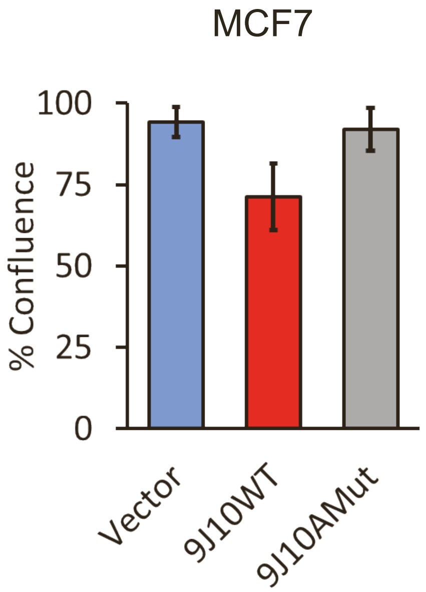

C

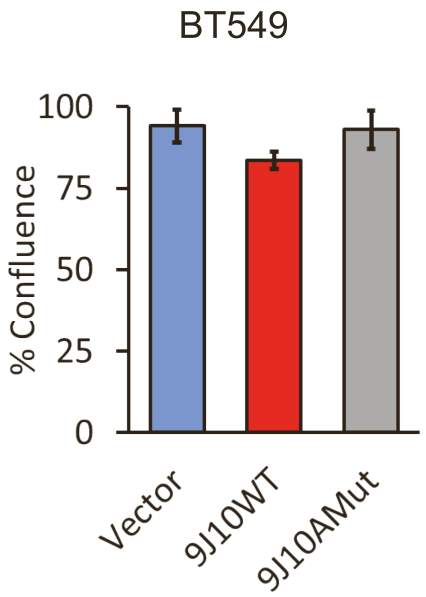

D

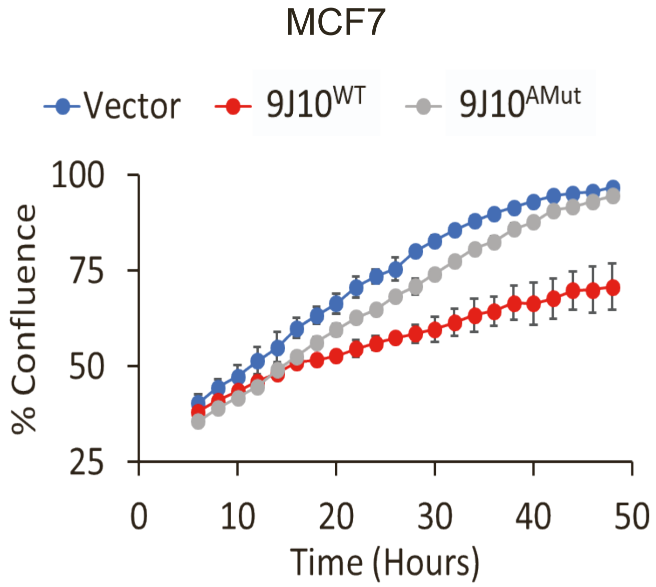

E

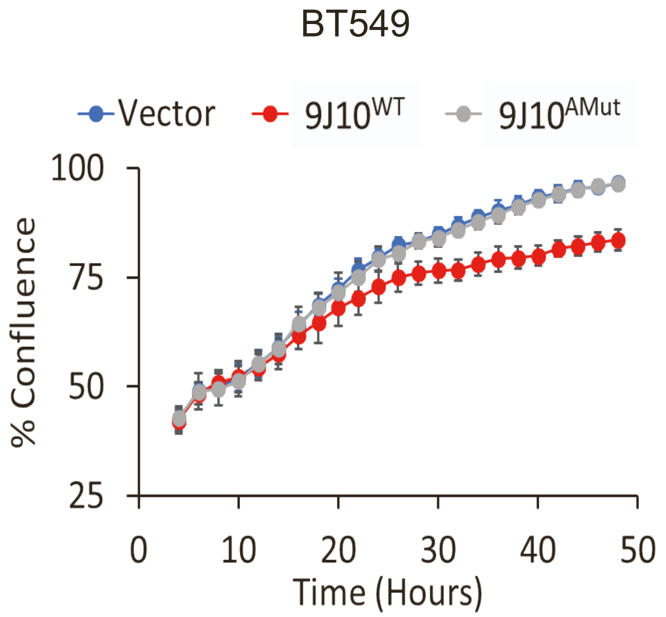

Supplementary Figure S5: Binding by ITC and co-crystal structure of 9J10 and 14-3-3ε.

A

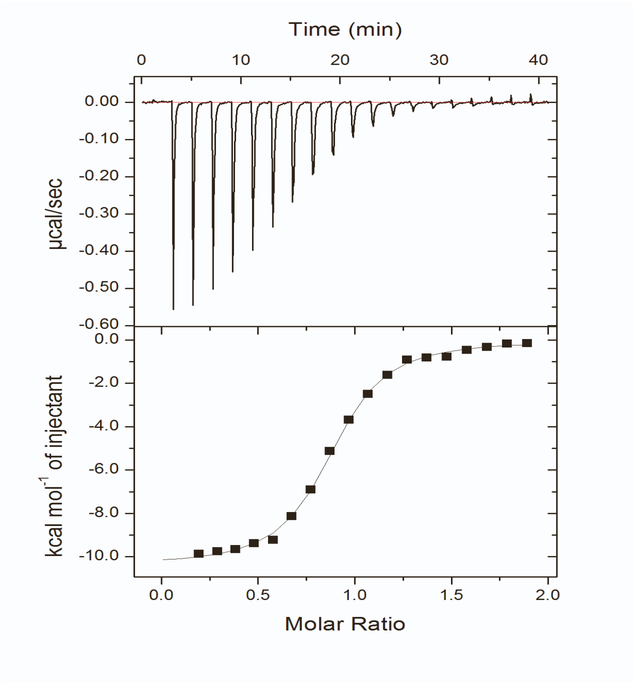

B

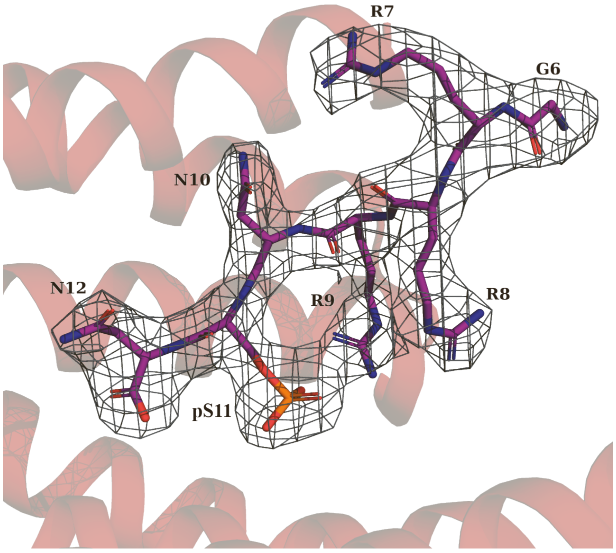

C

Interactions	14-3-3 (atom)	9J10 (atom)	Distance (Å)
H-Bonds	Asn 227 (OD1)	Asn 10 (N)	2.8
	Asn 227 (OD1)	Asn 10 (ND2)	3.3
	Asn 227 (ND2)	Asn 10 (O)	3.2
	Leu 223 (O)	Asn 10 (ND2)	3.1
	Asn 176 (OD1)	Asn 12 (N)	3.0
	Asn 176 (ND2)	Asn 12 (O)	3.0
	Arg 130 (NH2)	Sep 11 (O1P)	3.6
	Arg 130 (NH1)	Sep 11 (O2P)	2.7
	Arg 57 (NH1)	Sep 11 (O1P)	3.5
	Arg 57 (NH1)	Sep 11 (O3P)	3.3
	Arg 57 (NH2)	Sep 11 (O3P)	3.0
	Tyr 131 (OH)	Sep 11 (O1P)	2.6
Salt Bridges	Asp 226 (OD1)	Arg 7 (NH1)	3.4
	Asp 226 (OD1)	Arg 7 (NH2)	3.5
	Glu 183 (OE1)	Arg 9 (NE)	2.7
	Glu 183 (OE2)	Arg 9 (NE)	3.3
	Glu 183 (OE1)	Arg 9 (NH2)	3.6
	Glu 183 (OE2)	Arg 9 (NH2)	3.2
	Glu 134 (OE1)	Arg 9 (NH2)	3.4
	Lys 123 (NZ)	Asn 12 (O)	2.7

D

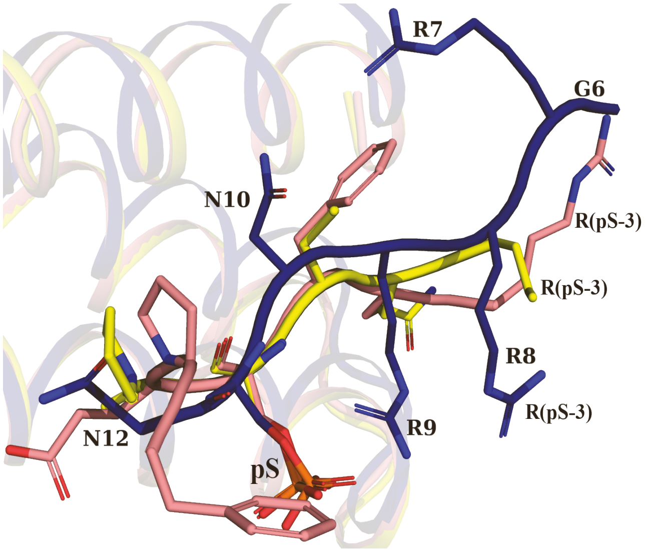

E

Pairs	Pair distance (CZ-CZ) d(Å)	Plane angle (NH1-NH2-NE) ϴ(°)
Arg61-Arg8	3.5	32.6
Arg8-Arg9	4.4	57.4

Supplementary Figure S6: Identification and validation of compound CU7218.

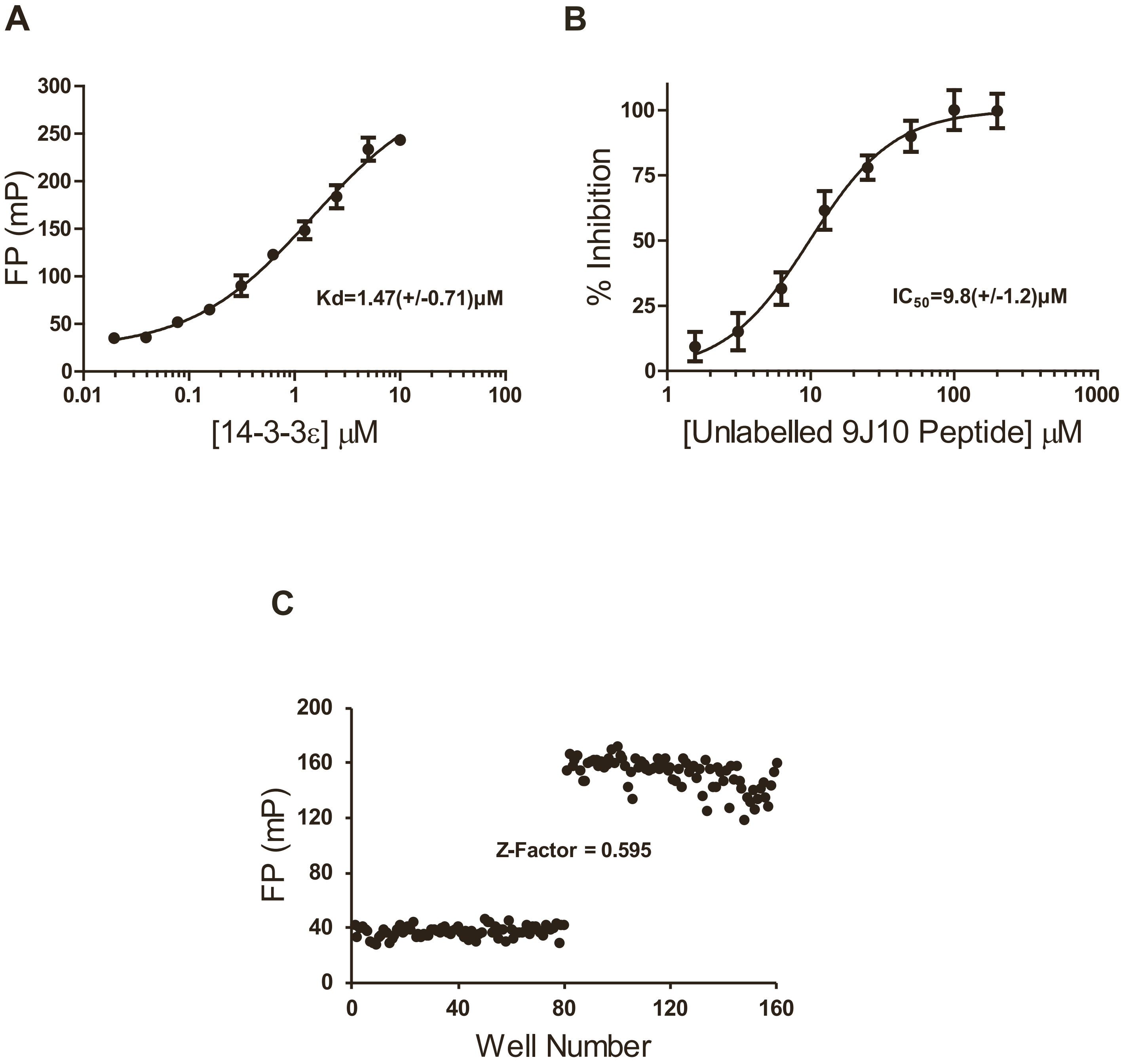

Supplementary Table S1: List of genomes used to generate the peptide library including genome length and percentage representation of each genome within the library (related to STAR Methods).

**Bacteria**	Genome length (bp)	Expected representation	Actual representation
Acinetobacter baumannii	4001457	3.21%	2.82%
Aeromonas hydrophila	4744448	3.81%	8.47%
Bacillus cereus	5432652	4.36%	2.80%
Bacteroides thetaiotaomicron	6293399	5.05%	5.20%
Bordetella pertussis	4086189	3.28%	5.39%
Borrelia burgdorferi	1397488	1.12%	1.65%
Campylobacter jejuni	1641481	1.32%	1.11%
Caulobacter crescentus	4016947	3.22%	4.00%
Chlorobium tepidum	2154946	1.73%	2.02%
Clostridium difficile	4298133	3.45%	2.06%
Corynebacterium diphtheriae	2488635	2.00%	1.36%
Deinococcus radiodurans	3284159	2.64%	2.17%
Desulfovibrio vulgaris	3773159	3.03%	2.82%
Geobacter sulfurreducens	3814128	3.06%	2.21%
Haemophilus influenzae	1830138	1.47%	0.81%
Helicobacter pylori	1667867	1.34%	1.13%
Legionella pneumophila	3397754	2.73%	1.61%
Listeria monocytogenes	2944528	2.36%	1.33%
Mycobacterium tuberculosis	4419977	3.55%	4.94%
Neisseria meningitidis	2194961	1.76%	1.52%
Porphyromonas gingivalis	2343476	1.88%	1.57%
Pseudomonas aeruginosa	6264404	5.03%	4.62%
Rhodobacter sphaeroides	4603060	3.69%	3.73%
Rhodopseudomonas palustris	5467640	4.39%	4.38%
Salmonella enterica	4951371	3.97%	2.43%
Streptomyces avermitilis	8482895	6.81%	5.87%
Thermotoga maritima	1860725	1.49%	1.22%
**Archaea**	Genome length (bp)	Expected representation	Actual representation
Archaeoglobus fulgidus	2178401	1.75%	1.55%
Aeropyrum pernix	1669696	1.34%	1.70%
Haloarcula marismortui	4274642	3.43%	3.69%
Halobacterium	2571010	2.06%	2.54%
Haloferax volcanii	4012900	3.22%	6.58%
Methanocaldococcus jannaschii	1739927	1.40%	0.83%
Pyrococcus horikoshii	1738505	1.40%	0.75%
Sulfolobus solfataricus	2992245	2.40%	1.70%
Thermoplasma volcanium	1584804	1.27%	1.44%

Supplementary Table S2: Validation of 59 primary hits in the FOXO3a Relocalisation Assay in U2OS-GFP-FOXO3a cells (related to Figure 1).

Peptide ID	Mean fold change relative to vector	Mean % cells with nuclear GFP-FOXO3a	Genome of origin
3F14	3.1 +/-1.3	4.6 +/-2.7	*Bordetella pertussis*
17C10	2.6 +/-0.8	4.1 +/-2.8	*Pseudomonas aeruginosa*
24J09	2.5 +/-0.9	3.8 +/-2.1	*Geobacter sulfurreducens*
11H05	2.4 +/-0.6	3.4 +/-1.1	*Rhodopseudomonas palustris*
2E21	2.3 +/-0.5	3.3 +/-0.7	*Aeromonas hydrophila and Legionella pneumophila*
1K05	2.2 +/-0.7	3.0 +/-0.3	*Streptomyces avermitilis*
1F13	2.2 +/-0.6	3.0 +/-0.4	*Bordetella pertussis*
12C03	2.1 +/-0.5	3.1 +/-1.4	*Streptomyces avermitilis*
12L09	2.1 +/-0.4	3.0 +/-0.8	*Bordetella pertussis*
22C04	2.1 +/-0.4	3.1 +/-1.1	*Aeromonas hydrophila*
1O23	2.0 +/-0.7	2.7 +/-0.3	*Unknown*
19I03	2.0 +/-0.6	2.9 +/-1.0	*Rhodobacter sphaeroides*
9J10	2.0 +/-0.3	2.9 +/-0.8	*Streptomyces avermitilis*
1M15	1.9 +/-0.2	3.0 +/-1.3	
20B17	1.9 +/-0.6	2.7 +/-0.7	
18C08	1.9 +/-0.4	2.8 +/-1.3	
14E14	1.9 +/-0.3	2.9 +/-1.4	
18D09	1.8 +/-0.4	2.6 +/-0.9	
18B15	1.8 +/-0.3	2.6 +/-1.2	
19B02	1.7 +/-0.6	2.3 +/-0.4	
18D07	1.7 +/-0.5	2.3 +/-0.2	
17J10	1.7 +/-0.3	2.5 +/-0.9	
11H13	1.6 +/-0.7	2.1 +/-0.2	
17I09	1.6 +/-0.7	2.1 +/-0.6	
17C16	1.6 +/-0.4	2.3 +/-0.5	
18B09	1.6 +/-0.1	2.5 +/-1.0	
3E10	1.5 +/-0.5	2.0 +/-0.2	
18B21	1.5 +/-0.4	2.1 +/-0.4	
7J15	1.5 +/-0.2	2.4 +/-1.2	
24J12	1.4 +/-0.5	1.9 +/-0.4	
11F11	1.4 +/-0.4	1.9 +/-0.4	
3L09	1.4 +/-0.2	2.0 +/-0.7	
12H13	1.3 +/-0.3	1.8 +/-0.4	
10J20	1.2 +/-0.5	1.7 +/-0.5	
19G09	1.0 +/-0.2	1.4 +/-0.4	
Vector	1.0 +/-0.0	1.5 +/-0.7	
10N04	0.9 +/-0.5	1.1 +/-0.2	
3I12	0.9 +/-0.3	1.2 +/-0.2	

10L18	0.9 +/-0.3	1.3 +/-0.2	
3H08	0.9 +/-0.2	1.2 +/-0.2	
5E23	0.9 +/-0.2	1.2 +/-0.3	
21D02	0.9 +/-0.1	1.3 +/-0.4	
3E14	0.8 +/-0.3	1.1 +/-0.2	
3H11	0.8 +/-0.3	1.1 +/-0.1	
8D06	0.8 +/-0.3	1.0 +/-0.2	
3H14	0.8 +/-0.2	1.1 +/-0.2	
6D21	0.8 +/-0.2	1.1 +/-0.3	
7J10	0.8 +/-0.2	1.1 +/-0.3	
11D11	0.8 +/-0.2	1.1 +/-0.2	
14N18	0.8 +/-0.2	1.2 +/-0.2	
8I08	0.8 +/-0.1	1.1 +/-0.4	
3B23	0.7 +/-0.3	0.9 +/-0.3	
5J03	0.7 +/-0.2	1.0 +/-0.2	
6N13	0.7 +/-0.2	1.0 +/-0.2	
7D15	0.7 +/-0.2	1.0 +/-0.3	
7F04	0.7 +/-0.2	1.0 +/-0.2	
8B23	0.7 +/-0.2	1.0 +/-0.2	
3F23	0.7 +/-0.1	1.0 +/-0.3	
10L04	0.6 +/-0.2	0.9 +/-0.1	
14F16	0.6 +/-0.2	0.9 +/-0.2	

Supplementary Table S4. Data collection and refinement statistics of 14-3-3ε:9J10 complex structure (related to Figure 5).

**Parameters**	**14-3-3ε:9J10**
Wavelength (Å)	1.54
Space group	$P2_12_12_1$
Unit cell parameters	
a,b,c (Å)	59.47, 83.35, 118.81
α, β, γ (°)	90 90 90
Resolution (Å)	59.47 – 3.16<br>(3.33 – 3.16)
R_{merge} (%)	14.5 (64.5)
Mean [<I>/σ(<I>)]	8.6 (2.3)
CC (1/2) (%)	99.5 (69.8)
Total no. of reflections	49,648 (6933)
Unique reflections	10, 688 (1516)
Completeness (%)	99.9 (99.5)
Multiplicity	4.6 (4.6)
Molecules in asymmetric unit	2
Solvent content (%)	39.56
**Refinement statistics**	
Resolution (Å)	30.00-3.16 (3.24-3.16)
Rwork (%)	22.17 (30.7)
Rfree (%)	22.05 (36.4)
Mean B-value ($Å^2$)	62.7
Ramachandran favoured (%)	95.9
Ramachandran outlier (%)	0
R.m.s. deviations	
Bond length (Å)	0.003
Bond angle (°)	1.241

Values in parentheses correspond to the highest resolution shell

Supplementary Table S5: List of siRNA target sequences (related to STAR Methods).

siRNA	Supplier	Target Sequence
RICTOR_1	Qiagen	ATGACCGATCTGGACCCATAA
RICTOR_3	Qiagen	TAGGTGCATTGACATACAACA
RICTOR_4	Qiagen	TACGAGCGCTTCGATATCTCA
RICTOR_5	Qiagen	TCCGATCATGGGCAGGTATTA
Non-Targeting Pool #2	Dharmacon	UAAGGCUAUGAAGAGAUAC, AUGUAUUGGCCUGUAUUAG, AUGAACGUGAAUUGCUCAA, UGGUUUACAUGUCGACUAA
PDPK1 pool	Dharmacon	CAAGAGACCUCGUGGAGAA, GACCAGAGGCCAAGAAUUU, GGAAACGAGUAUCUUAUAU, UGGCCAAAUUGCACGGAAU
PDPK1_5	Qiagen	AAGGGCATCATTCACAGGGAC
PDPK1_7	Qiagen	AACAAAGTTCTGAAAGGTGAA
PDPK1_9	Qiagen	CACGCCTAACAGGACGTATTA
XPO1 pool	Dharmacon	GAAAGUCUCUGUCAAAAUA, GCAAUAGGCUCCAUUAGUG, GGAACAUGAUCAACUUAUA, GGAUACAGAUUCCAUAAAU
XPO1_1	Qiagen	CCCATTGTAAAGCGACTTCAA
XPO1_2	Qiagen	TACATGTTACTCCCTAATCAA
XPO1_5	Qiagen	TTCTCAGAATATGAATACGAA
XPO1_6	Qiagen	ATGGTTAGTCGAATGGCTAAA
